# National and subnational incidence, mortality and associated factors of colorectal cancer in China: A systematic analysis and modelling study

**DOI:** 10.7189/jogh.13.04096

**Published:** 2023-10-13

**Authors:** Liying Xu, Jianhui Zhao, Zihan Li, Jing Sun, Ying Lu, Rongqi Zhang, Yingshuang Zhu, Kefeng Ding, Igor Rudan, Evropi Theodoratou, Peige Song, Xue Li, Global Health Epidemiology Research Group (GHERG)

**Affiliations:** 1 Department of Big Data in Health Science, School of Public Health and The Second Affiliated Hospital, Zhejiang University School of Medicine, Hangzhou, China; 2 Colorectal Surgery and Oncology, Key Laboratory of Cancer Prevention and Intervention, Ministry of Education, The Second Affiliated Hospital, Zhejiang University School of Medicine, Hangzhou, China; 3 Centre for Global Health, Usher Institute, University of Edinburgh, Edinburgh, UK; 4 Algebra University, Zagreb, Croatia; 5 School of Public Health and Women's Hospital, Zhejiang University School of Medicine, Hangzhou, China; 6 The Key Laboratory of Intelligent Preventive Medicine of Zheijang Province, Hangzhou. China

## Abstract

**Background:**

Due to their known variation by geography and economic development, we aimed to evaluate the incidence and mortality of colorectal cancer (CRC) in China over the past decades and identify factors associated with CRC among the Chinese population to provide targeted information on disease prevention.

**Methods:**

We conducted a systemic review and meta-analysis of epidemiolocal studies on the incidence, mortality, and associated factors of CRC among the Chinese population, extracting and synthesising data from eligible studies retrieved from seven global and Chinese databases. We pooled age-standardised incidence rates (ASIRs) and mortality rates (ASMRs) for each province, subregion, and the whole of China, and applied a joinpoint regression model and annual per cent changes (APCs) to estimate the trends of CRC incidence and mortality. We conducted random-effects meta-analyses to assess the effect estimates of identified associated risk factors.

**Results:**

We included 493 articles; 271 provided data on CRC incidence or mortality, and 222 on associated risk factors. Overall, the ASIR of CRC in China increased from 2.75 to 19.39 (per 100 000 person-years) between 1972 and 2019 with a slowed-down growth rate (APC_1_ = 5.75, APC_2_ = 0.42), while the ASMR of CRC decreased from 12.00 to 7.95 (per 100 000 person-years) between 1974 and 2020 with a slight downward trend (APC = -0.89). We analysed 62 risk factors with synthesized data; 16 belonging to the categories of anthropometrics factors, lifestyle factors, dietary factors, personal histories and mental health conditions were graded to be associated with CRC risk among the Chinese population in the meta-analysis limited to the high-quality studies.

**Conclusions:**

We found substantial variation of CRC burden across regions and provinces of China and identified several associated risk factors for CRC, which could help to guide the formulation of targeted disease prevention and control strategies.

**Registration:**

PROSPERO: CRD42022346558.

Colorectal cancer (CRC) is a significant global health issue, ranking as the third most common cancer and the second leading cause of cancer-related deaths worldwide in 2018 [1]. It has also become one of the five most common cancers in China since 2020 [2]. China is a large country with great regional variations in economic development, geographic features, as well as medical and health resources [3]. These variations are known to influence exposure to risk factors [4-6] and are crucial for estimating the national CRC burden. China has made efforts to put forward the “Consensus Opinion on CRC screening, early diagnosis and treatment and comprehensive prevention in China” [7-9] and initiated the urban early diagnosis and treatment project to promote early prevention [10,11]. Exploring overall and regional incidence provides a rough measure of population risk and allows for a side-evaluation of the effectiveness of these public health campaigns on CRC prevention. Optimising prevention strategies and allocation of medical resources in high CRC-incidence regions is crucial in reducing the local and national burden of CRC [12]. Investigating the epidemiology of CRC in different regions and provinces can provide important data for adjusting resource allocation to local needs and implementing cost-effective strategies. Given the long transition period from colorectal precancerous lesions to cancer formation [13,14], establishing the profile of associated risk factors is important to understanding some of the drivers of CRC incidence and promoting primary prevention [15]. Current meta-analyses on CRC risk factors combine the results of epidemiological studies from multiple countries [16,17]. Considering the ethnic and country-specific particularities of CRC occurrence [18,19], exploring CRC risk factors in the Chinese population could help with implementing targeted intervention measures. While previous studies have examined the epidemiology and aetiology of CRC in the Chinese population [20-26], few examined the incidence and mortality of CRC or the factors associated with its occurrence. We aim to address this gap by providing a comprehensive overview of the national and subnational CRC incidence, mortality, and trends of CRC, and the associated risk factors in China.

## METHODS

We registered the protocol in the International Prospective Register of Systematic Reviews (PROSPERO) (CRD42022346558) and conducted this systematic review and meta-analysis following the Preferred Reporting Items for Systematic Reviews and Meta-Analyses (PRISMA) [27] and the Meta-analysis of Observational Studies in Epidemiology (MOOSE) guidelines [28].

### Data sources

We obtained data on CRC incidence, mortality, and risk factors from a literature search. For incidence and mortality data, we conducted a secondary analysis by searching for published studies based on data from cancer registries of the Centers for Disease Control and Prevention (CDC) across China, as well as their chronic disease surveillance system, tumour follow-up registry, cause of death surveillance registration system, and cause of death retrospective investigation. We also evaluated data from the Global Burden of Disease (GBD) 2019 study and the Global Cancer Observatory (GLOBOCAN) 2020 online databases [29-33] for comparison. GLOBOCAN 2020, a project sponsored by the International Agency for Research on Cancer (IARC), provides multidimensional data on the incidence, mortality, and prevalence of 36 diseases in 185 countries and territories. Data on trends of CRC incidence in China between 1988 and 2012 were visualised in the GLOBOCAN “Cancer over time” database [34], with excluded mortality data. GBD 2019 estimates multidimensional data on 369 injuries and diseases from 204 countries and territories since 1990. Data on CRC incidence and mortality in China between 1990 and 2019 were available in the GBD 2019 online database [35].

### Literature search

We systematically searched for observational studies on the incidence, mortality, or associated risk factors of CRC in the native Chinese population from three Chinese (China National Knowledge Infrastructure (CNKI), Wanfang, Chinese Biomedical Literatures database (CBM-Sino Med)) and four global databases (MEDLINE, Embase, Web of Science, and Scopus) (Table S1 in the **Online Supplementary Document**). We additionally searched the reference lists of included studies and previous meta-analyses on CRC risk factors for any other studies conducted on the Chinese population. The search had no language restrictions and ended on 14 June 2023.

### Inclusion and exclusion criteria

We included studies reporting statistics on CRC incidence, mortality, or risk factors associated with CRC in the Chinese populations, cohort and case-control studies (including case-cohort and nested case-control studies) on risk factors associated with CRC development, and studies reporting age-standardised incidence rates (ASIRs) and age-standardised mortality rates (ASMRs) of CRC based on cancer registries or CDC. For repeated publication of the same study, we chose the one with the most comprehensive information. The Chinese population in our study refers to Chinese residents of all ethnicities in the mainland, Hong Kong, Macao, and Taiwan province, excluding overseas Chinese. We excluded abstracts, letters, case reports, case series, reviews, meta-analyses, studies on CRC recurrence, survival, prognosis, treatment or genetic factors, and studies with fewer than ten incident cases. We only included factors mentioned and analysed in at least three studies. Studies were included two researchers (JZ and LX) reached consensus on their eligibility.

### Data extraction

We extracted the following information: author, publication year, investigation period, study design, province and location, cancer site, participants (sample size, sex, number of males and females, mean age), incidence or mortality rate of CRC and data on associated factors (and their definition), odds ratios (ORs), relative risks (RRs), or hazard ratios (HRs), and corresponding 95% confidence intervals (95% CIs).

We categorised the study regions based on the definitions of the National Bureau of Statistics of China, with six areas set according to geography (East China, North China, Northeast China, Northwest China, South Central China and Southwest China) and four according to economic levels (Northeast region, East region, Central region and West region) [36,37] (Table S5 in the **Online Supplementary Document**).

### Study quality assessment

We used a modified Newcastle-Ottawa Scale (NOS) [38] to assess the quality of case-control and cohort studies. We pooled eight questions belonging to three sections in the NOS and categorised the quality assessment score of each study as low quality (0-3), medium quality (4-6), and high quality (7-9). Two authors (JZ and LX) independently conducted the assessment and resolved any discrepancies by further discussion until consensus was reached (Tables S2-S3 in the **Online Supplementary Document**).

### Statistical analysis

#### Pooling ASIR and ASMR of CRC and trend analysis at national, regional, and provincial levels

We collected data reported on a national level, data from cancer registries, and data from the provincial, city-level, and county CDCs in China. We performed pooling analyses on incidence or mortality data to capture the epidemiological characteristics of CRC in China at different geographical levels. To accurately estimate the regional disease burden, we first summarised and estimated the provincial CRC epidemiological data, then integrated them into regional data, and finally, estimated the national CRC epidemiological data. We examined the time trends of ASIRs and ASMRs for CRC at national and subnational levels in China through a joinpoint regression model [39] in segments. The final model provided the annual percentage change (APC) of the rates over time to reflect the direction and magnitude of trends. When the slope of the trend was statistically different from zero (*P* < 0.05), we described it as “decreasing significantly” or “increasing significantly”. We also evaluated trends of CRC incidence and mortality in China using publicly available data from the GLOBOCAN 2020 [34] and GBD 2019 [35] online databases for comparison.

#### Meta-analyses of the reported factors associated with CRC risk

We only pooled the effect estimate of risk factors assessed by at least three studies in the overall analysis. For each risk factor, we used the random-effects model (DerSimonian Laird method) to estimate the summary effect estimate and tested for heterogeneity (heterogeneity defined as *I^2^*>50%, *P* < 0.05) among included studies by Cochran’s Q statistic and *I^2^* metric (95% CI). We applied subgroup meta-analyses by sex (male vs female), and tumour site (colon vs rectum). We excluded studies with a low or moderate quality rating from the sensitivity analysis; for factors that were explored in both case-control studies and cohort studies, we conducted further sensitivity analyses for cohort studies. We assessed potential publication bias with Begg's and Egger's tests. We further applied excess significance test to explore whether the observed number of studies with significant results differed from the expected number of significant studies using the χ^2^ test (*P* < 0.1) and the Egger's regression test to evaluate the small-study effects (*P* < 0.1).

Except for the excess significance test and the small-study effects test, we considered a two-sided *P*-value of <0.05 as statistically significant. We performed all statistical analyses using Stata, version 13.0 (StataCorp LLC, College Station, Texas, USA) and the “meta” package in R, version 4.0.2 (R Core Team, Auckland, New Zealand). We created the maps of China through by ArcMap, version 10.7.0 (Esri, Redlands, California, USA) using the Chinese base map obtained from the DataV-GeoAtlas [40].

### Evidence credibility grading

As described previously [41], we applied credit assessment to assess the evidence credibility of modifiable risk factors and divided into five categories according to pre-defined criteria (Table S4 in the **Online Supplementary Document**). We categorised the credibility of evidence with significant results (*P* < 0.05) into four categories: class I, II, III, and IV.

## RESULTS

### Summary of systematic review

**Figure 1:**
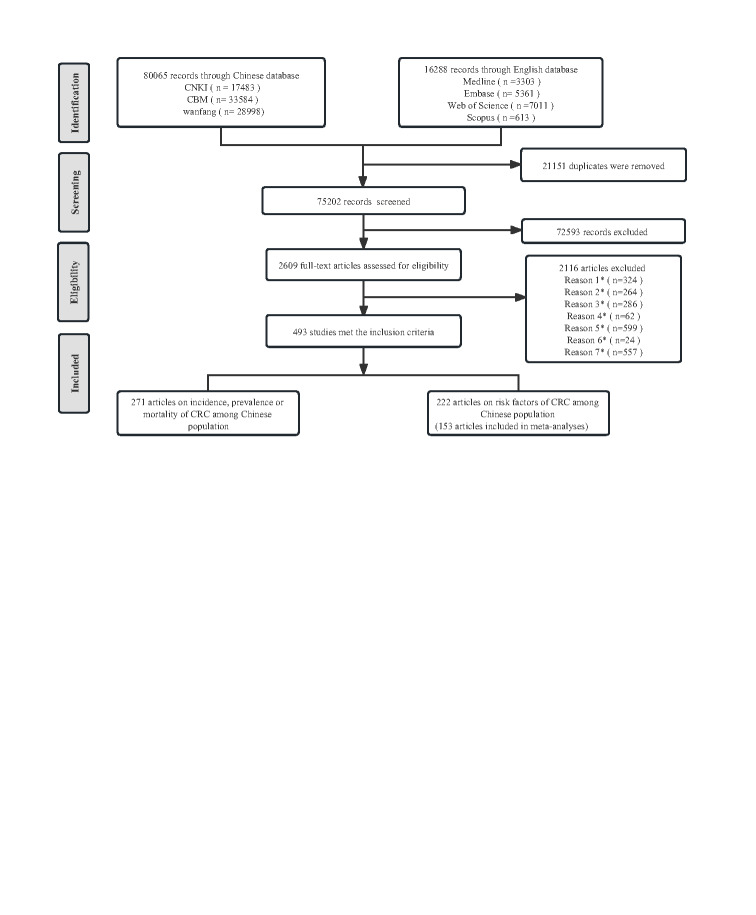
Study selection and flowchart. Reason 1*: Reviews or other non-original studies, Reason 2*: Meeting reports, Reason 3*: Studies that are not population-based or not based in China, Reason 4*: Full text not found, Reason 5*: No relevant indicators were mentioned (incidence /mortality rates or risk factors for Colorectal cancer), Reason 6*: Multiple publications of the same study, Reason 7*: Not standardised or not standardised according to uniform standards. CNKI – China National Knowledge Infrastructure, CBM – China Biology Medicine disc, CRC – colorectal cancer.

We retrieved 96 353 records from the database searches, 80 065 from Chinese and 16 288 from the global databases. Following deduplication and independent screening of titles, abstracts, and full texts by two reviewers, we included 493 eligible studies; 271 reported on ASIR and ASMR for CRC, and 222 reported associated risk factors for the Chinese population (**Figure 1**). Studies reporting on ASIR and ASMR (per 100 000 person-years) of CRC were all population-based, covering all six geographic regions and four economic areas in China (Table S5 in the **Online Supplementary Document**). The detailed regional distribution and characteristics of included studies are presented in Table S6-S7 in the **Online Supplementary Document**. We analysed trends of age-standardised rates summarised from the pooled analysis in the joinpoint regression model (**Table 1**).

**Table 1:** Trends analysis on ASIR and ASMR of CRC in different regions of China

		APCs, segment_1_		APCs, segment_2_		APCs, Segment_3_	
	**Period**	**Year**	**APC (%)**	**Year**	**APC (%)**	**Year**	**APC (%)**
**ASIR by geographical region**							
Northeastern China	2008-2017	2008-2017	-2.03	-	-	-	-
North China	2010-2019	2010-2019	5.03*	-	-	-	-
Eastern China	1993-2018	1993-2008	5.61*	2008-2018	-0.73	-	-
South Central China	1972-2019	1972-2006	6.38*	2006-2017	-1.71	2017-2019	33.23
Northwestern China	2009-2018	2009-2018	6.18*	-	-	-	-
Southwestern China	2010-2019	2010-2019	1.55	-	-	-	-
**ASMR by geographical region**							
Northeastern China	2001-2017	2001-2007	8.28*	2007-2017	-3.72*	-	-
North China	2003-2017	2003-2008	9.69*	2008-2013	-8.12	2013-2017	6.45
Eastern China	1974-2018	1974-2018	-1.00*	-	-	-	-
South Central China	2005-2018	2005-2018	-0.06	-	-	-	-
Northwestern China	2009-2020	2009-2020	7.10*	-	-	-	-
Southwestern China	2005-2019	2005-2019	4.72*	-	-	-	-
**ASIR by economic region**							
Northeastern China	2008-2017	2008-2017	-2.03	-	-	-	-
East China	1972-2019	1972-2006	5.74*	2006-2019	1.61	-	-
Central China	2010-2018	2010-2018	2.78*	-	-	-	-
Western China	2009-2019	2009-2019	5.01*	-	-	-	-
**ASMR by economic region**							
Northeastern China	2001-2017	2001-2007	8.28*	2007-2017	-3.72*	-	-
East China	1974-2018	1974-1981	10.78*	1981-2018	-1.58*	-	-
Central China	2005-2018	2005-2018	1.16	-	-	-	-
Western China	2005-2020	2005-2020	3.22*	-	-	-	-
**ASIR for all of China**	1972-2019	1972-2006	5.75*	2006-2019	0.42	-	-
**ASMR for all of China**	1974-2020	1974-2020	-0.89*	-	-	-	-

ASIR – age-standardised incidence rate, ASMR – age-standardised mortality rate, CRC – colorectal cancer, APC – annual percentage change

*P < 0.05.

### ASIR and ASMR of CRC and trends in China

**Figure 2:**
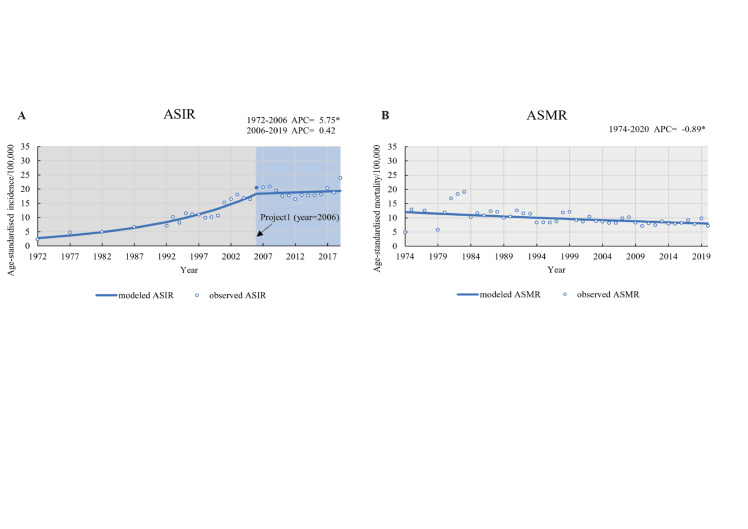
Trends analysis on ASIR and ASMR of CRC in entire China. **Panel A.** Yearly age-standardised incidence (1972-2019) of colorectal cancer in China based on the joinpoint regression model. **Panel B.** Yearly age-standardised mortality rate (1974-2020) of colorectal cancer in China based on the joinpoint regression model. The solid dot indicates a joinpoint (turning point demarking significance). The age composition of the Chinese population in 2000 was used as the standard population. . An asterisk (*) symbol indicates *P* < 0.05. ASIR – age-standardised incidence rate, ASMR – age-standardised mortality rate, CRC – colorectal cancer, APC – annual percentage change.

ASIR of CRC increased from 2.75 to 19.39 (per 100 000 person-years) between 1972 and 2019 (APC_1_ = 5.75 (*P* < 0.05), APC_2_ = 0.42) with a slowed-down growth rate (**Figure 2**). The pooled ASMR of CRC declined from 12.00 to 7.95 per 100 000 person-years in China from 1974 to 2020 (APC = -0.89 (*P* < 0.05)). We also found this upward trend in ASIR in the trend analysis of GBD-source data, but observed inconsistent results in the trend of ASMR (Figure S1 in the **Online Supplementary Document**). We compared the trends of CRC ASIR and ASMR in China obtained from our meta-analysis with data from the GBD database and GLOBOCAN 2020 online database (Figure S2 in the **Online Supplementary Document**).

**Figure 3:**
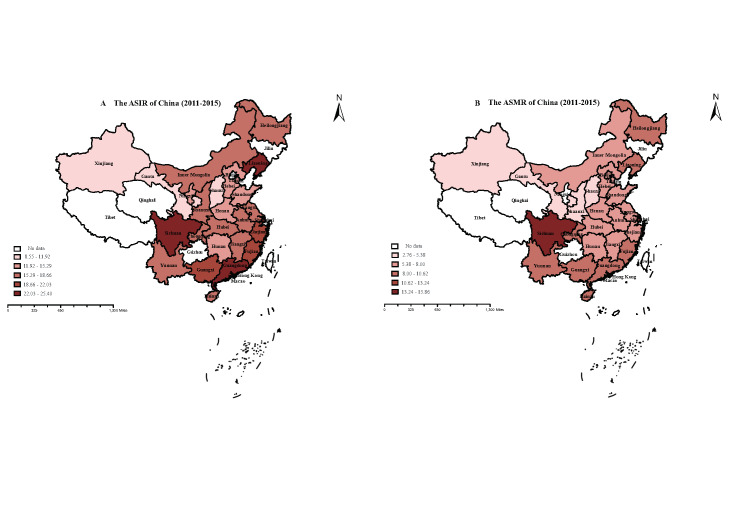
The ASIR and ASMR of CRC in China between 2011 and 2015. **Panel A.** Age-standardised incidence rate (ASIR) of colorectal cancer for each province in China, 2011-2015. **Panel B.** Age-standardised mortality rate (ASMR) of colorectal cancer for each province in China, 2011-2015. The age-standardised rate was divided into five segments. The colour gradient represents the magnitude of the age-standardised rate, the darker the colour, the larger the rate. Provinces with no data are filled in with blank spaces. ASIR – age-standardised incidence rate, ASMR – age-standardised mortality rate, CRC – colorectal cancer.

To analyse the incidence and mortality data of CRC at the subnational level, we divided China into six geographical and four economic regions (Table S5 in the **Online Supplementary Document**). The trends of CRC incidence and mortality were not the same in different regions (**Table 1**). Geographically, Northeast and East China had insignificant downward trends in CRC ASIR since 2008, while North, Northwest, and Southwest China showed upward trends. South Central China's CRC ASIR fluctuated. ASMR exhibited significant upward trends in Northeast, North, Northwest, and Southwest China since the 21st century. However, since 2007, Northeast China's ASMR has tended to decline, while North China's ASMR fluctuated. We observed a steady downward trend in Eastern China since 1974 (Figure S3 in the **Online Supplementary Document**). According to the economic partitions (Figure S4 in the **Online Supplementary Document**), the ASIR of CRC decreased in the Northeast region (APC = -2.03), had a slowed-down upward trend in the East region (APC_1_ = 5.74 (*P* < 0.05), APC_2_ = 1.61), and increased in the Central region (APC = 2.78 (*P* < 0.05)) and West region (APC = 5.01 (*P* < 0.05)). ASMR decreased significantly in the Northeast (APC_1_ = 8.28 (*P* < 0.05), APC_2_ = -3.72 (*P* < 0.05)) and East regions (APC_1_ = 10.78 (*P* < 0.05), APC_2_ = -1.58 (*P* < 0.05)) and tended to increase slightly in the Central region (APC = 1.16), with a significant upward trend in ASMR in the Western region (APC = 3.22 (*P* < 0.05)). The estimated pooled provincial ASIR and ASMR (per 100 000 person-years) of CRC from 2011 to 2015 are presented in **Figure 3**. ASIR and ASMR varied widely across provinces, among which those in Liaoning, Shanghai, Guangdong, Sichuan and Guangxi were ranked the top in China, while these in Xinjiang, Shanxi, and Gansu were relatively low.

### Spectrum of CRC-associated factors among the Chinese population

**Figure 4:**
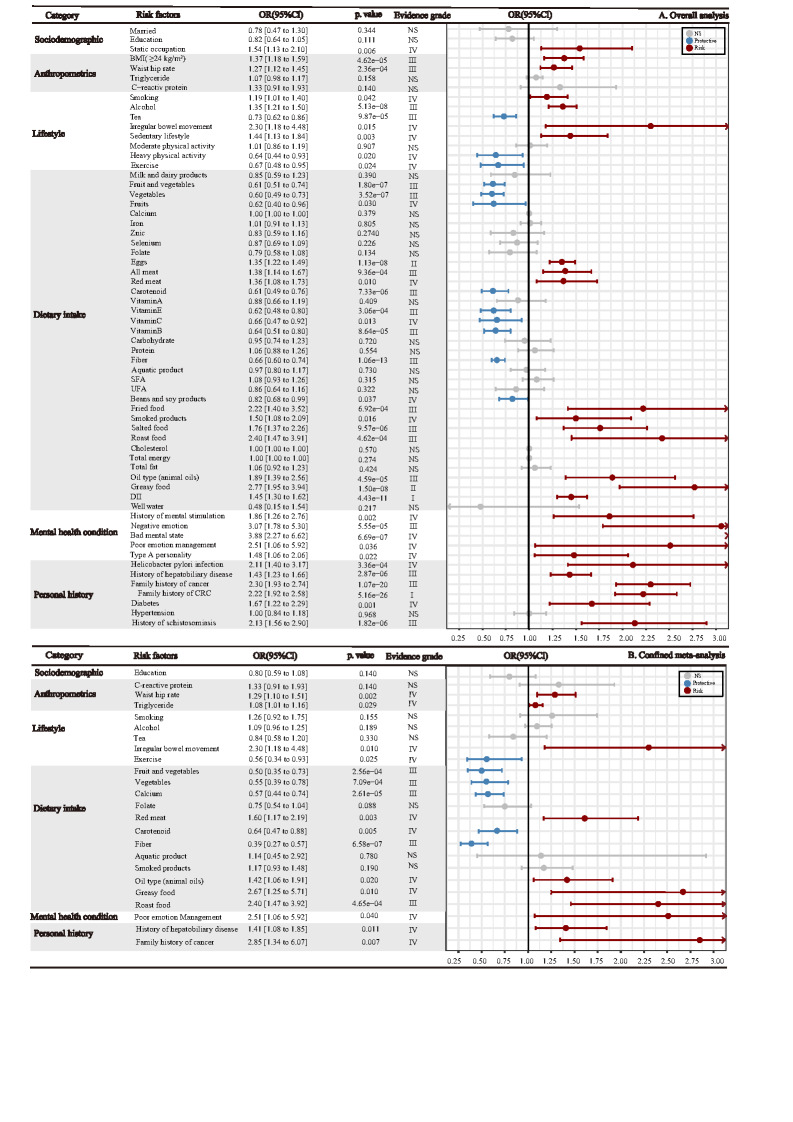
Results of overall meta-analysis confined meta-analysis of high-quality studies on the association between environmental factors and CRC. **Panel A.** Results of the overall meta-analysis on the association between environmental factors and CRC. **Panel B.** Results of the confined meta-analysis of high-quality studies on the association between environmental factors and CRC. All the factors are classified into various categories: sociodemographic, anthropometrics, lifestyle, dietary intake, mental health condition, and personal history factors. Three different colored legends represent different three different associations. Grey represents no significant association, blue represents a negative correlation, indicating to be a protective factor, and red indicates a positive correlation, indicating to be a risk factor. Evidence class criteria: Convincing (class I): statistical significance with *P* < 1 × 10^−6^; included more than 1000 cases; *I^2^*<50%; 95% prediction intervals excluding the null value; no evidence of small study effects (*P* > 0.10) and excess significance bias (*P* > 0.10). Highly suggestive (class II): statistical significance with *P* < 1 × 10^−3^; included more than 1000 cases; the largest component study reporting a significant result (*P* < 0.05). Suggestive (class III): statistical significance with *P* < 1 × 10^−3^; included more than 1000 cases. Weak (class IV): statistical significance with *P* < 0.05. Non-significant: *P* > 0.05. CRC – colorectal cancer, BMI – body mass index, UFA – unsaturated fatty acids, SFA – saturated fatty acids, DII – dietary inflammatory index, NS – non-significant, OR – odds ratio, CI – confidence interval.

We included 62 factors for analysis; 40 were identified as associated with CRC risk in the overall and 16 in the confined meta-analysis limited to high-quality studies (**Figure 4**). Subgroup analyses by tumour site and sex are presented in Tables S8-S9, the results of the confined meta-analyses for cohort studies in Table S10, the full list of studies included in each factor in Table S11, and summary information specific to each factor included in the overall and confined meta-analysis of high-quality studies in Tables S12-S13 in the **Online Supplementary Document**.

#### Sociodemographic factors

Fourteen studies investigated the association between sociodemographic factors (marital status, education level, and occupation) and risk of CRC. A static occupation was found to have a significant positive association with increased CRC risk in our overall analysis (OR = 1.54; 95% CI = 1.13-2.10) and in the confined meta-analysis of cohort studies. We found no significant association between education level, marital status, and CRC risk, even when analysing cohort or high-quality studies (**Figure 4** and Table S10 in the **Online Supplementary Document**).

#### Anthropometrics factors

We identified ten articles investigating the relationship between BMI and CRC risk and found that high BMI (BMI≥24 kg/m^2^) was significantly associated with increased risk of CRC (pooled OR = 1.37; 95% CI = 1.18-1.59) (**Figure 4**). Higher WHR was strongly associated with higher CRC risk (OR = 1.27; 95% CI = 1.12-1.45) in the overall analysis. This positive correlation persisted in the analysis limited to cohort studies, where we also observed a positive association between triglyceride (TG3) and CRC risk (Table S10 in the **Online Supplementary Document**).

#### Lifestyle and dietary factors

Three studies investigating the association between irregular bowel movement and the CRC risk and meta-analysis showed a positive association (OR = 2.30; 95% CI = 1.18-4.48), even when we limited the analysis to high-quality studies (**Figure 4**). In overall analysis, we observed that smoking (OR = 1.19; 95% CI = 1.01-1.40) and alcohol consumption (OR = 1.35; 95% CI = 1.21-1.50) were positively associated with increased CRC risk, while tea consumption (OR = 0.73; 95% CI = 0.62-0.86) and exercise (OR = 0.67; 95% CI = 0.48-0.95) showed inverse associations with CRC risk.

We included 81 articles on the associations between dietary factors and CRC risk in the overall analysis (**Figure 4**). All information on dietary intake was obtained through face-to-face questionnaires and compared between the highest and lowest dose categories. We identified 19 factors as significantly associated with CRC risk in the overall analysis, but nine when limiting the analysis to high-quality studies, eight of which have been reported in the overall analysis except for dietary calcium intake. The overall analysis did not find a significant association between dietary calcium intake and CRC risk, but we observed a significant inverse association was observed in the confined meta-analysis of three high-quality studies (OR = 0.57; 95% CI = 0.44-0.74). In a sensitivity analysis of only high-quality studies, intake of vegetables (OR = 0.55; 95% CI = 0.39-0.78), total fruit and vegetable (OR = 0.50; 95% CI = 0.35-0.73), dietary fibre (OR = 0.39; 95% CI = 0.27-0.57), and carotenoid (OR = 0.64; 95% CI = 0.47-0.88) showed significant inverse associations with CRC, while intake of red meat (OR = 1.60; 95% CI = 1.17-2.19), animal oil (OR = 1.42; 95% CI = 1.06-1.91), greasy food (OR = 2.67; 95% CI = 1.25-5.71), and roast food (OR = 2.40; 95% CI = 1.47-3.92) significantly increase the risk of developing CRC. We found no significant association between aquatic product intake and CRC risk in either the overall analysis or the confined meta-analysis of high-quality studies. We only observed the positive association between the high intake of milk and dairy products and the increased CRC risk in rectal cancer (Table S8 in the **Online Supplementary Document**).

#### Personal and family disease history

We conducted a confined meta-analysis of high-quality studies on the association between personal and family disease history and CRC risk on ten studies (**Figure 4**). Here, we identified four studies investigating the association between a history of hepatobiliary diseases and CRC risk, finding that a disease history of hepatobiliary contributed to an increased risk of CRC (OR = 1.41; 95% CI = 1.08-1.85), which remained true for the overall meta-analysis and the analysis limited to cohort studies (Table S10 in the **Online Supplementary Document**). Additionally, a family history of cancer remained significantly associated with increased CRC risk in the confined meta-analysis of six high-quality studies (OR = 2.85; 95% CI = 1.34-6.07).

#### Mental health conditions

Besides the widely reported risk factors, we found psychological problems to be associated with increased CRC risk among the Chinese population, as we identified an association between poor emotional management and increased CRC risk in both the overall analysis and the confined meta-analysis of high-quality studies (OR = 2.51; 95% CI = 1.06-5.92) (**Figure 4**).

### Assessment of evidence grading

We assessed the credibility levels of identified associations based on our classification evaluation criteria (Table S4 in the **Online Supplementary Document**). When exploring CRC-associated risk factors, ten (16%) meta-analyses showed a *P* < 10^−6^, 56 (90%)) had more than 1000 cases, 13 (21%) had no large heterogeneity (*I^2^*<50%), and 33 (53%) had neither small-study effects nor excess significance bias (Tables S12-13 in the **Online Supplementary Document**). Based on the results of the evidence assessment (**Figure 4**), two factors showed convincing evidence (high dietary inflammation index (DII), family history of CRC); two showed highly suggestive evidence (eggs and greasy food intake), 19 showed suggestive evidence (alcohol intake, fruit and vegetables intake, fibre intake, etc.), and 17 showed weak evidence (static occupation, irregular bowel movement, red meat intake, history of *Helicobacter pylori* infection, etc.) on the association with CRC risk.

## DISCUSSION

To the best of our knowledge, this is the first systematic review and meta-analysis to comprehensively assess the trends in CRC incidence and mortality over the past decades, and identify associated risk factors in China. Here we present three key findings. First, the overall increase in the ASIR of CRC slowed down and the ASMR showed a moderate gradual decline in China since the 1970s. Second, the incidence and mortality rates of CRC vary widely among regions and provinces in China, highlighting the need for attention. Third, several factors, including unhealthy diet and lifestyle, personal or family disease history, and psychological problems are all associated with an increased CRC risk. Given the current substantial and inequitable regional disease burden and complex aetiological networks, further region-specific policies should be formulated to promote disease prevention, identify local epidemiological characteristics, and comprehensively manage modifiable risk factors. Policies will also need to improve early screening techniques and strategies, follow diagnosis and treatment guidelines, and strengthen disease control in order to reduce the overall disease burden and narrow regional disparities.

### Disease burden of CRC in China

China is facing growing challenges in addressing its CRC disease burden [42,43]. Population-based incidence and mortality data serve to inform national and regional cancer prevention and control efforts, thus necessitating a comprehensive assessment of regional and national cancer burden and time trends. Previous studies have found and elaborated on discrepancies in cancer epidemiological data estimations between GBD and GLOBOCAN [44]. Current assessments of CRC incidence and mortality, and their trends in China by GBD 2019 and GLOBOCAN 2020 are not completely consistent and regional-level data are not available, making further studies particularly meaningful. The estimates of country-specific, national- and regional-level CRC incidence and mortality and related time trends assessed here fill this gap.

We found that the ASIR of CRC initially increased steeply, but gradually slowed down, showing an overall upward trend between 1972 and 2019. This slowdown might be attributed to the long-term efforts of the national public health measures, such as strengthening screening and disease prevention and control measures [45], promoting preventive education programs to increase the health awareness of the population and their active participation in screening [46,47], increasing the supply of national health resources [48], and enhancing early detection [49], which have been reported to be associated with improved cancer management. Nowadays, measures such as cancer preventive education and screening of high-risk groups have been highlighted in the Health China Action: Cancer Prevention and Control Implementation Plan and Healthy China 2030 Strategy [50,51]. Thanks to the joint efforts of the government and health organisations, screening, diagnostic capacity, and health education in China have greatly improved [52-54]. For example, the coverage of the Cancer Screening Program in Urban China had increased from six cities in nine provinces in 2012 to 67 cities in 28 provinces in 2021 [26]. Besides, some novel diagnostic options such as real-time automatic detection systems and cell-free DNA (DNA) methylation-based models implemented in some hospitals around China have visibly enhanced the early detection of colorectal lesions [55,56]. Surprisingly, possibly as a result of health education, the current Chinese population has a positive attitude towards screening and early diagnosis of CRC [57].

However, we observed a slight downward trend of ASMR for CRC in China, consistent with the results of the National Mortality Surveillance System-based study between 2005 and 2020 [58]. The level of medical and health services supply in China increased significantly from 2005 to 2020 [59]. Additionally, extensive screening helps to reduce CRC mortality and promote early detection [60,61]. Improvements in the early detection rate, as well as basic medical and improved treatment capacity, also lead to advances in the prognosis of CRC [62,63] and to a corresponding downward trend in the ASMR of CRC. Thus, this subtle downward trend may be linked to extensive screening, early diagnosis and treatment, and enhanced availability of medical resources [26,64].

During 1974-2020, the level of CRC ASIR nationwide was lower than the result estimated by the GBD 2019 (30.55 per 100 000 in 2019); and GLOBOCAN 2020 (23.9 per 100 000 in 2020) [65,66]. Similarly, the level of CRC ASMR nationwide between 1972 and 2019 was lower than the estimated by GBD (13.86 per 100 000 in 2019) and GLOBOCAN 2020 (12.0 per 100 000 in 2020) [65,66]. Different data sources and estimation methodologies may account for the disparities [67,68].

There are also regional variations in the development of CRC incidence and mortality across China. Our results indicate that, since approximately 2008, the ASIR and ASMR of CRC tended to decline in Northeast China and East China, while in some regions, particularly West China, the disease burden of CRC remains heavy, with a significant upward trend of ASIR and ASMR. This regional variation may be related to the unequal distribution of health care resources [69] and the gap in health literacy [70]. The enhanced supply of health resources has positive implications for population health [71], while low health literacy may result in under-utilisation of health resources and increased death risk [72]. Recently, with the promotion of national propaganda and education, the health literacy level of residents in the western region and the supply of medical resources have been continuously optimised, but still lag behind those in other regions [46,70,73]. This highlights the urgency of optimising the allocation of medical resources and promoting screening, prevention, and treatment in Western regions. Given the current significant burden of CRC in China [43] and the variations of CRC incidence and mortality across the country [74], there is a need to better control CRC and reduce its burden, as well as narrow the gaps between regions. The health system must continue to enhance disease surveillance and health education, increase the availability of health resources, strengthen medical structures, and improve the diagnostic and treatment capacities of local hospitals. Moreover, it is important to optimise the allocation of medical resources and infrastructure construction across China, especially in West China, to further reduce the rising trend of CRC incidence and effectively address this disease burden.

### CRC-associated factors among Chinese populations

Early prevention of CRC is a cost-effective way of combating cancer and reducing health care costs [75]. An essential aspect of early prevention is identifying and targeting risk factors [15]. Besides commonly reported related factors, we also found some less-reported potential associated factors of CRC, all of which could lay the theoretical foundation for primary and secondary prevention of CRC, risk prediction, and anchoring of at-risk populations, and should be explored in future studies.

Besides the many lifestyle factors associated with risk of CRC [76-86], we found a positive association between the frequency of more than one bowel movement per day (compared with once per day) and the CRC risk in the Chinese population. Studies on the association between bowel frequency and CRC risk have not yielded consistent results [87-90]. In a previous meta-analysis of ten cohort studies worldwide, increased frequency of bowel movements was only linked to an increased risk of rectal cancer [91]. The results were inconsistent among included studies, where a follow-up study in the Chinese population showed that those who had more than one bowel movement per day had an increased risk of CRC within five years of follow-up, while three studies in the USA and one in Japan did not show an association between bowel frequency and CRC risk [91]. Our meta-analysis included two case-control studies and one cohort study, all of which reported a significant positive association between high bowel frequency (more than once per day) and an increased CRC risk among the Chinese population. This inconsistency may be due to the limited number and different types of included studies, but it also suggests that increased frequency of defecation might be a potential risk factor and early warning signal specific to the Chinese population. More research is needed to further clarify this supposed correlation between bowel frequency and CRC risk.

Poor diet is a major contributor to CRC risk and has become a growing concern [92]. Our results are in line with those of other studies, which indicated that a high intake of meat was associated with a higher risk of CRC [93-95], while a high intake of fruits and vegetables [96,97], fibre [98,99], and legumes [100,101] was associated with a lower risk. Vitamins are known to have a strong antioxidant effect and can reduce free radical damage to the body [102]. However, existing studies have not drawn consistent conclusions about the association between vitamin supplementation and CRC risk [103-105]. When pooling a small number of studies, our overall meta-analysis showed significant negative associations of CRC risk with carotenoid, and vitamin E, C, or B intake, but no association with vitamin A intake. Given the small number of studies we included and the currently inconclusive results, more follow-up studies are needed to verify this association. Due to the vast territory and diverse landscape, the types of edible oil consumed vary around the country. Soybean, peanut, or cottonseed oil, as well as ghee and lard, are all commonly consumed throughout China [106]. Different oils have different proportions of substance and effectiveness [107]. With few studies focused on the role of animal oils in CRC development, the relationship between the two is still a blur [108-110]. We found that high consumption of animal oils in the Chinese population was associated with an increased risk of CRC, suggesting it could be controlled by moderately reducing animal oil intake.

The influence of psychological factors on the incidence of CRC has been explored in a few studies with uncertain results [111-113]. Although previous studies have focused on the effects of cancer on mental health instead of the reverse [114], we did find several mental health factors contribute to a higher risk of CRC in the Chinese population. The associations between psychological health status, especially the poor ability of emotion regulation and the increased CRC risk, were prominent among the Chinese population. Studies demonstrated that suppressing negative emotions is negatively correlated with physical and mental health [115,116]. Consequently, we should be attentive to mental health issues, learn to adjust, and conduct proper emotional management in our daily lives to reduce the risk of CRC.

### Study strengths and limitations

Our study had several important strengths. First, it synthesised various data sources to efficiently and fully demonstrate the different levels of the CRC burden in China, improving the public understanding of its epidemic dynamics. Second, all included studies were carried out in the Chinese population, ensuring that the identified associated factors apply to the actual population of China and can be targeted for CRC prevention in this context. Furthermore, we not only assessed data on epidemiological characteristics and country-specific associated factor profiles for China as a whole, but also further refined the regional burden of disease.

Our study also has some limitations. Caution should be exercised when drawing conclusions from the estimated trends of CRC incidence in all of China, given the low number of studies conducted between the 1970s and 1990s. Additionally, in the sub-regional analysis of CRC burden, the proportion of research conducted in the eastern region is significantly higher than that in the other regions; this uneven distribution could have biased our results and led to a lack of representativeness. Moreover, due to the data distribution, we chose 2011-2015 as the research period to cover as many provinces as possible when analysing provincial ASIR and ASMR of CRC, and could therefore not provide evaluations for more recent periods. Futures studies on regional disease burden should more comprehensively assess the regional disease burden of CRC. We also found a high degree of heterogeneity in our studies, which might be related to their different designs. Regarding CRC-related factors, most of the studies we included were of medium quality, which led to the loss of many factors due to the insufficient number of studies in conducting meta-analysis focusing only on high-quality studies. Here we focused on fragmented risk factors, and although we grouped them into several broad categories, most of them revolve around lifestyle elements that are difficult to categorise accurately. Due to the lack of information available, we were unable to do a more detailed subgroup analysis of the anatomical locations of CRC. Additionally, the uneven composition of risk factors per study may have caused biases.

## CONCLUSIONS

We found substantial variations in CRC burden across different regions and provinces in China and highlighted areas with the heaviest disease burden. We identified a wide range of factors associated with CRC risk in Chinese populations, which could help guide the formulation of targeted disease prevention and control strategies, the rational adjustment of medical and health resource allocation, and improved responses to challenges.

## Additional material


Online Supplementary Document
