# Supplementary material for: National and subnational incidence, mortality and associated factors of colorectal cancer in China: A systematic analysis and modelling study
Source: J Glob Health. 2023 Oct 13;13:04096. doi: 10.7189/jogh.13.04096 (PMC10569376; doi:10.7189/jogh.13.04096)

Table S1 Search strategy to identify studies reporting the ASIR, ASMR and risk factors for CRC in China.

Table S2 Quality assessment of the included case-control study.

Table S3 Quality assessment of the included cohort study.

Table S4 Evidence credibility grading criteria.

Table S5 Regional distribution of included studies.

Table S6 The number of the included studies among different regions.

Table S7 Characteristics of the included studies.

Table S8 Results of subgroup analysis of risk factors for CRC by site.

Table S9 Results of subgroup analysis of risk factors for CRC by sex.

Table S10 Results of Meta-analysis of CRC Risk Factors (restricted to cohort studies).

Table S11 Full-list of the included studies on the risk factors for CRC in China.

Table S12 Summary information specific to each factor included in the overall meta-analysis.

Table S13 Summary information specific to each factor included in the confined meta-analysis (restricted to high quality studies).

Figure S1 Trends analysis in age-standardised incidence and mortality from 1990 to 2019: Data from GBD 2019.

Figure S2 Trends analysis in age-standardised incidence from 1988 to 2012: Data from GLOBOCAN 2020 online database.

Figure S3 Trends analysis in age-standardised incidence and mortality rates by geographic partition from 1972 to 2020.

Figure S4 Trends analysis in age-standardised incidence and mortality rates by economic partition from 1972 to 2020.

Table S1 Search strategy to identify studies reporting the ASIR, ASMR and risk factors for CRC in China.

**Medline**	
(1)	colorectal.mp.
(2)	rectal.mp.
(3)	rectum.mp. or Rectum/
(4)	colonic.mp.
(5)	colon.mp. or Colon, Sigmoid/ or Colon, Ascending/ or Colon/ or Colon, Transverse/ or Colon, Descending/
(6)	bowel.mp.
(7)	sigmoid.mp. or Colon, Sigmoid/
(8)	intestin.mp.
(9)	**1 or 2 or 3 or 4 or 5 or 6 or 7 or 8**
(10)	cancer.mp. or Neoplasms/
(11)	Carcinoma/ or carcinoma.mp.
(12)	Neoplasms/ or neoplas*.mp.
(13)	tumor.mp. or Neoplasms/
(14)	Adenoma/ or adenoma.mp.
(15)	lesion.mp.
(16)	CRC.mp.
(17)	**10 or 11 or 12 or 13 or 14 or 15 or 16**
(18)	exp incidence or Inciden*.mp.
(19)	exp prevalence or Prevalen*.mp.
(20)	morbidity.mp. or Morbidity/
(21)	mortality/ or mortality.mp.
(22)	epidemiology.mp. or Epidemiology/
(23)	risk factor.mp. or Risk Factors/
(24)	burden.mp. or Tumor Burden/
(25)	trend.mp.
(26)	fatality.mp.
(27)	**18 or 19 or 20 or 21 or 22 or23 or 24 or 25 or 26**
(28)	China.mp. or exp China/
(29)	Chinese.mp.
(30)	**28 or 29**
(31)	**9 and 17 and 27 and 30**
**Embase**	
(1)	colorectal.mp.
(2)	rectal.mp.
(3)	rectum.mp. or Rectum/
(4)	colonic.mp.
(5)	colon.mp. or Colon, Sigmoid/ or Colon, Ascending/ or Colon/ or Colon, Transverse/ or Colon, Descending/

(6)	bowel.mp.
(7)	sigmoid.mp. or Colon, Sigmoid/
(8)	intestin.mp.
(9)	**1 or 2 or 3 or 4 or 5 or 6 or 7 or 8**
(10)	cancer.mp. or Neoplasms/
(11)	Carcinoma/ or carcinoma.mp.
(12)	Neoplasms/ or neoplas*.mp.
(13)	tumor.mp. or Neoplasms/
(14)	Adenoma/ or adenoma.mp.
(15)	lesion.mp.
(16)	CRC.mp.
(17)	**10 or 11 or 12 or 13 or 14 or 15 or 16**
(18)	exp incidence or Inciden*.mp.
(19)	exp prevalence or Prevalen*.mp.
(20)	morbidity.mp. or Morbidity/
(21)	mortality/ or mortality.mp.
(22)	epidemiology.mp. or Epidemiology/
(23)	risk factor.mp. or Risk Factors/
(24)	burden.mp. or Tumor Burden/
(25)	trend.mp.
(26)	fatality.mp.
(27)	**8 or 19 or 20 or 21 or 22 or23 or 24 or 25 or 26**
(28)	China.mp. or exp China/
(29)	Chinese.mp.
(30)	**28 or 29**
(31)	**9 and 17and 27 and 30**

(continue)

Database	Access Date	Search Terms
**Scopus**	June 2023	(TITLE-ABS-KEY (colorectal OR rectal OR rectum OR colonic OR colon OR sigmoid OR ascending OR transverse OR descending OR bowel OR intestin)) AND (TITLE-ABS-KEY (cancer OR carcinoma OR neoplasms OR neoplas* OR tumor OR adenoma OR lesion OR CRC)) AND (TITLE-ABS-KEY (incidence OR prevalen* OR morbidity OR epidemiology OR risk factor OR burden OR tumor burden OR trend OR fatality)) AND (TITLE-ABS-KEY (China OR Chinese))
**Web of Science**	June 2023	colorectal or rectal or rectum or colonic or colon or sigmoid or ascending or transverse or descending or bowel or intestin (Topic) and cancer or carcinoma or neoplasms or neoplas* or tumor or adenoma or lesion or CRC (Topic) and incidence or prevalen* or morbidity or epidemiology or risk factor or burden or tumor burden or trend or fatality (Topic) and China OR Chinese (Topic)
**Wanfang**	June 2023	(题名:(结直肠癌) + 题名:(结直肠肿瘤) + 题名:(结直肠腺瘤) +题名:(结肠癌) + 题名:(结肠肿瘤) + 题名:(结肠腺瘤) + 题名:(直肠癌) + 题名:(直肠肿瘤) + 题名:(直肠腺瘤)+ 题名:(CRC)) and (题名:(发病率) + 题名:(发生率) + 题名:(患病率) +题名:(罹患率) + 题名:(现患率) + 题名:(死亡率) + 题名:(病死率) + 题名:(流行) + 题名:(负担) + 题名:(危险因素) + 题名:(趋势))
**CBM**	June 2023	(结直肠癌 or 结直肠肿瘤 or 结直肠腺瘤 or 结肠肿瘤 or 结肠腺瘤 or 结肠癌 or 直肠肿瘤 or 直肠腺瘤 or 直肠癌 or CRC) AND (发病率 or 发生率 or 患病率 or 罹患率 or 现患率 or 死亡率 or 病死率 or 流行 or 负担 or 危险因素 or 趋势)
**CNKI**	June 2023	(SU % '结直肠癌' + '结直肠肿瘤' + '结直肠腺瘤' + '结肠癌' + '结肠肿瘤' + '结肠腺瘤' + '直肠癌' + '直肠肿瘤' + '直肠腺瘤' + 'CRC') AND (SU % '患病率' + '发病率' + '现患率' + '罹患率' + '流行' + '发生率' + '死亡率' + '病死率' + '负担' + '危险因素' + '趋势')

Table S2 Quality assessment of the included case-control study.

Author, year	Study design	Selection				Comparability	Outcome			Final score
		Is the case definition adequate?	Representativeness of the cases	Selection of Controls	Definition of Controls		Ascertainment of exposure	Same method of ascertainment for cases and controls	Non-Response rate	
Tang, 2021	CC	1	1	1	0	0	1	1	0	5
Zhang, 2020	CC	1	1	1	0	1	1	1	0	6
Ma, 2020	CC	0	0	1	1	1	1	1	0	5
Dong, 2019	CC	1	1	1	1	0	1	1	0	6
Wu, 2018	CC	1	1	1	1	1	1	1	0	7
Chang, 2018	CC	1	1	1	0	0	1	1	0	5
Yang, 2017	CC	1	1	1	0	1	1	1	0	6
Yuan, 2016	CC	1	1	1	1	1	1	1	0	7
Shuai, 2016	CC	0	1	0	1	0	1	1	0	4
Cao, 2016	CC	1	1	1	1	1	1	1	0	7
Zhang, 2014	CC	0	0	1	0	1	1	1	0	4
Li, 2013	CC	1	0	1	0	1	1	1	0	5
Wu, 2012	CC	0	0	1	1	1	1	1	0	5
Wang, 2012	CC	1	1	1	1	1	1	1	0	7
Chen, 2012	CC	1	0	1	1	0	1	1	0	5
Xiang, 2011	CC	1	1	1	1	1	1	1	0	7
Zhang, 2010	CC	1	1	1	1	0	1	1	0	6
Chen, 2010	CC	1	1	1	0	1	1	1	0	6
Ye, 2007	CC	1	1	1	1	1	1	1	0	7
Li, 2007	CC	1	1	1	1	0	0	1	0	5
Xu, 2006	CC	1	1	1	1	0	0	1	0	5
Gong, 2006	CC	1	1	1	0	1	1	1	0	6
Chen, 2006	CC	0	0	1	0	0	1	1	1	4

Chen, 2006	CC	1	0	1	1	0	1	1	0	5
Chen, 2004	CC	1	1	1	1	0	1	1	0	6
Li, 2003	CC	0	0	1	1	1	1	1	0	5
Nie, 2002	CC	1	1	1	1	2	1	1	0	8
Zhu, 2001	CC	1	1	1	1	1	1	1	0	7
Wang, 2001	CC	1	1	1	1	1	1	1	0	7
Wang, 2001	CC	1	0	1	0	1	1	1	0	5
Chen, 2001	CC	1	1	1	1	0	1	1	0	6
Zhou, 1996	CC	1	1	1	1	1	1	1	0	7
Yang, 1996	CC	1	0	1	0	0	1	1	1	5
Lai, 1995	CC	1	1	1	1	1	1	0	0	6
Yang, 1994	CC	0	0	1	1	1	1	0	0	4
Yang, 1994	CC	0	1	1	0	1	1	1	1	6
Liu, 1994	CC	1	1	1	0	1	1	1	0	6
Zhao, 1993	CC	1	1	1	0	1	1	1	1	7
Yang, 1993	CC	0	1	1	0	1	1	1	0	5
Liu, 1993	CC	1	1	1	1	1	1	1	0	7
Zhang, 1992	CC	1	1	1	0	1	1	1	0	6
Yang, 1992	CC	0	1	1	0	1	1	1	0	5
Ding, 1992	CC	1	1	0	1	1	1	1	0	6
Wu, 1990	CC	1	1	1	0	1	1	1	0	6
Guo, 1987	CC	1	0	1	0	1	1	1	0	5
Lu, 1986	CC	1	1	1	1	1	0	0	0	5
Nie, 2018	CC	0	1	1	1	0	1	0	0	4
Gao, 2017	CC	1	1	0	1	1	1	1	0	6
Wu, 2016	CC	1	1	0	1	0	1	1	0	5
Wu, 2016	CC	1	1	0	1	1	1	1	0	6
Liu, 2015	CC	1	1	0	0	0	1	1	0	4
Luo, 2014	CC	1	1	1	1	0	1	1	0	6
Yu, 2013	CC	1	1	1	0	0	1	1	0	5
Li, 2012	CC	1	1	0	1	0	1	1	0	5

Zhang, 2011	CC	1	1	0	1	0	1	1	0	5
Yao, 2011	CC	1	1	1	1	0	1	1	0	6
Li, 2010	CC	1	1	0	1	1	1	1	0	6
Che, 2010	CC	1	0	0	1	1	1	1	0	5
Peng, 2009	CC	1	1	0	1	1	1	1	0	6
Zhao, 2008	CC	1	1	0	1	1	1	1	0	6
Feng, 2008	CC	1	1	0	1	1	1	1	0	6
Song, 2007	CC	1	1	0	1	1	1	1	0	6
Li, 2007	CC	1	1	0	1	0	1	1	0	5
Hu, 2007	CC	0	1	0	1	0	1	1	0	4
Zhang, 2005	CC	1	1	1	1	1	1	1	0	7
Ma, 2005	CC	1	1	0	1	1	1	1	0	6
Chen, 2003	CC	1	1	1	0	0	1	1	0	5
Zhang, 2002	CC	1	1	1	0	1	1	1	0	6
Xiong, 2002	CC	1	1	0	1	1	1	1	0	6
Liu, 1997	CC	1	1	0	1	1	1	1	0	6
Meng, 1994	CC	1	1	1	1	1	1	1	0	7
Jiang, 2004	CC	0	1	1	1	1	1	1	0	6
Luo, 2021	CC	1	1	1	1	1	1	1	0	7
Li, 2021	CC	1	1	1	1	1	1	1	0	7
Zhang, 2020	CC	1	1	1	1	1	1	1	1	8
Wang, 2020	CC	1	1	1	0	0	1	1	0	5
Huang, 2020	CC	1	1	1	1	1	1	1	1	8
Luo, 2019	CC	1	1	1	1	1	1	1	1	8
Luo, 2019	CC	1	1	1	1	1	1	1	0	7
Huang, 2018	CC	1	1	1	1	1	1	1	1	8
Xu, 2015	CC	0	1	1	0	1	1	1	0	5
Song, 2015	CC	1	1	1	1	0	1	1	0	6
Qin, 2015	CC	1	1	1	0	0	1	1	0	5
Luo, 2015	CC	1	1	1	1	1	1	1	0	7
Lu, 2015	CC	1	1	1	1	1	1	1	0	7

Zhong, 2014	CC	1	1	1	1	1	1	1	0	7
Takata, 2014	CC	1	1	1	1	2	1	1	0	8
Gao, 2014	CC	1	1	1	1	0	1	1	0	6
Zhong, 2013	CC	1	1	1	1	1	1	1	1	8
Hou, 2006	CC	1	1	1	0	1	1	1	0	6
Qiu, 2005	CC	1	1	0	1	1	1	1	0	6
Hou, 2004	CC	1	1	1	0	1	1	1	0	6
Ho, 2004	CC	1	1	1	1	1	1	1	0	7
Chiu, 2003	CC	1	1	1	0	0	1	1	0	5
Ji, 2002	CC	1	1	1	0	1	1	1	0	6
Ji, 1997	CC	1	1	1	0	1	1	1	0	6
Whittemore, 1990	CC	1	1	1	0	1	1	1	0	6
Xu, 1984	CC	1	1	1	0	1	1	1	0	6
Fang, 2019	CC	1	1	1	1	1	1	1	0	7
Wu, 2019	CC	1	1	1	1	1	1	1	0	7
Wang, 2018	CC	1	1	0	1	1	1	1	0	6
Huang, 2010	CC	1	1	0	1	1	1	1	1	7
Wei, 2009	CC	1	0	1	0	1	1	1	0	5
Zeng, 1993	CC	1	1	1	0	0	1	1	0	5
Ho, 2006	CC	0	1	1	0	0	1	1	0	4
Vogtmann, 2014	CC	1	1	1	0	1	1	1	0	6
Wang,2018	CC	1	1	1	1	1	1	1	0	7
Lin,2018	CC	1	1	1	1	1	1	1	1	8
Wu,2009	CC	1	1	1	1	1	1	1	0	7
Tang,1999	CC	1	1	1	1	1	1	1	0	7
Liu,2021	CC	1	1	1	1	1	1	1	1	8

[1] **Abbreviations:** CC, case-control study.

Table S3 Quality assessment of the included cohort study.

Author, year	Study design	Selection				Comparability	Outcome			Final score
		Representativeness of the exposed cohort	Selection of the non-exposed cohort	Ascertainment of exposure	Demonstration that outcome of interest was not present at start of study		Assessment of outcome	Was follow-up long enough for outcomes to occur	Adequacy of follow up of cohorts	
Wang, 2017	CO	1	1	1	1	1	1	1	1	8
He, 2006	CO	1	1	0	0	1	1	1	1	6
Chen, 2004	CO	1	1	0	1	0	1	1	0	5
Chen, 2004	CO	1	1	0	1	1	1	1	0	6
Chen, 2004	CO	1	1	0	1	1	1	1	0	6
Chen, 2004	CO	1	1	1	1	1	1	1	1	8
Chen, 1991	CO	1	1	1	0	0	1	1	0	5
Yang, 2019	CO	1	1	1	0	1	1	1	0	6
Wang, 2021	CO	1	1	1	1	0	1	1	1	7
Pang, 2021	CO	1	1	1	1	0	1	1	1	7
Liu, 2021	CO	1	1	1	1	1	1	1	0	7
Keskin, 2021	CO	1	1	1	0	1	1	1	0	6
Im, P. K., 2021	CO	1	1	1	1	1	1	1	0	7
Pang, 2020	CO	1	1	1	1	1	1	1	0	7
Li, 2020	CO	1	1	1	1	1	1	1	0	7
Yu, 2019	CO	1	1	1	1	1	1	1	0	7
Song, 2019	CO	1	1	1	0	1	1	1	0	6
Liu, 2019	CO	1	1	1	1	1	1	1	1	8
Li, X., 2019	CO	1	1	1	0	2	1	1	0	7
Zhang, 2018	CO	1	1	1	1	1	1	1	0	7
Pang, 2018	CO	1	1	1	1	1	1	1	0	7
Vogtmann, 2013	CO	1	1	1	1	1	1	1	0	7
Li, 2013	CO	1	1	1	1	1	1	1	0	7

Murphy, 2009	CO	1	1	1	1		1	1	0	6
Wu, 2013	CO	1	1	1	1	1	1	1	0	7
Nechuta, 2012	CO	1	1	1	0	1	1	1	0	6
Yang, 2011	CO	1	1	1	1	1	1	1	0	7
Li, 2011	CO	1	1	1	1	1	1	1	0	7
Yang, 2009	CO	1	1	1	1	1	1	1	0	7
Murff, 2009	CO	1	1	1	1	1	1	1	0	7
Lee, 2009	CO	1	1	1	1	0	1	1	0	6
Yang, 2007	CO	1	1	1	1	1	1	1	0	7
Shin, 2006	CO	1	1	1	1	1	1	1	0	7
Wang,2023	CO	1	1	1	1	1	1	1	0	7
Lin,2014	CO	1	1	1	1	0	1	1	1	7
Su,2022	CO	1	1	1	1	1	1	0	1	7
Kakkoura,2022	CO	1	1	1	1	1	1	0	1	7
Liu,2022	CO	1	1	1	0	1	1	0	1	6

[1]**Abbreviations:** CO**,** cohort study.

Table S4 Evidence credibility grading criteria.

**Category**	**Criteria**
Convincing evidence (class I)	● *P*-value < 1×10^{-6}<br>● More than 1000 cases<br>● 95% prediction interval excluding the null value<br>● $I^2 < 50\%$<br>● No small-study effects and excess significance bias
Highly suggestive evidence (class II)	● *P*-value < 1×10^{-6}<br>● More than 1000 cases<br>●A statistically significant result reported in the largest individual study
Suggestive evidence (class III)	● *P*-value < 1×10^{-3}<br>● More than 1000 cases
Weak evidence (class IV)	● *P*-value < 0.05
Non-significant (NS)	● *P*-value > 0.05

Table S5 Regional distribution of included studies.

Classification	Region	Covered provinces
Geographical regions	Northeast China	Heilongjiang province, Jilin province, Liaoning province
	North China	Beijing Municipality, Hebei province, Inner Mongolia Autonomous Region, Shanxi province, Tianjin Municipality
	East China	Anhui province, Fujian province, Jiangsu province, Jiangxi province, Shandong province, Shanghai Municipality, Zhejiang province
	Sourth Central China	Guangdong province, Guangxi Zhuang Autonomous Region, Hainan province, Henan province, Hubei province, Hunan province
	Northwest China	Gansu province, Ningxia Hui Autonomous Region, Qinghai province, Shaanxi province, Xinjiang Uyghur Autonomous Region
	Southwest China	Chongqing Municipality, Guizhou province, Sichuan province, Tibet Autonomous Region, Yunnan province
Economic regions	Northeast region	Heilongjiang province, Jilin province, Liaoning province
	Central region	Henan province, Hubei province, Hunan province, Anhui province, Shanxi province, Jiangxi province
	East region	Beijing Municipality, Tianjin Municipality, Hebei province, Shandong province, Shanghai Municipality, Jiangsu province, Zhejiang province, Fujian province, Guangdong province, Hainan province
	West region	Chongqing Municipality, Guizhou province, Sichuan province, Tibet Autonomous Region, Yunnan province, Guangxi Zhuang Autonomous Region, Xinjiang Uyghur Autonomous Region, Ningxia Hui Autonomous Region, Qinghai province, Shaanxi province, Gansu province, Inner Mongolia Autonomous Region

Table S6 The number of the included studies among different regions.

Subtypes	Subregion	Number of studies (ASIR)	Number of studies (ASMR)
Geographical Partitions	Northeast China	13	16
	North China	20	18
	East China	70	79
	Sourth Central China	50	51
	Northwest China	14	11
	Sourthwest China	24	25
Economic Partitions	Northeast China	13	16
	East China	91	98
	Central China	33	36
	West China	54	50

Table S7 Characteristics of the included studies.

Author (year)	study type	Cases(n) / Total(n)	Outcomes	Lifestyle factors	Dietary factors	Diseases history factors	Mental profile factors	Sociodemographic factors	Anthropometrics factors
					**Case-control study**				
Tang, 2021	CC	316 / 639	CRC	Exercise, Smoking, Alcohol, DII	None	None	None	None	None
Zhang, 2020	CC	356 / 712	CRC	Tea, Sedentary lifestyle, Exercise	None	Family history of cancer	None	Married	None
Ma, 2020	CC	105 / 315	CRC	Sedentary lifestyle, Smoking, Tea, High fat and greasy diet	Fried food, Smoked products, Salted products	Family history of cancer, Diabetes, Enteritis, Haemorrhoids, Colon polyps	History of mental stimulation	None	None
Dong, 2019	CC	135 / 2060	CRC	None	None	Helicobacter pylori infection	None	None	None
Wu, 2018	CC	763 / 1526	CRC	Tea	None	None	None	None	None
Chang, 2018	CC	300 / 600	CRC	Smoking, Alcohol, High fat and greasy diet	Smoked products, Fiber	None	None	None	None
Yang, 2017	CC	301 / 600	CRC	Tea, Alcohol, Smoking, Sedentary lifestyle	None	Family history of CRC	None	None	None
Yuan, 2016	CC	200 / 400	CRC	Sedentary lifestyle	Fiber, Red meat	None	None	None	None
Shuai, 2016	CC	251 / 509	CRC	Alcohol, Smoking	None	None	None	None	None
Cao, 2016	CC	218 / 436	CRC	Salted food	None	None	None	None	None
Zhang, 2014	CC	121 / 1275	CRC	Alcohol	Smoked products	History of gastrointestinal diseases, Family history of cancer	Type A personality, Bad mental state	None	None
Li, 2013	CC	456 / 912	CRC	High fat and greasy diet, Alcohol	None	None	None	None	None
Wu, 2012	CC	560 / 1703	CRC	None	None	None	Negative emotion, History of mental stimulation	None	None

Wang, 2012	CC	102 / 306	CRC	Exercise	None	History of gastrointestinal diseases, Family history of cancer	Emotional regulation	None	None
Chen, 2012	CC	302 / 613	Rectal cancer	Smoking	None	None	None	None	None
Xiang, 2011	CC	342 / 735	CRC	None	Calcium, Iron, Znic, Selenium	None	None	None	None
Zhang, 2010	CC	215 / 430	CRC	Irregular bowel movement, Alcohol	None	Mucous bloody stool	None	None	None
Chen, 2010	CC	125 / 250	Colon cancer	High fat and greasy diet	Roast food	None	None	None	None
Ye, 2007	CC	136 / 272	CRC	High fat and greasy diet	Aquatic product	Enteritis	Emotional regulation	None	None
Li, 2007	CC	1869 / 5607	CRC	None	None	Mucous bloody stool, Colon polyps, Family history of CRC	History of mental stimulation	None	None
Xu, 2006	CC	150 / 450	CRC	None	Total energy, Fiber, Cholesterol	Mucous bloody stool, Colon polyps	None	None	None
Gong, 2006	CC	210 / 439	Rectal cancer	Smoking, Alcohol	None	None	None	None	None
Chen, 2006	CC	140 / 483	CRC	None	Folate	None	None	None	None
Chen, 2006	CC	140 / 483	Colon cancer & Rectal cancer	Smoking	Red meat	Mucous bloody stool, Colon polyps	None	None	None
Chen, 2004	CC	140 / 483	Colon cancer & Rectal cancer	None	Fried food, Smoked products, Salted food	None	None	None	None
Li, 2003	CC	150 / 300	Colon cancer	Smoking	Milk and dairy products, Vegetables, Fiber, Beans and soy products	None	Type A personality	None	None
Nie, 2002	CC	100 / 300	CRC	None	None	Appendiceal Diseases, Family history of cancer	Emotional regulation	None	None
Zhu, 2001	CC	308 / 924	CRC	Alcohol, Animal oil	Eggs, Red meat	None	None	None	None
Wang, 2001	CC	109 / 872	Colon cancer	Animal oil	Vegetables, Red meat, Smoked products	History of gastrointestinal diseases, Family history of CRC	Negative emotion, Bad mental state	None	None
Wang, 2001	CC	151 / 1691	Colon cancer	Alcohol	Vegetables	History of gastrointestinal	Negative emotion	Static occupation	None

						disease, History of schistosomiasis, Family history of CRC			
Chen, 2001	CC	196 / 1176	Colon cancer & Rectal cancer	None	None	Mucous bloody stool, Colon polyps	None	None	None
Zhou, 1996	CC	245 / 490	CRC	Alcohol	None	Mucous bloody stool, Chronic diarrhea	None	None	None
Yang, 1996	CC	3166 / 6169	CRC	None	None	Colon polyps, Enteritis, History of hepatobiliary disease, Diabetes, History of schistosomiasis	None	None	None
Lai, 1995	CC	129 / 258	CRC	Sedentary lifestyle	Fiber, SFA	None	None	None	None
Yang, 1994	CC	735 / 2186	Colon cancer	Smoking, Tea	None	Colon polyps, Family history of CRC	None	None	None
Yang, 1994	CC	1301 / 2701	Colon cancer & Rectal cancer	None	Calcium, Fiber	None	None	None	None
Liu, 1994	CC	286 / 572	CRC	Alcohol	None	Mucous bloody stool, Chronic diarrhea	None	None	None
Zhao, 1993	CC	202 / 404	Colon cancer & Rectal cancer	None	None	History of gastrointestinal diseases, Haemorrhoids	None	None	None
Yang, 1993	CC	1328 / 2779	Colon cancer & Rectal cancer	None	Calcium, Iron, Znic, Selenium	None	None	None	None
Liu, 1993	CC	110 / 220	Rectal cancer	Animal oil, High fat and greasy diet	None	Family history of cancer	None	None	None
Zhang, 1992	CC	250 / 750	CRC	Moderate physical activity, Heavy physical activity	Vegetables, All meat	Family history of cancer	Bad mental state	None	None
Yang, 1992	CC	850 / 1707	Colon cancer & Rectal cancer	None	Vitamin C, Carbohydrate, Protein, Fiber, Total fat, Calcium	Family history of CRC	None	None	None

Ding, 1992	CC	210 / 420	CRC	None	Fried food	Mucous bloody stool, Appendiceal Diseases, Family history of cancer	None	None	None
Wu, 1990	CC	114 / 228	CRC	None	None	Appendiceal Diseases, Colon polyps, Enteritis, History of hepatobiliary disease, History of schistosomiasis, Haemorrhoids	None	None	None
Guo, 1987	CC	197 / 397	CRC	Smoking	None	Family history of CRC	None	None	None
Lu, 1986	CC	202 / 606	Colon cancer	None	Protein, Total energy, Total fat, Vitamin A, Vitamin C, Fiber, Cholesterol, Aquatic product, Beans and soy products	Family history of CRC, Haemorrhoids, Enteritis	Type A personality	None	None
Nie, 2018	CC	400 / 800	CRC	None	None	Family history of CRC, Diabetes	None	None	None
Gao, 2017	CC	104 / 312	CRC	Smoking, High fat and greasy diet	None	Family history of cancer	None	None	None
Wu, 2016	CC	1953 / 6107	CRC	DII	None	None	None	None	None
Wu, 2016	CC	110 / 220	CRC	Animal oil	Fried food, Salted food	None	None	None	None
Liu, 2015	CC	299 / 607	Rectal cancer	Smoking	None	None	None	None	None
Luo, 2014	CC	844 / 2119	CRC	None	None	Diabetes	None	None	None
Yu, 2013	CC	577 / 1154	CRC	None	None	Colon polyps, Family history of CRC, Diabetes	None	None	None
Li, 2012	CC	256 / 744	CRC	None	None	None	Negative emotion	None	None
Zhang, 2011	CC	256 / 628	CRC	Smoking, Alcohol	None	Diabetes	None	None	BMI
Yao, 2011	CC	202 / 404	CRC	None	None	Diabetes, History of hepatobiliary disease	None	None	None
Li, 2010	CC	111 / 222	Rectal cancer	None	None	Haemorrhoids, Family history of cancer	None	None	None
Che, 2010	CC	110 / 220	CRC	High fat and greasy diet	Milk and dairy products, Fruits, Aquatic product, Fiber, Salted food	None	None	None	None

Peng, 2009	CC	286 / 572	CRC	Smoking	Fiber	History of gastrointestinal diseases, Family history of cancer	None	None	None
Zhao, 2008	CC	142 / 284	Colon cancer	Sedentary lifestyle, Animal oil	Vegetables	Appendiceal Diseases, History of hepatobiliary disease, Family history of cancer	None	None	None
Feng, 2008	CC	118 / 236	Rectal cancer	Sedentary lifestyle, High fat and greasy diet, Exercise	Vegetables	Family history of cancer	None	None	None
Song, 2007	CC	172 / 344	CRC	None	Aquatic product	Family history of cancer	None	Static occupation	None
Li, 2007	CC	660 / 5233	CRC	None	None	Diabetes, Family history of CRC	None	None	None
Hu, 2007	CC	364 / 1097	CRC	Smoking, Alcohol	None	Diabetes, Family history of CRC, Hypertension	None	None	Triglyceride
Zhang, 2005	CC	113 / 370	CRC	Irregular bowel movement	Fruits	Mucous bloody stool	None	None	None
Ma, 2005	CC	478 / 1316	CRC	Moderate physical activity, Heavy physical activity, Exercise	None	None	None	None	BMI
Chen, 2003	CC	448 / 1079	CRC	None	None	History of hepatobiliary disease	None	None	None
Zhang, 2002	CC	269 / 538	CRC	None	Vegetables, Eggs, Aquatic product	History of gastrointestinal diseases	None	None	None
Xiong, 2002	CC	132 / 264	CRC	None	Calcium, Fried food, Salted food	Family history of cancer	None	None	None
Liu, 1997	CC	153 / 306	CRC	Animal oil	None	Enteritis	None	Static occupation	None
Meng, 1994	CC	100 / 200	CRC	None	Aquatic product, Salted food, Red meat	Family history of cancer	None	None	None
Jiang, 2004	CC	126 / 469	Colon cancer & Rectal cancer	Smoking, Animal oil, Well water	None	Mucous bloody stool, Colon polyps, History of hepatobiliary disease, Family history of CRC	None	None	None
Luo, 2021	CC	493 / 991	CRC	None	Znic, Selenium	None	None	None	None

Li, 2021	CC	2502 / 5040	CRC	None	None	None	None	Education	None
Zhang, 2020	CC	2380 / 4769	CRC	None	Milk and dairy products, Calcium	None	None	None	None
Wang, 2020	CC	304 / 2666	CRC	None	None	Helicobacter pylori infection	None	None	None
Huang, 2020	CC	2502 / 5040	CRC	None	Folate, Vitamin B	None	None	None	None
Luo, 2019	CC	2138 / 4282	CRC	None	Iron	None	None	None	None
Luo, 2019	CC	535 / 1087	CRC	None	Vitamin A, Vitamin E	None	None	None	None
Huang, 2018	CC	1944 / 3971	CRC	None	Carbohydrate, Fiber	None	None	None	None
Xu, 2015	CC	1189 / 2378	CRC	None	Aquatic product, Salted food	None	None	None	None
Song, 2015	CC	265 / 517	CRC	None	Fiber	None	None	None	None
Qin, 2015	CC	466 / 2796	CRC	None	Vegetables, Salted food	None	None	None	None
Luo, 2015	CC	1057 / 2114	CRC	None	Fruit and vegetables	None	None	None	None
Lu, 2015	CC	845 / 1690	CRC	None	Carotenoid	None	None	None	None
Zhong, 2014	CC	613 / 1226	CRC	None	Fiber	None	None	None	None
Takata, 2014	CC	288 / 863	CRC	None	Folate	None	None	None	None
Gao, 2014	CC	315 / 754	CRC	Alcohol	None	None	None	None	None
Zhong, 2013	CC	489 / 1465	CRC	None	SFA, UFA	None	None	None	None
Hou, 2006	CC	931 / 2483	Colon cancer	None	None	None	None	None	BMI
Qiu, 2005	CC	142 / 427	Colon cancer	None	None	History of schistosomiasis	None	None	None
Hou, 2004	CC	931 / 2483	Colon cancer	Moderate physical activity, Heavy physical activity	None	None	None	None	None
Ho, 2004	CC	822 / 1748	CRC	Smoking, Alcohol	None	None	None	None	None
Chiu, 2003	CC	931 / 2483	Colon cancer	None	Milk and dairy products, Fruit and vegetables, Eggs, All meat, Aquatic product, Beans and soy products, Salted food, Total energy, Carotenoid,	None	None	None	None

					Vitamin A, Vitamin E, Carbohydrate, Protein, Fiber, Total fat				
Ji, 2002	CC	1805 / 3357	Colon cancer & Rectal cancer	Smoking, Alcohol	None	None	None	None	None
Ji, 1997	CC	1805 / 3357	Colon cancer & Rectal cancer	Tea	None	None	None	None	None
Whittemor, 1990	CC	432 / 1728	CRC	Sedentary lifestyle	SFA, UFA, Protein	None	None	Static occupation	None
Xu, 1984	CC	252 / 504	CRC	None	None	History of schistosomiasis	None	None	None
Fang, 2019	CC	833 / 1666	CRC	None	Vegetables	None	None	None	None
Wu, 2019	CC	833 / 1666	CRC	None	Vegetables	None	None	None	None
Wang, 2018	CC	310 / 930	CRC	Alcohol	None	None	None	None	None
Huang, 2010	CC	304 / 2994	CRC	Smoking, Alcohol	None	Diabetes	None	None	None
Wei, 2009	CC	706 / 1429	CRC	Smoking, Alcohol	None	Family history of cancer	None	None	BMI
Zeng, 1993	CC	503 / 2691	CRC	None	None	History of hepatobiliary disease	None	None	None
Ho, 2006	CC	822 / 1748	CRC	Tea	Roast food	None	None	None	None
Vogtmann**,** 2014	CC	340 / 1013	CRC	None	Vegetables	None	None	None	None
Wang, 2018	CC	317 / 634	CRC	Exercise	All meat, Vegetables, Roast food, Fried food	None	Negative emotion	None	BMI
Lin, 2018	CC	166 / 332	CRC	Smoking, Alcohol	None	None	None	None	None
Wu, 2009	CC	166 / 332	CRC	Smoking, Alcohol	None	None	None	None	None
Tang, 1999	CC	163 / 326	CRC	Physical activity	None	None	None	None	None
Liu, 2021	CC	163 / 326	CRC	Physical activity	Vegetables, Fiber, Vitamin A, Vitamin E, Vitamin C, Vitamin B, Carotenoid, Calcium, Folate	None	None	None	None
**Cohort study**									

Wang, 2017	CO	336 / 73869	CRC	None	None	None	None	None	C-reactive protein
He, 2006	CO	328 / 64460	CRC	None	None	None	History of mental stimulation	Education	None
Chen, 2004	CO	242 / 64115	CRC	Smoking, Well water	None	None	None	Married, Static occupation	None
Chen, 2004	CO	242 / 64154	Colon cancer & Rectal cancer	None	None	Appendiceal Diseases	None	None	None
Chen, 2004	CO	187 / 64650	CRC	None	None	Colon polyps	None	None	None
Chen, 2004	CO	242 / 64102	CRC	Alcohol	None	None	None	Married, Static occupation	None
Chen, 1991	CO	225 / 2773985	Colon cancer & Rectal cancer	Well water	None	None	None	None	None
Yang, 2019	CO	3056 / 510134	CRC	Irregular bowel movement	None	None	None	None	None
Wang, 2021	CO	255 / 54187	CRC	Smoking, Alcohol, Exercise	None	History of hepatobiliary disease, Hypertension, Diabetes	None	Education	BMI, Triglyceride, C-reactive protein
Pang, 2021	CO	3061 / 510137	CRC	None	None	History of hepatobiliary disease	None	None	None
Liu, 2021	CO	448 / 93390	CRC	None	None	History of hepatobiliary disease	None	None	None
Keskin, 2021	CO	179 / 29553	CRC	Smoking, Alcohol	Fruit and vegetables, Eggs, All meat, Salted food	Family history of cancer	None	None	BM
Im, P. K., 2021	CO	3056 / 510137	CRC	Alcohol	None	None	None	None	None
Pang, 2020	CO	3061 / 510131	CRC	None	None	None	None	Education	None
Li, 2020	CO	144 / 23415	CRC	Smoking	None	None	None	None	None
Yu, 2019	CO	2267 / 455918	CRC	Tea	None	None	None	None	None
Song, 2019	CO	2138 / 496732	CRC	None	None	History of hepatobiliary disease	None	None	None

Liu, 2019	CO	214 / 19680	CRC	None	None	Helicobacter pylori infection	None	None	None
Li, X., 2019	CO	394 / 104333	CRC	None	None	Hypertension	None	None	Triglyceride
Zhang, 2018	CO	671 / 59503	CRC	Exercise	None	None	None	None	Waist hip rate
Pang, 2018	CO	3024 / 510136	CRC	None	None	Diabetes	None	None	None
Vogtmann, 2013	CO	398 / 61274	CRC	None	Fruit and vegetables, Beans and soy products	None	None	None	None
Li, 2013	CO	935 / 134255	CRC	None	None	None	None	None	Waist hip rate
Murphy, 2009	CO	391 / 773358	CRC	None	None	Family history of cancer, Family history of CRC	None	None	None
Wu, 2013	CO	288 / 61482	CRC	None	None	None	None	None	C-reactive protein
Nechuta, 2012	CO	1255 / 69310	CRC	Tea	None	None	None	None	None
Yang, 2011	CO	243 / 60567	CRC	Tea	None	None	None	None	None
Li, 2011	CO	475 / 73061	CRC	None	Carbohydrate	None	None	None	None
Yang, 2009	CO	321 / 68412	CRC	None	Beans and soy products	None	None	None	None
Murff, 2009	CO	3966 / 73243	CRC	None	SFA, Aquatic product	None	None	None	None
Lee, 2009	CO	394 / 73224	CRC	Tea, Exercise	Fruit and vegetables, Total energy	Family history of CRC	None	Married, Education	BMI, Waist hip rate
Yang, 2007	CO	256 / 69710	CRC	Tea	None	None	None	None	None
Shin, 2006	CO	283 / 73314	CRC	None	Calcium, Carotenoid, Vitamin A, Vitamin E, Fiber, Vitamin B	None	None	None	None
Wang, 2023	CO	630 / 28359	CRC	None	None	None	None	None	Waist hip rate
Lin, 2014	CO	446 / 2393	CRC	None	None	History of hepatobiliary disease	None	None	Triglyceride
Su, 2022	CO	458 / 52938	CRC	Exercise	None	None	None	None	None
Kakkoura, 2022	CO	3350 / 510146	CRC	None	Milk and dairy products	None	None	None	None
Liu, 2022	CO	665 / 93402	CRC	None	None	History of hepatobiliary disease	None	None	None

Table S8 Results of subgroup analysis of risk factors for CRC by site.

Risk factor	Colon cancer					Rectal cancer					P value (Heterogeneity)
	No. of studies	Case / Total	OR (95%CI)	P value	I^2(%)	No. of studies	Case / Total	OR (95%CI)	P value	I^2(%)	
**Sociodemographic**											
Education	3	3,561 / 588,395	0.85 (0.65, 1.10)	0.209	89.2	3	2,765 / 588,395	0.77 (0.59, 1.01)	0.056	86.4	0.607
Static occupation	4	538 / 131,636	1.61 (1.26, 2.06)	1.00E-04	1.8	2	394 / 65,843	1.06 (0.61, 1.82)	0.846	45.0	0.172
**Anthropometrics**											
BMI	6	>2,263 / 79,275	1.50 (1.16, 1.94)	0.002	66.0	5	>911 / 77,242	1.39 (1.18, 1.65)	1.00E-04	0.0	0.627
Waist hip rate	3	>798 / 235,838	1.31 (0.98, 1.75)	0.072	63.4	3	>531 / 235,838	1.21 (0.98, 1.50)	0.071	0.0	0.665
**Lifestyle**											
Smoking	9	>2,824 / 75,319	0.96 (0.78, 1.18)	0.680	60.3	9	>3,058 / 12,304	1.13 (0.93, 1.38)	0.212	70.6	0.264
Alcohol	8	>3,851 / 583,857	1.37 (1.06, 1.77)	0.018	88.3	8	>3,973 / 582,815	1.34 (1.04, 1.73)	0.022	88.2	0.904
Tea	7	3,187 / 212,511	0.75 (0.63, 0.90)	0.002	61.6	6	2,337 / 210,763	0.67 (0.54, 0.84)	4.00E-04	62.8	0.436
Sedentary lifestyle	3	365 / 2,270	1.42 (1.08, 1.88)	0.013	33.5	2	377 / 1,964	0.98 (0.58, 1.65)	0.943	63.6	0.219
Exercise	5	>1,036 / 135,316	0.75 (0.41, 1.38)	0.362	90.1	6	>945 / 135,552	0.63 (0.35, 1.12)	0.116	90.3	0.684
**Diet intake**											
Milk and dairy products	4	2,793 / 80,776	0.91 (0.61, 1.36)	0.651	89.9	2	986 / 77,993	0.56 (0.43, 0.73)	1.85E-05	14.2	0.048
Fruit and vegetables	10	3,047 / 171,981	0.60 (0.42, 0.85)	0.004	82.9	7	1,382 / 166,807	0.67 (0.44, 1.03)	0.065	81.2	0.695
Vegetables	9	2,811 / 98,757	0.51 (0.34, 0.77)	0.001	84.2	6	1,460 / 93,583	0.60 (0.34, 1.06)	0.080	86.0	0.649
Fruits	3	1,230 / 93,310	0.84 (0.54, 1.31)	0.442	70.0	2	278 / 90,872	0.81 (0.41, 1.61)	0.543	73.7	0.930
Calcium	6	>3,569 / 86,005	1.00 (1.00, 1.00)	0.006	73.2	3	>2,096 / 84,298	0.80 (0.60, 1.08)	0.144	84.0	0.146
Iron	3	>2,029 / 7,796	1.09 (0.86, 1.38)	0.466	55.5	3	>1,404 / 7,796	1.00 (0.87, 1.15)	0.994	0.0	0.538
Znic	2	>726 / 3,514	0.68 (0.32, 1.46)	0.323	55.5	2	1,670 / 3,514	0.62 (0.18, 2.09)	0.439	92.8	0.900
Selenium	2	>726 / 3,514	1.05 (0.77, 1.44)	0.741	36.0	2	1,670 / 3,514	0.97 (0.86, 1.09)	0.630	0.0	0.643
Eggs	3	1,230 / 105,260	1.44 (1.18, 1.76)	4.00E-04	0.0	2	274 / 102,777	1.43 (1.03, 2.00)	0.034	0.0	0.972
Meat	6	1,611 / 107,249	1.35 (1.12, 1.62)	0.001	38.5	4	459 / 103,894	1.12 (0.80, 1.57)	0.505	11.3	0.341
Red meat	5	1,548 / 77,696	1.39 (1.15, 1.68)	7.00E-04	27.0	3	343 / 74,341	1.02 (0.59, 1.78)	0.932	48.9	0.299
Vitamin A	4	1,596 / 77,490	0.98 (0.68, 1.42)	0.919	74.9	2	348 / 74,401	0.66 (0.21, 2.04)	0.472	84.6	0.517
Vitamin E	3	1,394 / 76,884	0.69 (0.49, 0.99)	0.042	61.0	2	281 / 75,021	0.53 (0.33, 0.84)	0.008	0.0	0.377
Vitamin C	4	1,774 / 78,110	0.67 (0.44, 1.00)	0.052	88.3	2	349 / 74,401	0.87 (0.53, 1.43)	0.589	30.1	0.427
Carbohydrate	4	2,866 / 81,222	0.95 (0.70, 1.29)	0.759	52.4	2	960 / 77,032	0.88 (0.71, 1.10)	0.262	0.0	0.690
Fiber	11	4,491 / 88,811	0.72 (0.62, 0.85)	5.72E-05	85.6	7	2,327 / 83,694	0.71 (0.59, 0.86)	4.00E-04	84.5	0.911
Aquatic product	5	2,290 / 151,934	1.01 (0.80, 1.28)	0.908	78.9	3	758 / 148,845	0.75 (0.50, 1.11)	0.151	83.0	0.208

SFA	3	676 / 76,417	1.21 (1.04, 1.40)	0.012	0.0	4	714 / 76,675	1.12 (0.99, 1.25)	0.063	64.1	0.423
UFA	4	876 / 149,660	1.12 (0.93, 1.36)	0.239	0.0	4	767 / 149,660	0.86 (0.70, 1.06)	0.153	25.7	0.066
Beans and soy products	5	1,714 / 133,075	0.88 (0.75, 1.03)	0.103	37.5	2	288 / 129,686	0.61 (0.43, 0.87)	0.006	0.0	0.063
Smoked products	3	402 / 74,579	1.18 (0.96, 1.43)	0.110	72.2	2	241 / 73,707	0.96 (0.75, 1.23)	0.768	27.3	0.203
Salted food	5	2,008 / 108,121	1.43 (1.02, 2.02)	0.040	85.7	6	825 / 105,638	1.04 (0.89, 1.22)	0.615	0.0	0.097
Well water	2	>107 / 2,838,100	0.73 (0.13, 3.95)	0.713	95.7	3	362 / 2,838,569	0.58 (0.14, 2.34)	0.440	96.7	0.895
**Personal history**											
History of hepatobiliary disease	5	3,860 / 519,750	1.96 (1.11, 3.46)	0.021	78.5	3	3,509 / 518,997	1.02 (0.81, 1.29)	0.855	55.3	0.037
Family history of cancer	9	2,030 / 110,907	2.08 (1.36, 3.18)	7.00E-04	61.0	7	1,697 / 107,663	1.68 (1.23, 2.29)	1.00E-03	61.7	0.426
Family history of CRC	6	1,477 / 79,641	2.30 (1.45, 3.65)	4.00E-04	36.8	2	884 / 76,003	1.65 (1.00, 2.71)	0.048	0.0	0.338
Diabetes	2	3,411 / 516,305	1.16 (1.00, 1.35)	0.045	0.0	2	4,882 / 516,305	1.17 (0.96, 1.42)	0.114	0.0	0.946

[1]**Abbreviations:** CRC, colorectal cancer; BMI, body mass index; UFA, unsaturated fatty acids; SFA, saturated fatty acids; OR, odds ratio; CI, confidence interval.

Table S9 Results of subgroup analysis of risk factors for CRC by sex.

Risk factor	Colorectal cancer (male)				Colorectal cancer (female)				P value (Heterogeneity)
	No. of studies	OR (95%CI)	P value	I^2(%)	No. of studies	OR (95%CI)	P value	I^2(%)	
**Sociodemographic**									
Education	2	0.56 (0.27, 1.17)	0.125	88.8	2	0.68 (0.49, 0.94)	0.019	75.0	0.635
**Anthropometrics**									
BMI	3	1.46 (1.15, 1.86)	0.002	0.0	3	1.25 (0.99, 1.58)	0.063	0.0	0.364
Waist hip rate	2	1.36 (1.10, 1.69)	0.004	30.4	2	1.09 (0.90, 1.32)	0.380	0.0	0.132
**Lifestyle**									
Smoking	5	0.94 (0.81, 1.09)	0.386	31.3	3	0.94 (0.67, 1.32)	0.724	47.7	1.000
Alcohol	6	1.18 (0.99, 1.39)	0.063	75.1	5	1.03 (0.90, 1.18)	0.653	0.0	0.220
Tea	4	0.80 (0.60, 1.05)	0.103	80.5	6	0.72 (0.58, 0.90)	0.003	78.7	0.562
**Diet intake**									
Milk and dairy products	2	0.66 (0.36, 1.19)	0.164	90.9	3	0.74 (0.52, 1.07)	0.112	77.7	0.748
Fruit and vegetables	7	0.84 (0.59, 1.18)	0.315	77.8	6	0.75 (0.47, 1.18)	0.209	82.6	0.700
Vegetables	7	0.82 (0.56, 1.20)	0.301	78.3	5	0.65 (0.31, 1.35)	0.248	86.2	0.583
Fruits	3	0.93 (0.49, 1.78)	0.834	80.5	2	0.78 (0.45, 1.35)	0.380	67.6	0.684
Eggs	2	1.54 (1.09, 2.16)	0.014	13.8	3	1.36 (1.10, 1.68)	0.004	0.0	0.545
All meat	2	1.51 (1.19, 1.92)	6.00E-04	0.0	3	1.07 (0.78, 1.45)	0.682	52.3	0.085
Carotenoid	2	0.45 (0.35, 0.58)	6.53E-10	0.0	3	0.76 (0.56, 1.04)	0.091	50.4	0.010
Vitamin A	2	0.82 (0.27, 2.50)	0.732	89.3	3	0.89 (0.50, 1.58)	0.690	76.5	0.898
Vitamin E	2	0.51 (0.38, 0.68)	7.56E-06	0.0	3	0.77 (0.55, 1.09)	0.141	25.7	0.072
Vitamin C	2	0.40 (0.29, 0.55)	1.97E-08	0.0	3	0.77 (0.62, 0.96)	0.019	0.0	9.00E-04
Fiber	5	0.57 (0.40, 0.80)	1.20E-03	87.6	6	0.70 (0.59, 0.83)	6.37E-05	0.0	0.297
Aquatic product	2	0.84 (0.50, 1.40)	0.505	90.9	4	0.83 (0.61, 1.13)	0.236	82.6	0.969
SFA	3	1.05 (1.00, 1.11)	0.045	0.0	3	1.30 (0.99, 1.71)	0.055	0.0	0.313
UFA	2	0.99 (0.82, 1.20)	0.931	0.0	4	0.96 (0.80, 1.14)	0.619	5.3	0.817
Beans and soy products	2	0.85 (0.66, 1.09)	0.208	0.0	2	0.72 (0.58, 0.91)	0.006	0.0	0.335
Salted food	3	1.03 (0.39, 2.73)	0.954	83.3	4	1.47 (0.92, 2.35)	0.110	84.8	0.519
**Personal history**									
History of hepatobiliary disease	4	1.88 (1.00, 3.51)	0.089	36.9	3	1.73 (1.15, 2.59)	0.008	0.0	0.827

[1]**Abbreviations:** CRC, colorectal cancer; BMI, body mass index; UFA, unsaturated fatty acids; SFA, saturated fatty acids; OR, odds ratio; CI, confidence interval.

Table S10 Results of Meta-analysis of CRC Risk Factors (restricted to cohort studies).

Risk factor	No. of studies	Case / Total	OR	95%CI	95%PI	P value	I^2(%)	Evidence grade
**Sociodemographic**								
Married	3	878 /201,441	0.87	(0.37, 2.00)	(0.18, 4.12)	0.731	83.1	NS
Education	3	3,710 /637,542	1.03	(0.95, 1.11)	(0.95, 1.12)	0.438	0.8	NS
Static occupation	2	484 /128,217	1.46	(1.14, 1.85)	(1.14, 1.85)	0.002	0.0	Weak evidence
**Anthropometrics**								
BMI	3	828 /156,964	1.25	(1.01, 1.55)	(1.01, 1.55)	0.039	0.0	Weak evidence
Waist hip rate	4	2,630 /295,341	1.27	(1.12, 1.45)	(1.03, 1.57)	2.38E-04	33.7	Suggestive evidence
Triglyceride	3	1,095 /160,835	1.08	(1.01, 1.16)	(0.99, 1.18)	0.029	23.9	Weak evidence
C-reactive protein	3	879 /189,538	1.33	(0.91, 1.93)	(0.66, 2.66)	0.141	85.2	NS
**Lifestyle**								
Smoking	4	820 /171,270	1.10	(0.72, 1.71)	(0.47, 2.59)	0.654	73.5	NS
Alcohol	4	2,203 /657,979	1.11	(1.00, 1.24)	(0.86, 1.44)	0.048	69.3	Weak evidence
Tea	5	4,415 /728,729	0.85	(0.70, 1.03)	(0.57, 1.28)	0.106	71.2	NS
Exercise	3	1,523 /185,665	0.91	(0.75, 1.11)	(0.67, 1.25)	0.375	49.1	NS
**Diet intake**								
Milk and dairy products	2	3,744 /583,370	1.01	(0.78, 1.31)	(0.68, 1.49)	0.943	46.2	NS
Fruit and vegetables	3	971 /164,051	1.05	(0.78, 1.40)	(0.61, 1.79)	0.759	60.4	NS
Vegetables	2	577 /90,827	1.00	(0.76, 1.33)	(0.76, 1.33)	0.984	0.0	NS
Fruits	2	577 /90,827	0.95	(0.48, 1.91)	(0.30, 3.01)	0.890	87.2	NS
Eggs	2	573 /102,777	1.48	(1.17, 1.89)	(1.17, 1.89)	0.001	0.0	Weak evidence
All meat	2	573 /12,777	1.14	(0.69, 1.90)	(0.52, 2.50)	0.603	69.3	NS
Aquatic product	2	790 /146,467	1.24	(1.00, 1.54)	(1.00, 1.54)	0.050	0.0	Weak evidence
UFA	2	790 /146,467	0.93	(0.72, 1.20)	(0.72, 1.20)	0.554	0.0	NS
Beans and soy products	2	719 /129,686	0.74	(0.59, 0.92)	(0.59, 0.92)	0.007	0.0	Weak evidence
Salted food	2	573 /102,777	0.91	(0.68, 1.21)	(0.68, 1.21)	0.502	0.0	NS
Well water	2	467 /2,838,100	0.54	(0.14, 2.11)	(0.04, 8.02)	0.375	97.6	NS
**Personal history**								
History of hepatobiliary disease	6	7,013 /1,250,240	1.43	(1.18, 1.74)	(0.95, 2.16)	2.64E-04	64.8	Suggestive evidence
Family history of cancer	3	964 /176,135	1.07	(0.90, 1.27)	(0.90, 1.27)	0.454	0.0	NS
Family history of CRC	2	785 /146,582	1.55	(0.91, 2.63)	(0.78, 3.04)	0.109	30.8	NS
Diabetes	2	3,279 /564,323	1.13	(1.01, 1.27)	(1.01, 1.27)	0.037	0.0	Weak evidence

Hypertension	2	649 /158,520	0.99	(0.83, 1.18)	(0.83, 1.18)	0.881	0.0	NS

[1]**Abbreviations:** CRC, colorectal cancer; BMI, body mass index; UFA, unsaturated fatty acid; OR, odds ratio; CI, confidence interval; NS, non-significant.

Table S11 Full-list of the included studies on the risk factors for CRC in China.

Risk factor	No. of studies	Study
**Sociodemographic**		
Married	4	● Lee, S.A., et al., Animal origin foods and colorectal cancer risk: a report from the Shanghai Women's Health Study. Nutrition and Cancer, 2009. 61(2): p. 194-205.<br>● Zhang, T., et al., Analysis of the related factors between lifestyle and colorectal cancer in Nanyang area(南阳地区居民生活方式与结直肠癌相关因素分析). Journal of Community Medicine, 2020. 18(18): p. 1255-1258.<br>● Chen, K., et al., Alcohol drinking and colorectal cancer:a population-based prospective cohort study(应用队列研究方法检验饮酒与肠癌发病的联系). Journal of ZheJiang University (Medical Sciences), 2004(05): p. 40-44.<br>● Chen, K., et al., Association of Drinking Water Source and Colorectal Cancer Incidence: A Prospect Cohort Study(饮水类型与结直肠癌发病率关系的前瞻性队列研究). Chinese Journal of Cancer, 2004(05): p. 550-554.
Education	4	● Lee, S.A., et al., Animal origin foods and colorectal cancer risk: a report from the Shanghai Women's Health Study. Nutrition and Cancer, 2009. 61(2): p. 194-205.<br>● Wang, Z., et al., Associations Between Nonalcoholic Fatty Liver Disease and Cancers in a Large Cohort in China. Clinical Gastroenterology and Hepatology : the Official Clinical Practice Journal of the American Gastroenterological Association, 2021. 19(4).<br>● Pang, Y., et al., Socioeconomic Status in Relation to Risks of Major Gastrointestinal Cancers in Chinese Adults: A Prospective Study of 0.5 Million People. Cancer Epidemiology, Biomarkers & Prevention : a Publication of the American Association For Cancer Research, Cosponsored by the American Society of Preventive Oncology, 2020. 29(4): p. 823-831.<br>● Li, L., et al., Educational level and colorectal cancer risk: the mediating roles of lifestyle and dietary factors. European Journal of Cancer Prevention : the Official Journal of the European Cancer Prevention Organisation (ECP), 2022. 31(2): p. 137-144.
Static occupation	6	● Song, Y.F., et al., A case control study on the relationship between colorectal cancer and helicobacter pylori Infection and other factors(幽门螺旋杆菌感染等因素与大肠癌关系的病例对照研究). Journal of Modern Oncology, 2007. 15(7).<br>● Wang, X.H., et al., A case-control study of risk factors for colon cancer(结肠癌危险因素的病例对照研究). Chinese Journal of Cancer, 2001(09): p. 977-980.<br>● Whittemore, A.S., et al., Diet, physical activity, and colorectal cancer among Chinese in North America and China. Journal of the National Cancer Institute, 1990. 82(11): p. 915-926.<br>● Liu, A.Z., et al., 153 Paired case control study of risk factors for colorectal cancer(153 对大肠癌危险因素的配对病例对照研究). CHINA PUBLIC HEALTH, 1997. 13(4).<br>● Chen, K., et al., Alcohol drinking and colorectal cancer:a population-based prospective cohort study(应用队列研究方法检验饮酒与肠癌发病的联系). Journal of ZheJiang University (Medical Sciences), 2004(05): p. 40-44.<br>● Chen, K., et al., Association of Drinking Water Source and Colorectal Cancer Incidence: A Prospect Cohort Study(饮水类型与结直肠癌发病率关系的前瞻性队列研究). Chinese Journal of Cancer, 2004(05): p. 550-554.
**Anthropometrics**		
BMI(≥24 kg/m^2)	10	● Ma, H.T., et al., Physical activity and colorectal cancer: a case-control trial(体力活动对结直肠癌发病影响的重庆地区大样本病例-对照研究). Journal of Army Medical University, 2005. 27(22): p. 2276-2279.<br>● Hou, L., et al., Body mass index and colon cancer risk in Chinese people: menopause as an effect modifier. European Journal of Cancer (Oxford, England : 1990), 2006. 42(1): p. 84-90.<br>● Lee, S.A., et al., Animal origin foods and colorectal cancer risk: a report from the Shanghai Women's Health Study. Nutrition and Cancer, 2009. 61(2): p. 194-205.<br>● Wei, Y.S., et al., Risk factors for sporadic colorectal cancer in southern Chinese. World Journal of Gastroenterology, 2009. 15(20): p. 2526-2530.<br>● Yao, J.H., Relationship between body mass index and the colorectal cancer(身体体质指数与结直肠癌的相关性研究). Chinese Journal of Gastroenterology and Hepatology, 2011. 20(10): p. 904-906.<br>● Zhang, D.W., et al., Relationship of patients with type 2 diabetes mellitus with colorectal cancer(II型糖尿病与结直肠癌的相关性研究). Journal of Modern Oncology, 2011. 19(09): p. 1802-1805.<br>● Keskin, H., et al., Colorectal cancer in the Linxian China Nutrition Intervention Trial: Risk factors and intervention results. PloS One, 2021. 16(9): p. e0255322.<br>● Tang, D., et al., Research of genetic and lifestyle risk score on risk assessment of colorectal cancer(遗传和生活方式风险评分与结直肠癌发生风险的评估研究). Journal of Medical Forum, 2021. 42(19): p. 97-101.

		• Wang, Z., et al., Associations Between Nonalcoholic Fatty Liver Disease and Cancers in a Large Cohort in China. Clinical Gastroenterology and Hepatology : the Official Clinical Practice Journal of the American Gastroenterological Association, 2021. 19(4).<br>• Wang W, Dong Z, Zhang X, Li W, Li P, Chen X. Dietary and the Risk of Sporadic Colorectal Cancer in China: A Case-control Study. Iran J Public Health. 2018;47(9):1327-1335.
Waist hip rate	4	• Lee, S.A., et al., Animal origin foods and colorectal cancer risk: a report from the Shanghai Women's Health Study. Nutrition and Cancer, 2009. 61(2): p. 194-205.<br>• Zhang, Q.-L., et al., The joint effects of major lifestyle factors on colorectal cancer risk among Chinese men: A prospective cohort study. International Journal of Cancer, 2018. 142(6): p. 1093-1101.<br>• Li, H., et al., Body weight, fat distribution and colorectal cancer risk: a report from cohort studies of 134255 Chinese men and women. International Journal of Obesity (2005), 2013. 37(6): p. 783-789.<br>• Wang SY, Zhang WS, Jiang CQ, et al. Association of novel and conventional obesity indices with colorectal cancer risk in older Chinese: a 14-year follow-up of the Guangzhou Biobank Cohort Study. BMC Cancer. 2023;23(1):286. Published 2023 Mar 29.
Triglyceride	4	• Wang, Z., et al., Associations Between Nonalcoholic Fatty Liver Disease and Cancers in a Large Cohort in China. Clinical Gastroenterology and Hepatology : the Official Clinical Practice Journal of the American Gastroenterological Association, 2021. 19(4).<br>• Hu, S.Q., Z. Tang, and M. Zhang, Investigation on the risks of colorectal cancer in patients with diabetes mellitus(糖尿病与结直肠癌患病危险关系的调查分析). World Chinese Journal of Digestology, 2007(01): p. 88-91.<br>• Li, X., et al., Metabolic Syndrome Components and the Risk of Colorectal Cancer: A Population-Based Prospective Study in Chinese Men. Frontiers In Oncology, 2019. 9: p. 1047.<br>• Lin XF, Shi KQ, You J, et al. Increased risk of colorectal malignant neoplasm in patients with nonalcoholic fatty liver disease: a large study. Mol Biol Rep. 2014;41(5):2989-2997.
C-reactive protein	3	• Wang, Z., et al., Associations Between Nonalcoholic Fatty Liver Disease and Cancers in a Large Cohort in China. Clinical Gastroenterology and Hepatology : the Official Clinical Practice Journal of the American Gastroenterological Association, 2021. 19(4).<br>• Wu, J., et al., Circulating C-reactive protein and colorectal cancer risk: a report from the Shanghai Men's Health Study. Carcinogenesis, 2013. 34(12): p. 2799-2803.<br>• Wang, G., et al., A prospective cohort study of inflammatory factors and risk of colorectal cancer in a male population(炎性因子与男性人群大肠癌发病风险的前瞻性队列研究). National Medical Journal of China, 2017. 97(40).
**Lifestyle**		
Smoking	26	• Li, L., et al., A Case-Control Study for Colon Cancer in Beijing,China(北京市结肠癌危险因素的病例对照研究). Chinese Journal of Clinical Oncology, 2003(08): p. 26-28.<br>• Wei, Y.S., et al., Risk factors for sporadic colorectal cancer in southern Chinese. World Journal of Gastroenterology, 2009. 15(20): p. 2526-2530.<br>• Zhang, D.W., et al., Relationship of patients with type 2 diabetes mellitus with colorectal cancer(II型糖尿病与结直肠癌的相关性研究). Journal of Modern Oncology, 2011. 19(09): p. 1802-1805.<br>• Keskin, H., et al., Colorectal cancer in the Linxian China Nutrition Intervention Trial: Risk factors and intervention results. PloS One, 2021. 16(9): p. e0255322.<br>• Tang, D., et al., Research of genetic and lifestyle risk score on risk assessment of colorectal cancer(遗传和生活方式风险评分与结直肠癌发生风险的评估研究). Journal of Medical Forum, 2021. 42(19): p. 97-101.<br>• Wang, Z., et al., Associations Between Nonalcoholic Fatty Liver Disease and Cancers in a Large Cohort in China. Clinical Gastroenterology and Hepatology : the Official Clinical Practice Journal of the American Gastroenterological Association, 2021. 19(4).<br>• Wu, D.R., A case-control study on risk factors of colorectal cancer in Jiashan County(嘉善县大肠癌危险因素的病例对照研究). Journal of Practical Oncology, 1990. 5(2).<br>• Peng, X.E., et al., Case-control Study on Risk Factors of Colorectal Cancer in Fujian Province(福建省大肠癌发病危险因素的病例对照研究). Cancer Research on Prevention and Treatment, 2009(9).<br>• Ma, J.J., et al., Study on the incidence and the influencing factors of colorectal cancer in Hanting District of Weifang(潍坊市寒亭区结直肠癌发病情况及影响因素研究). Chinese Journal of Hospital Statistics, 2020. 27(04): p. 349-353.<br>• Yang, G., et al., Dietary Factors and Cancer of the Colonand Rectum in a Population based Case-control Study in Shanghai(结、直肠癌与营养因素的流行病学研究). Chinese Journal of Epidemiology, 1994. 15(5): p. 299-303.<br>• Hu, S.Q., Z. Tang, and M. Zhang, Investigation on the risks of colorectal cancer in patients with diabetes mellitus(糖尿病与结直肠癌患病危险关系的调查分析). World Chinese Journal of Digestology, 2007(01): p. 88-91.<br>• Jiang, Q.T., et al., The case-control study on relationship between environmental risk exposure and incidence of colorectal cancer in the population-based cohort(随访队列的结直肠癌危险因素的病例-对照研究). Tumor, 2004(01): p. 6-10.

		● Yang, T., et al., Intake of Pickled Vegetables and Colorectal Cancer(腌泡菜摄入及其与其他饮食习惯的交互作用与结直肠癌发生风险的关系). Journal of Sichuan University(Medical Science Edition), 2017. 48(06): p. 886-890.<br>● Gao, W.X., et al., Smoking and other related factors and epidemiology of colorectal cancer(吸烟等相关因素与大肠癌的流行病学研究). China Tropical Medicine, 2017. 17(7).<br>● Chang, C., et al., Relationship between the gene-environment factors to colorectal cancer in the Hakka population in Meizhou area(遗传及环境因素与广东省梅州客家人结直肠癌的关系研究). Chinese Journal of Surgical Oncology, 2018. 10(02): p. 87-91.<br>● Chen, K., et al., A case-control study on the association between the genetic polymorphism of sulfotransferase 1A1, diet and susceptibility of colorectal cancer(磺基转移酶 1A1 基因多态性和饮食暴露与结直肠癌易感性的关系). Chinese Journal of Oncology, 2006(09): p. 670-673.<br>● Chen, K., et al., Association of Drinking Water Source and Colorectal Cancer Incidence: A Prospect Cohort Study(饮水类型与结直肠癌发病率关系的前瞻性队列研究). Chinese Journal of Cancer, 2004(05): p. 550-554.<br>● Ji, B.T., et al., Cigarette and alcohol consumption and the risk of colorectal cancer in Shanghai, China. European Journal of Cancer Prevention : the Official Journal of the European Cancer Prevention Organisation (ECP), 2002. 11(3): p. 237-244.<br>● Ho, J.W.-C., et al., Smoking, drinking and colorectal cancer in Hong Kong Chinese: a case-control study. International Journal of Cancer, 2004. 109(4): p. 587-597.<br>● Gong, J.P., et al., Relationship of CYP2E1 RsaI genetic polymorphism and smoking,alcohol drinking habit with risk of rectal cancer(细胞色素 P450 2E1 基因多态性、烟酒习惯与直肠癌易感性的关系). Acta Universitatis Medicinalis Nanjing(Natural Science), 2006. 26(12).<br>● Chen, J., et al., Influencing Factors Analysis of Gene Polymorphisms and Environmental Factors of Rectal Cancer(直肠癌相关基因多态性及环境因素的影响因素分析). Chinese Journal of Health Statistics, 2012. 29(01): p. 31-33.<br>● Liu, K., Effect of environmental factors and gene polymorphisms on colorectal cancer(环境因素及基因多态性对直肠癌的影响). Journal of Colorectal & Anal Surgery, 2015. 21(S1): p. 31-32.<br>● Shuai.Q., et al., Association between plasma 25-hydroxy vitamin D levels and risk of colorectal neoplasms:a case-control study(血浆 25-羟维生素 D 水平与结直肠肿瘤关系的病例对照研究). Academic Journal of Second Military Medical University, 2016. 37(05): p. 536-543.<br>● Li, J., et al., Environmental tobacco smoke and cancer risk, a prospective cohort study in a Chinese population. Environmental Research, 2020. 191: p. 110015.<br>● Lin Y, Peng Y, Liang B, et al. Associations of dinner-to-bed time, post-dinner walk and sleep duration with colorectal cancer: A case-control study. Medicine (Baltimore). 2018;97(34):e12038.<br>● Wu IC, Lee CH, Kuo CH, et al. Consumption of cigarettes but not betel quid or alcohol increases colorectal cancer risk. J Formos Med Assoc. 2009;108(2):155-163.
Alcohol	26	● Zhang, X.Y., et al., An Analysis on Risk Factors of Common Malignant Tumors in Tongxiang City(上海市结肠癌营养流行病学调查). Zhejiang Journal of Preventive Medicine, 2014. 26(02): p. 150-153.<br>● Wei, Y.S., et al., Risk factors for sporadic colorectal cancer in southern Chinese. World Journal of Gastroenterology, 2009. 15(20): p. 2526-2530.<br>● Zhang, D.W., et al., Relationship of patients with type 2 diabetes mellitus with colorectal cancer(II型糖尿病与结直肠癌的相关性研究). Journal of Modern Oncology, 2011. 19(09): p. 1802-1805.<br>● Keskin, H., et al., Colorectal cancer in the Linxian China Nutrition Intervention Trial: Risk factors and intervention results. PloS One, 2021. 16(9): p. e0255322.<br>● Tang, D., et al., Research of genetic and lifestyle risk score on risk assessment of colorectal cancer(遗传和生活方式风险评分与结直肠癌发生风险的评估研究). Journal of Medical Forum, 2021. 42(19): p. 97-101.<br>● Wang, Z., et al., Associations Between Nonalcoholic Fatty Liver Disease and Cancers in a Large Cohort in China. Clinical Gastroenterology and Hepatology : the Official Clinical Practice Journal of the American Gastroenterological Association, 2021. 19(4).<br>● Wu, D.R., A case-control study on risk factors of colorectal cancer in Jiashan County(嘉善县大肠癌危险因素的病例对照研究). Journal of Practical Oncology, 1990. 5(2).<br>● Hu, S.Q., Z. Tang, and M. Zhang, Investigation on the risks of colorectal cancer in patients with diabetes mellitus(糖尿病与结直肠癌患病危险关系的调查分析). World Chinese Journal of Digestology, 2007(01): p. 88-91.<br>● Wang, X.H., et al., A case-control study of risk factors for colon cancer(结肠癌危险因素的病例对照研究). Chinese Journal of Cancer, 2001(09): p. 977-980.<br>● Yang, T., et al., Intake of Pickled Vegetables and Colorectal Cancer(腌泡菜摄入及其与其他饮食习惯的交互作用与结直肠癌发生风险的关系). Journal of Sichuan University(Medical Science Edition), 2017. 48(06): p. 886-890.

		• Zhu, L.P., et al., A Case-control Study of Risk Factors for Colorectal Cancer in Jiangxi Province(江西省 308 例大肠癌危险因素的病例对照研究). Chinese Journal of Prevention and Control of Chronic Non-communicable Diseases, 2001. 9(3).<br>• Zhang, X.H. and A.G. Xu, Analysis on the Risk Factors for Colorectal Cancer(大肠癌危险因素的病例对照研究). Chinese Journal of Clinical Gastroenterology, 2010(5).<br>• Li, X.L., A case-control family study of colorectal cancer in rural area of Taizhou City in Jiangsu Province(江苏省泰兴市农村地区结直肠癌危险因素的病例对照家系研究). International Journal of Pathology and Clinical Medicine, 2013. 33(02): p. 112-115.<br>• Chang, C., et al., Relationship between the gene-environment factors to colorectal cancer in the Hakka population in Meizhou area(遗传及环境因素与广东省梅州客家人结直肠癌的关系研究). Chinese Journal of Surgical Oncology, 2018. 10(02): p. 87-91.<br>• Chen, K., et al., Alcohol drinking and colorectal cancer:a population-based prospective cohort study(应用队列研究方法检验饮酒与肠癌发病的联系). Journal of ZheJiang University (Medical Sciences), 2004(05): p. 40-44.<br>• Ji, B.T., et al., Cigarette and alcohol consumption and the risk of colorectal cancer in Shanghai, China. European Journal of Cancer Prevention : the Official Journal of the European Cancer Prevention Organisation (ECP), 2002. 11(3): p. 237-244.<br>• Ho, J.W.-C., et al., Smoking, drinking and colorectal cancer in Hong Kong Chinese: a case-control study. International Journal of Cancer, 2004. 109(4): p. 587-597.<br>• Gong, J.P., et al., Relationship of CYP2E1 RsaI genetic polymorphism and smoking,alcohol drinking habit with risk of rectal cancer(细胞色素 P450 2E1 基因多态性、烟酒习惯与直肠癌易感性的关系). Acta Universitatis Medicinalis Nanjing(Natural Science), 2006. 26(12).<br>• Shuai.Q., et al., Association between plasma 25-hydroxy vitamin D levels and risk of colorectal neoplasms:a case-control study(血浆 25-羟维生素 D 水平与结直肠肿瘤关系的病例对照研究). Academic Journal of Second Military Medical University, 2016. 37(05): p. 536-543.<br>• Liu, X.Y., et al.,A case-control study of 286 cases with colorectal cancer in Jiashan County(嘉善县 286 例大肠癌病例-对照研究). Chinese Journal of Prevention and Control of Chronic Noncommunicable Disease 1994. 2(3).<br>• Zhou, L., H. Yu, and S. Zheng, Risk-factors analysis of colorectal cancer in Hangzhou(杭州市大肠癌危险因素分析). Journal of Zhejiang University(Medical Sciences), 1996. 25(5).<br>• Gao, C.-M., et al., Polymorphisms in XRCC1 gene, alcohol drinking, and risk of colorectal cancer: a case-control study in Jiangsu Province of China. Asian Pacific Journal of Cancer Prevention : APJCP, 2014. 14(11): p. 6613-6618.<br>• Wang, Y., et al., Association between alcohol consumption and colorectal cancer risk: a case-control study in the Han Chinese population. European Journal of Cancer Prevention : the Official Journal of the European Cancer Prevention Organisation (ECP), 2018. 27(5): p. 433-437.<br>• Im, P.K., et al., Alcohol drinking and risks of total and site-specific cancers in China: A 10-year prospective study of 0.5 million adults. International Journal of Cancer, 2021. 149(3): p. 522-534.<br>• Lin Y, Peng Y, Liang B, et al. Associations of dinner-to-bed time, post-dinner walk and sleep duration with colorectal cancer: A case-control study. Medicine (Baltimore). 2018;97(34):e12038.<br>• Wu IC, Lee CH, Kuo CH, et al. Consumption of cigarettes but not betel quid or alcohol increases colorectal cancer risk. J Formos Med Assoc. 2009;108(2):155-163.
Tea	12	• Lee, S.A., et al., Animal origin foods and colorectal cancer risk: a report from the Shanghai Women's Health Study. Nutrition and Cancer, 2009. 61(2): p. 194-205.<br>• Ma, J.J., et al., Study on the incidence and the influencing factors of colorectal cancer in Hanting District of Weifang(潍坊市寒亭区结直肠癌发病情况及影响因素研究). Chinese Journal of Hospital Statistics, 2020. 27(04): p. 349-353.<br>• Zhang, T., et al., Analysis of the related factors between lifestyle and colorectal cancer in Nanyang area(南阳地区居民生活方式与结直肠癌相关因素分析). Journal of Community Medicine, 2020. 18(18): p. 1255-1258.<br>• Yang, G., et al., Dietary Factors and Cancer of the Colonand Rectum in a Population based Case-control Study in Shanghai(结、直肠癌与营养因素的流行病学研究). Chinese Journal of Epidemiology, 1994. 15(5): p. 299-303.<br>• Yang, T., et al., Intake of Pickled Vegetables and Colorectal Cancer(腌泡菜摄入及其与其他饮食习惯的交互作用与结直肠癌发生风险的关系). Journal of Sichuan University(Medical Science Edition), 2017. 48(06): p. 886-890.<br>• Ji, B.T., et al., Green tea consumption and the risk of pancreatic and colorectal cancers. International Journal of Cancer, 1997. 70(3): p. 255-258.<br>• Ho, J., S. Yuen, and S. Yuen, A case-control study on environmental and familial risk factors for colorectal cancer in Hong Kong: physical activity reduces colorectal cancer risk. 2006.<br>• Yang, G., et al., Prospective cohort study of green tea consumption and colorectal cancer risk in women. Cancer Epidemiology, Biomarkers & Prevention : a Publication of the American

		Association For Cancer Research, Cosponsored by the American Society of Preventive Oncology, 2007. 16(6): p. 1219-1223.<br>● Yang, G., et al., Green tea consumption and colorectal cancer risk: a report from the Shanghai Men's Health Study. Carcinogenesis, 2011. 32(11): p. 1684-1688.<br>● Nechuta, S., et al., Prospective cohort study of tea consumption and risk of digestive system cancers: results from the Shanghai Women's Health Study. The American Journal of Clinical Nutrition, 2012. 96(5): p. 1056-1063.<br>● Wu, X., et al., Green Tea Consumption and Colorectal Cancer Risk:a Case-Control Study in Northeast China. Journal of China Medical University, 2018. 47(12): p. 1057-1062.<br>● Li, X., et al., Association between tea consumption and risk of cancer: a prospective cohort study of 0.5 million Chinese adults. European Journal of Epidemiology, 2019. 34(8): p. 753-763.
Irregular bowel movement	3	● Zhang, X.H. and A.G. Xu, Analysis on the Risk Factors for Colorectal Cancer(大肠癌危险因素的病例对照研究). Chinese Journal of Clinical Gastroenterology, 2010(5).<br>● Zhang, X.H. and A.G. Xu, Analysis on the risk factors for colorectal cancer in Huizhou city(大肠癌危险因素分析). Modern Digestion & Intervention, 2005(04): p. 203-205.<br>● YandgS.C., et al., Prospective association between bowel frequency and risk of colorectal cancer in Chinese adults(中国成年人排便频率与结直肠癌发病风险的前瞻性关联分析). Chinese Journal of Epidemiology, 2019. 40(04): p. 382-388.
Sedentariness	8	● Feng, Y.J., A paired case-control study of risk factors for rectal cancer(直肠癌危险因素的配对病例对照研究). Journal of Chinese Physician, 2008(12): p. 1690-1691.<br>● Zhao, J.S., et al., A Case-control Study on Risk Factors of Colonic Cancer(结肠癌危险因素的病例对照研究). Cancer Research on Prevention and Treatment, 2008(07): p. 524-526.<br>● Ma, J.J., et al., Study on the incidence and the influencing factors of colorectal cancer in Hanting District of Weifang(潍坊市寒亭区结直肠癌发病情况及影响因素研究). Chinese Journal of Hospital Statistics, 2020. 27(04): p. 349-353.<br>● Zhang, T., et al., Analysis of the related factors between lifestyle and colorectal cancer in Nanyang area(南阳地区居民生活方式与结直肠癌相关因素分析). Journal of Community Medicine, 2020. 18(18): p. 1255-1258.<br>● Yang, T., et al., Intake of Pickled Vegetables and Colorectal Cancer(腌泡菜摄入及其与其他饮食习惯的交互作用与结直肠癌发生风险的关系). Journal of Sichuan University(Medical Science Edition), 2017. 48(06): p. 886-890.<br>● Lai, K.D., et al., Diet physical activity and colorectal cancer: case control study in Dalian(膳食、体力活动与大肠癌:病例对照研究). Chinese Journal of Prevention and Control of Chronic Diseases, 1995. 3(3).<br>● Whittemore, A.S., et al., Diet, physical activity, and colorectal cancer among Chinese in North America and China. Journal of the National Cancer Institute, 1990. 82(11): p. 915-926.<br>● Yuan, P., et al., Association of dietary fiber intake with colorectal cancer: a matched casecontrol study(结直肠癌与膳食纤维相关饮食因素病例对照研究). Chinese Journal of Public Health, 2016. 32(12): p. 1719-1723.
Moderate physical activity	4	● Ma, H.T., et al., Physical activity and colorectal cancer: a case-control trial(体力活动对结直肠癌发病影响的重庆地区大样本病例-对照研究). Journal of Army Medical University, 2005. 27(22): p. 2276-2279.<br>● Zhang, C., R.T. Wang, and T.G. Wang, A case-control study of 250 cases of colorectal cancer in Beijing(北京市 250 例大肠癌的病例对照研究). Chinese Journal of Epidemiology, 1992. 13(6).<br>● Hou, L., et al., Commuting physical activity and risk of colon cancer in Shanghai, China. American Journal of Epidemiology, 2004. 160(9): p. 860-867.<br>● Tang R, Wang JY, Lo SK, Hsieh LL. Physical activity, water intake and risk of colorectal cancer in Taiwan: a hospital-based case-control study. Int J Cancer. 1999;82(4):484-489.
Heavy physical activity	4	● Ma, H.T., et al., Physical activity and colorectal cancer: a case-control trial(体力活动对结直肠癌发病影响的重庆地区大样本病例-对照研究). Journal of Army Medical University, 2005. 27(22): p. 2276-2279.<br>● Zhang, C., R.T. Wang, and T.G. Wang, A case-control study of 250 cases of colorectal cancer in Beijing(北京市 250 例大肠癌的病例对照研究). Chinese Journal of Epidemiology, 1992. 13(6).<br>● Hou, L., et al., Commuting physical activity and risk of colon cancer in Shanghai, China. American Journal of Epidemiology, 2004. 160(9): p. 860-867.<br>● Tang R, Wang JY, Lo SK, Hsieh LL. Physical activity, water intake and risk of colorectal cancer in Taiwan: a hospital-based case-control study. Int J Cancer. 1999;82(4):484-489.
Exercise	9	● Ma, H.T., et al., Physical activity and colorectal cancer: a case-control trial(体力活动对结直肠癌发病影响的重庆地区大样本病例-对照研究). Journal of Army Medical University, 2005. 27(22): p. 2276-2279.<br>● Lee, S.A., et al., Animal origin foods and colorectal cancer risk: a report from the Shanghai Women's Health Study. Nutrition and Cancer, 2009. 61(2): p. 194-205.

		● Tang, D., et al., Research of genetic and lifestyle risk score on risk assessment of colorectal cancer(遗传和生活方式风险评分与结直肠癌发生风险的评估研究). Journal of Medical Forum, 2021. 42(19): p. 97-101.<br>● Feng, Y.J., A paired case-control study of risk factors for rectal cancer(直肠癌危险因素的配对病例对照研究). Journal of Chinese Physician, 2008(12): p. 1690-1691.<br>● Wang, F.J., L.F. Jiang, and J.F. Guo, A case-control study on the risk factors for colorectal cancer in Xiaoshan county(萧山区大肠癌发病危险因素的病例对照研究). Chinese Rural Health Service Administration, 2012. 32(9).<br>● Zhang, T., et al., Analysis of the related factors between lifestyle and colorectal cancer in Nanyang area(南阳地区居民生活方式与结直肠癌相关因素分析). Journal of Community Medicine, 2020. 18(18): p. 1255-1258.<br>● Zhang, Q.-L., et al., The joint effects of major lifestyle factors on colorectal cancer risk among Chinese men: A prospective cohort study. International Journal of Cancer, 2018. 142(6): p. 1093-1101.<br>● Su J, Jiang Y, Fan X, et al. Association between physical activity and cancer risk among Chinese adults: a 10-year prospective study. Int J Behav Nutr Phys Act. 2022;19(1):150.<br>● Wang W, Dong Z, Zhang X, Li W, Li P, Chen X. Dietary and the Risk of Sporadic Colorectal Cancer in China: A Case-control Study. Iran J Public Health. 2018;47(9):1327-1335.
**Dietary intake**		
Milk and dairy products	6	● Li, L., et al., A Case-Control Study for Colon Cancer in Beijing,China(北京市结肠癌危险因素的病例对照研究). Chinese Journal of Clinical Oncology, 2003(08): p. 26-28.<br>● Lee, S.A., et al., Animal origin foods and colorectal cancer risk: a report from the Shanghai Women's Health Study. Nutrition and Cancer, 2009. 61(2): p. 194-205.<br>● Chiu, B.C.H., et al., Dietary factors and risk of colon cancer in Shanghai, China. Cancer Epidemiology, Biomarkers & Prevention : a Publication of the American Association For Cancer Research, Cosponsored by the American Society of Preventive Oncology, 2003. 12(3): p. 201-208.<br>● Che, Q.H. and H.R. Diao, A Study on the Correlation of Dietary Factors with Colorectal Cancer in the Patients from a Hospital of Shenyang(结直肠癌患者膳食相关因素病例对照研究). Chinese Journal of Prevention and Control of Chronic Diseases, 2010. 18(04): p. 368-369.<br>● Zhang, X., et al., Higher intakes of dietary vitamin D, calcium and dairy products are inversely associated with the risk of colorectal cancer: a case-control study in China. The British Journal of Nutrition, 2020. 123(6): p. 699-711.<br>● Kakkoura MG, Du H, Guo Y, et al. Dairy consumption and risks of total and site-specific cancers in Chinese adults: an 11-year prospective study of 0.5 million people. BMC Med. 2022;20(1):134.
Fruit and vegetables	21	● Li, L., et al., A Case-Control Study for Colon Cancer in Beijing,China(北京市结肠癌危险因素的病例对照研究). Chinese Journal of Clinical Oncology, 2003(08): p. 26-28.<br>● Lee, S.A., et al., Animal origin foods and colorectal cancer risk: a report from the Shanghai Women's Health Study. Nutrition and Cancer, 2009. 61(2): p. 194-205.<br>● Keskin, H., et al., Colorectal cancer in the Linxian China Nutrition Intervention Trial: Risk factors and intervention results. PloS One, 2021. 16(9): p. e0255322.<br>● Zhang, C., R.T. Wang, and T.G. Wang, A case-control study of 250 cases of colorectal cancer in Beijing(北京市 250 例大肠癌的病例对照研究). Chinese Journal of Epidemiology, 1992. 13(6).<br>● Feng, Y.J., A paired case-control study of risk factors for rectal cancer(直肠癌危险因素的配对病例对照研究). Journal of Chinese Physician, 2008(12): p. 1690-1691.<br>● Zhao, J.S., et al., A Case-control Study on Risk Factors of Colonic Cancer(结肠癌危险因素的病例对照研究). Cancer Research on Prevention and Treatment, 2008(07): p. 524-526.<br>● Ma, J.J., et al., Study on the incidence and the influencing factors of colorectal cancer in Hanting District of Weifang(潍坊市寒亭区结直肠癌发病情况及影响因素研究). Chinese Journal of Hospital Statistics, 2020. 27(04): p. 349-353.<br>● Wang, X.H., et al., A case-control study of risk factors for colon cancer(结肠癌危险因素的病例对照研究). Chinese Journal of Cancer, 2001(09): p. 977-980.<br>● Wang, X.H., et al., Colon cancer risk factors in Jiashan county, Zhejiang province, the highest incidence area in China(大肠癌高发区居民结肠癌危险因素研究). Chinese Journal of Oncology, 2001(06): p. 44-46.<br>● Chiu, B.C.H., et al., Dietary factors and risk of colon cancer in Shanghai, China. Cancer Epidemiology, Biomarkers & Prevention : a Publication of the American Association For Cancer Research, Cosponsored by the American Society of Preventive Oncology, 2003. 12(3): p. 201-208.<br>● Zhang, Z.Y., et al., A case-control study of 269 cases of colorectal cancer(269 例大肠癌病例对照研究). Chinese Journal of Cancer Prevention and Treatment, 2002. 9(6).<br>● Vogtmann, E., et al., Fruit and vegetable intake and the risk of colorectal cancer: results from the Shanghai Men's Health Study. CanceVogtmann, E., et al., Fruit and vegetable intake and

		the risk of colorectal cancer: results from the Shanghai Men's Health Study. Cancer Causes & Control : CCC, 2013. 24(11): p. 1935-1945.<br>● Liu, Y.T., J.H. Ding, and Y.G. Lin, A case-control study of rectal cancer(直肠癌病例对照研究). Jiangsu Medical Journal, 1993(04): p. 218.<br>● Che, Q.H. and H.R. Diao, A Study on the Correlation of Dietary Factors with Colorectal Cancer in the Patients from a Hospital of Shenyang(结直肠癌患者膳食相关因素病例对照研究). Chinese Journal of Prevention and Control of Chronic Diseases, 2010. 18(04): p. 368-369.<br>● Vogtmann, E., et al., Cruciferous vegetables, glutathione S-transferase polymorphisms, and the risk of colorectal cancer among Chinese men. Annals of Epidemiology, 2014. 24(1): p. 44-49.<br>● Luo, W.-P., et al., High consumption of vegetable and fruit colour groups is inversely associated with the risk of colorectal cancer: a case-control study. The British Journal of Nutrition, 2015. 113(7): p. 1129-1138.<br>● Qin, M., et al., Risk factors for colorectal neoplasms based on colonoscopy and pathological diagnoses of Chinese citizens: a multicenter, case-control study. International Journal of Colorectal Disease, 2015. 30(3): p. 353-361.<br>● Fang, W., et al., Cruciferous vegetables and colorectal cancer risk: a hospital-based matched case-control study in Northeast China. European Journal of Clinical Nutrition, 2019. 73(3): p. 450-457.<br>● Wu, X., et al., Allium vegetables are associated with reduced risk of colorectal cancer: A hospital-based matched case-control study in China. Asia-Pacific Journal of Clinical Oncology, 2019. 15(5): p. e132-e141.<br>● Liu Y, Li S, Jiang L, Zhang Y, Li Z, Shi J. Solanaceous Vegetables and Colorectal Cancer Risk: A Hospital-Based Matched Case-Control Study in Northeast China. Front Nutr. 2021;8:688897.<br>● Wang W, Dong Z, Zhang X, Li W, Li P, Chen X. Dietary and the Risk of Sporadic Colorectal Cancer in China: A Case-control Study. Iran J Public Health. 2018;47(9):1327-1335.
Vegetables	19	● Li, L., et al., A Case-Control Study for Colon Cancer in Beijing,China(北京市结肠癌危险因素的病例对照研究). Chinese Journal of Clinical Oncology, 2003(08): p. 26-28.<br>● Keskin, H., et al., Colorectal cancer in the Linxian China Nutrition Intervention Trial: Risk factors and intervention results. PloS One, 2021. 16(9): p. e0255322.<br>● Zhang, C., R.T. Wang, and T.G. Wang, A case-control study of 250 cases of colorectal cancer in Beijing(北京市 250 例大肠癌的病例对照研究). Chinese Journal of Epidemiology, 1992. 13(6).<br>● Feng, Y.J., A paired case-control study of risk factors for rectal cancer(直肠癌危险因素的配对病例对照研究). Journal of Chinese Physician, 2008(12): p. 1690-1691.<br>● Zhao, J.S., et al., A Case-control Study on Risk Factors of Colonic Cancer(结肠癌危险因素的病例对照研究). Cancer Research on Prevention and Treatment, 2008(07): p. 524-526.<br>● Ma, J.J., et al., Study on the incidence and the influencing factors of colorectal cancer in Hanting District of Weifang(潍坊市寒亭区结直肠癌发病情况及影响因素研究). Chinese Journal of Hospital Statistics, 2020. 27(04): p. 349-353.<br>● Wang, X.H., et al., A case-control study of risk factors for colon cancer(结肠癌危险因素的病例对照研究). Chinese Journal of Cancer, 2001(09): p. 977-980.<br>● Wang, X.H., et al., Colon cancer risk factors in Jiashan county, Zhejiang province, the highest incidence area in China(大肠癌高发区居民结肠癌危险因素研究). Chinese Journal of Oncology, 2001(06): p. 44-46.<br>● Chiu, B.C.H., et al., Dietary factors and risk of colon cancer in Shanghai, China. Cancer Epidemiology, Biomarkers & Prevention : a Publication of the American Association For Cancer Research, Cosponsored by the American Society of Preventive Oncology, 2003. 12(3): p. 201-208.<br>● Zhang, Z.Y., et al., A case-control study of 269 cases of colorectal cancer(269 例大肠癌病例对照研究). Chinese Journal of Cancer Prevention and Treatment, 2002. 9(6).<br>● Vogtmann, E., et al., Fruit and vegetable intake and the risk of colorectal cancer: results from the Shanghai Men's Health Study. CanceVogtmann, E., et al., Fruit and vegetable intake and the risk of colorectal cancer: results from the Shanghai Men's Health Study. Cancer Causes & Control : CCC, 2013. 24(11): p. 1935-1945.<br>● Liu, Y.T., J.H. Ding, and Y.G. Lin, A case-control study of rectal cancer(直肠癌病例对照研究). Jiangsu Medical Journal, 1993(04): p. 218.<br>● Vogtmann, E., et al., Cruciferous vegetables, glutathione S-transferase polymorphisms, and the risk of colorectal cancer among Chinese men. Annals of Epidemiology, 2014. 24(1): p. 44-49.<br>● Luo, W.-P., et al., High consumption of vegetable and fruit colour groups is inversely associated with the risk of colorectal cancer: a case-control study. The British Journal of Nutrition, 2015. 113(7): p. 1129-1138.<br>● Qin, M., et al., Risk factors for colorectal neoplasms based on colonoscopy and pathological diagnoses of Chinese citizens: a multicenter, case-control study. International Journal of Colorectal Disease, 2015. 30(3): p. 353-361.

		● Fang, W., et al., Cruciferous vegetables and colorectal cancer risk: a hospital-based matched case-control study in Northeast China. European Journal of Clinical Nutrition, 2019. 73(3): p. 450-457.<br>● Wu, X., et al., Allium vegetables are associated with reduced risk of colorectal cancer: A hospital-based matched case-control study in China. Asia-Pacific Journal of Clinical Oncology, 2019. 15(5): p. e132-e141.<br>● Liu Y, Li S, Jiang L, Zhang Y, Li Z, Shi J. Solanaceous Vegetables and Colorectal Cancer Risk: A Hospital-Based Matched Case-Control Study in Northeast China. Front Nutr. 2021;8:688897.<br>● Wang W, Dong Z, Zhang X, Li W, Li P, Chen X. Dietary and the Risk of Sporadic Colorectal Cancer in China: A Case-control Study. Iran J Public Health. 2018;47(9):1327-1335.
Fruits	5	● Keskin, H., et al., Colorectal cancer in the Linxian China Nutrition Intervention Trial: Risk factors and intervention results. PloS One, 2021. 16(9): p. e0255322.<br>● Chiu, B.C.H., et al., Dietary factors and risk of colon cancer in Shanghai, China. Cancer Epidemiology, Biomarkers & Prevention : a Publication of the American Association For Cancer Research, Cosponsored by the American Society of Preventive Oncology, 2003. 12(3): p. 201-208.<br>● Vogtmann, E., et al., Fruit and vegetable intake and the risk of colorectal cancer: results from the Shanghai Men's Health Study. CanceVogtmann, E., et al., Fruit and vegetable intake and the risk of colorectal cancer: results from the Shanghai Men's Health Study. Cancer Causes & Control : CCC, 2013. 24(11): p. 1935-1945.<br>● Che, Q.H. and H.R. Diao, A Study on the Correlation of Dietary Factors with Colorectal Cancer in the Patients from a Hospital of Shenyang(结直肠癌患者膳食相关因素病例对照研究). Chinese Journal of Prevention and Control of Chronic Diseases, 2010. 18(04): p. 368-369.<br>● Luo, W.-P., et al., High consumption of vegetable and fruit colour groups is inversely associated with the risk of colorectal cancer: a case-control study. The British Journal of Nutrition, 2015. 113(7): p. 1129-1138.
Calcium	8	● Xiong, J.Y., D.J. Shen, and F.Y. Zhu, Epidemological Study on Hihg Risk Factos for Large Intestine Cancer in Loudi City(娄底市大肠癌高危因素临床流行病学研究). Journal of Chinese Physician, 2002. 4(4).<br>● Yang, G., et al., Environmental and genetic factors in the pathogenesis of colorectal cancer(大肠癌发病的环境因素与遗传因素). Chinese Journal of Epidemiology, 1992. 13(1).<br>● Shin, A., et al., Dietary intake of calcium, fiber and other micronutrients in relation to colorectal cancer risk: Results from the Shanghai Women's Health Study. International Journal of Cancer, 2006. 119(12): p. 2938-2942.<br>● Zhang, X., et al., Higher intakes of dietary vitamin D, calcium and dairy products are inversely associated with the risk of colorectal cancer: a case-control study in China. The British Journal of Nutrition, 2020. 123(6): p. 699-711.<br>● Yang, G., et al., A Case-control Study on Colorectal Cancer and Dietary Fiber and Calcium of Various Sources(不同来源膳食纤维、钙与结直肠癌关系的研究). CHINESE JOURNAL OF PREVENTIVE MEDICINE, 1994. 28(4): p. 195-198.<br>● Yang, G. and B.T. Ji, Relationship between ten inorganic elements and colorectal cancer(十种无机元素与结,直肠癌的关系). Chinese Journal of Preventive Medicine, 1993. 027(5): p. 282-285.<br>● Xiang, J., et al., Dietary Intake of Microelements and Colorectal Cancer Risk(饮食微量元素摄取与结直肠癌的发病风险). China Cancer, 2011. 20(10): p. 731-734.<br>● Liu Y, Li S, Jiang L, Zhang Y, Li Z, Shi J. Solanaceous Vegetables and Colorectal Cancer Risk: A Hospital-Based Matched Case-Control Study in Northeast China. Front Nutr. 2021;8:688897.
Iron	3	● Yang, G. and B.T. Ji, Relationship between ten inorganic elements and colorectal cancer(十种无机元素与结,直肠癌的关系). Chinese Journal of Preventive Medicine, 1993. 027(5): p. 282-285.<br>● Xiang, J., et al., Dietary Intake of Microelements and Colorectal Cancer Risk(饮食微量元素摄取与结直肠癌的发病风险). China Cancer, 2011. 20(10): p. 731-734.<br>● Luo, H., et al., Association between Dietary Zinc and Selenium Intake, Oxidative Stress-Related Gene Polymorphism, and Colorectal Cancer Risk in Chinese Population - A Case-Control Study. Nutrition and Cancer, 2021. 73(9): p. 1621-1630.
Znic	3	● Yang, G. and B.T. Ji, Relationship between ten inorganic elements and colorectal cancer(十种无机元素与结、直肠癌的关系). Chinese Journal of Preventive Medicine, 1993. 027(5): p. 282-285.<br>● Xiang, J., et al., Dietary Intake of Microelements and Colorectal Cancer Risk(饮食微量元素摄取与结直肠癌的发病风险). China Cancer, 2011. 20(10): p. 731-734.<br>● Luo, H., et al., Association between Dietary Zinc and Selenium Intake, Oxidative Stress-Related Gene Polymorphism, and Colorectal Cancer Risk in Chinese Population - A Case-Control Study. Nutrition and Cancer, 2021. 73(9): p. 1621-1630.

Selenium	3	• Yang, G. and B.T. Ji, Relationship between ten inorganic elements and colorectal cancer(十种无机元素与结、直肠癌的关系). Chinese Journal of Preventive Medicine, 1993. 027(5): p. 282-285.<br>• Xiang, J., et al., Dietary Intake of Microelements and Colorectal Cancer Risk(饮食微量元素摄取与结直肠癌的发病风险). China Cancer, 2011. 20(10): p. 731-734.<br>• Luo, H., et al., Association between Dietary Zinc and Selenium Intake, Oxidative Stress-Related Gene Polymorphism, and Colorectal Cancer Risk in Chinese Population - A Case-Control Study. Nutrition and Cancer, 2021. 73(9): p. 1621-1630.
Folate	4	• Chen, K., et al., Folate, methionine, polymorphism of methionine synthase, thymidylate synthetase and susceptibility to colorectal cancer(叶酸、蛋氨酸摄入量,MTR、TS 基因多态与结直肠癌关系的巢式病例对照研究). Acta Nutrimenta Sinica, 2006. 28(4): p. 342-345,349.<br>• Takata, Y., et al., Plasma folate concentrations and colorectal cancer risk: a case-control study nested within the Shanghai Men's Health Study. International Journal of Cancer, 2014. 135(9): p. 2191-2198.<br>• Huang, C.-Y., et al., Dietary B vitamin and methionine intakes and risk for colorectal cancer: a case-control study in China. The British Journal of Nutrition, 2020. 123(11): p. 1277-1289.<br>• Liu Y, Li S, Jiang L, Zhang Y, Li Z, Shi J. Solanaceous Vegetables and Colorectal Cancer Risk: A Hospital-Based Matched Case-Control Study in Northeast China. Front Nutr. 2021;8:688897.
Eggs	5	• Lee, S.A., et al., Animal origin foods and colorectal cancer risk: a report from the Shanghai Women's Health Study. Nutrition and Cancer, 2009. 61(2): p. 194-205.<br>• Keskin, H., et al., Colorectal cancer in the Linxian China Nutrition Intervention Trial: Risk factors and intervention results. PloS One, 2021. 16(9): p. e0255322.<br>• Chiu, B.C.H., et al., Dietary factors and risk of colon cancer in Shanghai, China. Cancer Epidemiology, Biomarkers & Prevention : a Publication of the American Association For Cancer Research, Cosponsored by the American Society of Preventive Oncology, 2003. 12(3): p. 201-208.<br>• Zhu, L.P., et al., A Case-control Study of Risk Factors for Colorectal Cancer in Jiangxi Province(江西省 308 例大肠癌危险因素的病例对照研究). Chinese Journal of Prevention and Control of Chronic Non-communicable Diseases, 2001. 9(3).<br>• Zhang, Z.Y., et al., A case-control study of 269 cases of colorectal cancer(269 例大肠癌病例对照研究). Chinese Journal of Cancer Prevention and Treatment, 2002. 9(6).
All meat	10	• Lee, S.A., et al., Animal origin foods and colorectal cancer risk: a report from the Shanghai Women's Health Study. Nutrition and Cancer, 2009. 61(2): p. 194-205.<br>• Keskin, H., et al., Colorectal cancer in the Linxian China Nutrition Intervention Trial: Risk factors and intervention results. PloS One, 2021. 16(9): p. e0255322.<br>• Zhang, C., R.T. Wang, and T.G. Wang, A case-control study of 250 cases of colorectal cancer in Beijing(北京市 250 例大肠癌的病例对照研究). Chinese Journal of Epidemiology, 1992. 13(6).<br>• Meng, F.H., et al., Investigation on risk factors of colorectal cancer in Guangdong. ACAD J ISI MED COLL PLA, 1994. 14(4).<br>• Wang, X.H., et al., Colon cancer risk factors in Jiashan county, Zhejiang province, the highest incidence area in China(大肠癌高发区居民结肠癌危险因素研究). Chinese Journal of Oncology, 2001(06): p. 44-46.<br>• Chiu, B.C.H., et al., Dietary factors and risk of colon cancer in Shanghai, China. Cancer Epidemiology, Biomarkers & Prevention : a Publication of the American Association For Cancer Research, Cosponsored by the American Society of Preventive Oncology, 2003. 12(3): p. 201-208.<br>• Zhu, L.P., et al., A Case-control Study of Risk Factors for Colorectal Cancer in Jiangxi Province(江西省 308 例大肠癌危险因素的病例对照研究). Chinese Journal of Prevention and Control of Chronic Non-communicable Diseases, 2001. 9(3).<br>• Chen, K., et al., A case-control study on the association between the genetic polymorphism of sulfotransferase 1A1, diet and susceptibility of colorectal cancer(磺基转移酶 1A1 基因多态性和饮食暴露与结直肠癌易感性的关系). Chinese Journal of Oncology, 2006(09): p. 670-673.<br>• Yuan, P., et al., Association of dietary fiber intake with colorectal cancer: a matched casecontrol study(结直肠癌与膳食纤维相关饮食因素病例对照研究). Chinese Journal of Public Health, 2016. 32(12): p. 1719-1723.<br>• Wang W, Dong Z, Zhang X, Li W, Li P, Chen X. Dietary and the Risk of Sporadic Colorectal Cancer in China: A Case-control Study. Iran J Public Health. 2018;47(9):1327-1335.
Red meat	8	• Lee, S.A., et al., Animal origin foods and colorectal cancer risk: a report from the Shanghai Women's Health Study. Nutrition and Cancer, 2009. 61(2): p. 194-205.<br>• Meng, F.H., et al., Investigation on risk factors of colorectal cancer in Guangdong(广东人大肠癌危险因素的调查). ACAD J ISI MED COLL PLA, 1994. 14(4).

		● Wang, X.H., et al., Colon cancer risk factors in Jiashan county, Zhejiang province, the highest incidence area in China(大肠癌高发区居民结肠癌危险因素研究). Chinese Journal of Oncology, 2001(06): p. 44-46.<br>● Chiu, B.C.H., et al., Dietary factors and risk of colon cancer in Shanghai, China. Cancer Epidemiology, Biomarkers & Prevention : a Publication of the American Association For Cancer Research, Cosponsored by the American Society of Preventive Oncology, 2003. 12(3): p. 201-208.<br>● Zhu, L.P., et al., A Case-control Study of Risk Factors for Colorectal Cancer in Jiangxi Province(江西省 308 例大肠癌危险因素的病例对照研究). Chinese Journal of Prevention and Control of Chronic Non-communicable Diseases, 2001. 9(3).<br>● Chen, K., et al., A case-control study on the association between the genetic polymorphism of sulfotransferase 1A1, diet and susceptibility of colorectal cancer(磺基转移酶 1A1 基因多态性和饮食暴露与结直肠癌易感性的关系). Chinese Journal of Oncology, 2006(09): p. 670-673.<br>● Yuan, P., et al., Association of dietary fiber intake with colorectal cancer: a matched casecontrol study(结直肠癌与膳食纤维相关饮食因素病例对照研究). Chinese Journal of Public Health, 2016. 32(12): p. 1719-1723.<br>● Wang W, Dong Z, Zhang X, Li W, Li P, Chen X. Dietary and the Risk of Sporadic Colorectal Cancer in China: A Case-control Study. Iran J Public Health. 2018;47(9):1327-1335.
Carotenoid	4	● Chiu, B.C.H., et al., Dietary factors and risk of colon cancer in Shanghai, China. Cancer Epidemiology, Biomarkers & Prevention : a Publication of the American Association For Cancer Research, Cosponsored by the American Society of Preventive Oncology, 2003. 12(3): p. 201-208.<br>● Shin, A., et al., Dietary intake of calcium, fiber and other micronutrients in relation to colorectal cancer risk: Results from the Shanghai Women's Health Study. International Journal of Cancer, 2006. 119(12): p. 2938-2942.<br>● Lu, M.S., et al., Higher intake of carotenoid is associated with a lower risk of colorectal cancer in Chinese adults: a case-control study. European Journal of Nutrition, 2015. 54(4): p. 619-628.<br>● Liu Y, Li S, Jiang L, Zhang Y, Li Z, Shi J. Solanaceous Vegetables and Colorectal Cancer Risk: A Hospital-Based Matched Case-Control Study in Northeast China. Front Nutr. 2021;8:688897.
VitaminA	5	● lu, R.F., An epidemiological nutrition approach to colon cancer in urban Shanghai(上海市结肠癌营养流行病学调查). Fudan University Journal of Medical Sciences, 1986(5).<br>● Chiu, B.C.H., et al., Dietary factors and risk of colon cancer in Shanghai, China. Cancer Epidemiology, Biomarkers & Prevention : a Publication of the American Association For Cancer Research, Cosponsored by the American Society of Preventive Oncology, 2003. 12(3): p. 201-208.<br>● Shin, A., et al., Dietary intake of calcium, fiber and other micronutrients in relation to colorectal cancer risk: Results from the Shanghai Women's Health Study. International Journal of Cancer, 2006. 119(12): p. 2938-2942.<br>● Luo, H., et al., Dietary and serum vitamins A and E and colorectal cancer risk in Chinese population: a case-control study. European Journal of Cancer Prevention : the Official Journal of the European Cancer Prevention Organisation (ECP), 2019. 28(4): p. 268-277.<br>● Liu Y, Li S, Jiang L, Zhang Y, Li Z, Shi J. Solanaceous Vegetables and Colorectal Cancer Risk: A Hospital-Based Matched Case-Control Study in Northeast China. Front Nutr. 2021;8:688897.
VitaminE	4	● Chiu, B.C.H., et al., Dietary factors and risk of colon cancer in Shanghai, China. Cancer Epidemiology, Biomarkers & Prevention : a Publication of the American Association For Cancer Research, Cosponsored by the American Society of Preventive Oncology, 2003. 12(3): p. 201-208.<br>● Shin, A., et al., Dietary intake of calcium, fiber and other micronutrients in relation to colorectal cancer risk: Results from the Shanghai Women's Health Study. International Journal of Cancer, 2006. 119(12): p. 2938-2942.<br>● Luo, H., et al., Dietary and serum vitamins A and E and colorectal cancer risk in Chinese population: a case-control study. European Journal of Cancer Prevention : the Official Journal of the European Cancer Prevention Organisation (ECP), 2019. 28(4): p. 268-277.<br>● Liu Y, Li S, Jiang L, Zhang Y, Li Z, Shi J. Solanaceous Vegetables and Colorectal Cancer Risk: A Hospital-Based Matched Case-Control Study in Northeast China. Front Nutr. 2021;8:688897.
VitaminC	5	● lu, R.F., An epidemiological nutrition approach to colon cancer in urban Shanghai(上海市结肠癌营养流行病学调查). Fudan University Journal of Medical Sciences, 1986(5).<br>● Yang, G., et al., Environmental and genetic factors in the pathogenesis of colorectal cancer(大肠癌发病的环境因素与遗传因素). Chinese Journal of Epidemiology, 1992. 13(1).<br>● Chiu, B.C.H., et al., Dietary factors and risk of colon cancer in Shanghai, China. Cancer Epidemiology, Biomarkers & Prevention : a Publication of the American Association For Cancer Research, Cosponsored by the American Society of Preventive Oncology, 2003. 12(3): p. 201-208.

		● Shin, A., et al., Dietary intake of calcium, fiber and other micronutrients in relation to colorectal cancer risk: Results from the Shanghai Women's Health Study. International Journal of Cancer, 2006. 119(12): p. 2938-2942.<br>● Liu Y, Li S, Jiang L, Zhang Y, Li Z, Shi J. Solanaceous Vegetables and Colorectal Cancer Risk: A Hospital-Based Matched Case-Control Study in Northeast China. Front Nutr. 2021;8:688897.
VitaminB	3	● Huang CY, Abulimiti A, Zhang X, et al. Dietary B vitamin and methionine intakes and risk for colorectal cancer: a case-control study in China. Br J Nutr. 2020;123(11):1277-1289.<br>● Shin, A., et al., Dietary intake of calcium, fiber and other micronutrients in relation to colorectal cancer risk: Results from the Shanghai Women's Health Study. International Journal of Cancer, 2006. 119(12): p. 2938-2942.<br>● Liu Y, Li S, Jiang L, Zhang Y, Li Z, Shi J. Solanaceous Vegetables and Colorectal Cancer Risk: A Hospital-Based Matched Case-Control Study in Northeast China. Front Nutr. 2021;8:688897.
Carbohydrate	4	● Yang, G., et al., Environmental and genetic factors in the pathogenesis of colorectal cancer(大肠癌发病的环境因素与遗传因素). Chinese Journal of Epidemiology, 1992. 13(1).<br>● Chiu, B.C.H., et al., Dietary factors and risk of colon cancer in Shanghai, China. Cancer Epidemiology, Biomarkers & Prevention : a Publication of the American Association For Cancer Research, Cosponsored by the American Society of Preventive Oncology, 2003. 12(3): p. 201-208.<br>● Huang, J., et al., Carbohydrate, dietary glycaemic index and glycaemic load, and colorectal cancer risk: a case-control study in China. The British Journal of Nutrition, 2018. 119(8): p. 937-948<br>● Li, H.-L., et al., Dietary glycemic load and risk of colorectal cancer in Chinese women. The American Journal of Clinical Nutrition, 2011. 93(1): p. 101-107.
Protein	5	● lu, R.F., An epidemiological nutrition approach to colon cancer in urban Shanghai(上海市结肠癌营养流行病学调查). Fudan University Journal of Medical Sciences, 1986(5).<br>● Yang, G., et al., Environmental and genetic factors in the pathogenesis of colorectal cancer(大肠癌发病的环境因素与遗传因素). Chinese Journal of Epidemiology, 1992. 13(1).<br>● Lai, K.D., et al., Diet physical activity and colorectal cancer: case control study in Dalian(膳食、体力活动与大肠癌:病例对照研究). Chinese Journal of Prevention and Control of Chronic Diseases, 1995. 3(3).<br>● Whittemore, A.S., et al., Diet, physical activity, and colorectal cancer among Chinese in North America and China. Journal of the National Cancer Institute, 1990. 82(11): p. 915-926.<br>● Chiu, B.C.H., et al., Dietary factors and risk of colon cancer in Shanghai, China. Cancer Epidemiology, Biomarkers & Prevention : a Publication of the American Association For Cancer Research, Cosponsored by the American Society of Preventive Oncology, 2003. 12(3): p. 201-208.
Fiber	16	● lu, R.F., An epidemiological nutrition approach to colon cancer in urban Shanghai(上海市结肠癌营养流行病学调查). Fudan University Journal of Medical Sciences, 1986(5).<br>● Li, L., et al., A Case-Control Study for Colon Cancer in Beijing,China(北京市结肠癌危险因素的病例对照研究). Chinese Journal of Clinical Oncology, 2003(08): p. 26-28.<br>● Peng, X.E., et al., Case-control Study on Risk Factors of Colorectal Cancer in Fujian Province(福建省大肠癌发病危险因素的病例对照研究). Cancer Research on Prevention and Treatment, 2009(9).<br>● Yang, G., et al., Environmental and genetic factors in the pathogenesis of colorectal cancer(大肠癌发病的环境因素与遗传因素). Chinese Journal of Epidemiology, 1992. 13(1).<br>● Lai, K.D., et al., Diet physical activity and colorectal cancer: case control study in Dalian(膳食、体力活动与大肠癌:病例对照研究). Chinese Journal of Prevention and Control of Chronic Diseases, 1995. 3(3).<br>● Xu, G.F., et al., A case-control study of the relationship between resistant starch and colorectal cancer. Acta Nutrimenta Sinica, 2006. 28(1).<br>● Chiu, B.C.H., et al., Dietary factors and risk of colon cancer in Shanghai, China. Cancer Epidemiology, Biomarkers & Prevention : a Publication of the American Association For Cancer Research, Cosponsored by the American Society of Preventive Oncology, 2003. 12(3): p. 201-208.<br>● Che, Q.H. and H.R. Diao, A Study on the Correlation of Dietary Factors with Colorectal Cancer in the Patients from a Hospital of Shenyang(结直肠癌患者膳食相关因素病例对照研究). Chinese Journal of Prevention and Control of Chronic Diseases, 2010. 18(04): p. 368-369.<br>● Chang, C., et al., Relationship between the gene-environment factors to colorectal cancer in the Hakka population in Meizhou area(遗传及环境因素与广东省梅州客家人结直肠癌的关系研究). Chinese Journal of Surgical Oncology, 2018. 10(02): p. 87-91.<br>● Yuan, P., et al., Association of dietary fiber intake with colorectal cancer: a matched casecontrol study(结直肠癌与膳食纤维相关饮食因素病例对照研究). Chinese Journal of Public Health, 2016. 32(12): p. 1719-1723.

		● Shin, A., et al., Dietary intake of calcium, fiber and other micronutrients in relation to colorectal cancer risk: Results from the Shanghai Women's Health Study. International Journal of Cancer, 2006. 119(12): p. 2938-2942.<br>● Yang, G., et al., A Case-control Study on Colorectal Cancer and Dietary Fiber and Calcium of Various Sources(不同来源膳食纤维、钙与结直肠癌关系的研究). CHINESE JOURNAL OF PREVENTIVE MEDICINE, 1994. 28(4): p. 195-198.<br>● Zhong, X., et al., Dietary fiber and fiber fraction intakes and colorectal cancer risk in Chinese adults. Nutrition and Cancer, 2014. 66(3): p. 351-361.<br>● Song, Y., et al., Dietary fibre and the risk of colorectal cancer: a case- control study. Asian Pacific Journal of Cancer Prevention : APJCP, 2015. 16(9): p. 3747-3752.<br>● Huang, J., et al., Carbohydrate, dietary glycaemic index and glycaemic load, and colorectal cancer risk: a case-control study in China. The British Journal of Nutrition, 2018. 119(8): p. 937-948<br>● Liu Y, Li S, Jiang L, Zhang Y, Li Z, Shi J. Solanaceous Vegetables and Colorectal Cancer Risk: A Hospital-Based Matched Case-Control Study in Northeast China. Front Nutr. 2021;8:688897.
Aquatic product	10	● lu, R.F., An epidemiological nutrition approach to colon cancer in urban Shanghai(上海市结肠癌营养流行病学调查). Fudan University Journal of Medical Sciences, 1986(5).<br>● Lee, S.A., et al., Animal origin foods and colorectal cancer risk: a report from the Shanghai Women's Health Study. Nutrition and Cancer, 2009. 61(2): p. 194-205.<br>● Meng, F.H., et al., Investigation on risk factors of colorectal cancer in Guangdong. ACAD J ISI MED COLL PLA, 1994. 14(4).<br>● Song, Y.F., et al., A case control study on the relationship between colorectal cancer and helicobacter pylori Infection and other factors(幽门螺旋杆菌感染等因素与大肠癌关系的病例对照研究). Journal of Modern Oncology, 2007. 15(7).<br>● Murff, H.J., et al., A prospective study of dietary polyunsaturated fatty acids and colorectal cancer risk in Chinese women. Cancer Epidemiology, Biomarkers & Prevention : a Publication of the American Association For Cancer Research, Cosponsored by the American Society of Preventive Oncology, 2009. 18(8): p. 2283-2291.<br>● Chiu, B.C.H., et al., Dietary factors and risk of colon cancer in Shanghai, China. Cancer Epidemiology, Biomarkers & Prevention : a Publication of the American Association For Cancer Research, Cosponsored by the American Society of Preventive Oncology, 2003. 12(3): p. 201-208.<br>● Zhang, Z.Y., et al., A case-control study of 269 cases of colorectal cancer(269 例大肠癌病例对照研究). Chinese Journal of Cancer Prevention and Treatment, 2002. 9(6).<br>● Ye, J.J. and N.F. Shi, A case-control study of risk factors for colorectal cancer in Cixi City, Zhejiang Province(浙江省慈溪市结直肠癌危险因素病例对照研究). Disease surveillance, 2007(03): p. 196-198.<br>● Che, Q.H. and H.R. Diao, A Study on the Correlation of Dietary Factors with Colorectal Cancer in the Patients from a Hospital of Shenyang(结直肠癌患者膳食相关因素病例对照研究). Chinese Journal of Prevention and Control of Chronic Diseases, 2010. 18(04): p. 368-369.<br>● Xu, M., et al., Higher freshwater fish and sea fish intake is inversely associated with colorectal cancer risk among Chinese population: a case-control study. Scientific Reports, 2015. 5: p. 12976.
SFA	3	● Lee, S.A., et al., Animal origin foods and colorectal cancer risk: a report from the Shanghai Women's Health Study. Nutrition and Cancer, 2009. 61(2): p. 194-205.<br>● Lai, K.D., et al., Diet physical activity and colorectal cancer: case control study in Dalian(膳食、体力活动与大肠癌:病例对照研究). Chinese Journal of Prevention and Control of Chronic Diseases, 1995. 3(3).<br>● Zhong, X., et al., Dietary fat, fatty acid intakes and colorectal cancer risk in Chinese adults: a case-control study. European Journal of Cancer Prevention : the Official Journal of the European Cancer Prevention Organisation (ECP), 2013. 22(5): p. 438-447.
UFA	3	● Lee, S.A., et al., Animal origin foods and colorectal cancer risk: a report from the Shanghai Women's Health Study. Nutrition and Cancer, 2009. 61(2): p. 194-205.<br>● Zhong, X., et al., Dietary fat, fatty acid intakes and colorectal cancer risk in Chinese adults: a case-control study. European Journal of Cancer Prevention : the Official Journal of the European Cancer Prevention Organisation (ECP), 2013. 22(5): p. 438-447.<br>● Murff, H.J., et al., A prospective study of dietary polyunsaturated fatty acids and colorectal cancer risk in Chinese women. Cancer Epidemiology, Biomarkers & Prevention : a Publication of the American Association For Cancer Research, Cosponsored by the American Society of Preventive Oncology, 2009. 18(8): p. 2283-2291.
Beans and soy products	5	● lu, R.F., An epidemiological nutrition approach to colon cancer in urban Shanghai(上海市结肠癌营养流行病学调查). Fudan University Journal of Medical Sciences, 1986(5).<br>● Li, L., et al., A Case-Control Study for Colon Cancer in Beijing,China(北京市结肠癌危险因素的病例对照研究). Chinese Journal of Clinical Oncology, 2003(08): p. 26-28.<br>● Chiu, B.C.H., et al., Dietary factors and risk of colon cancer in Shanghai, China. Cancer Epidemiology, Biomarkers & Prevention : a Publication of the American Association For

		Cancer Research, Cosponsored by the American Society of Preventive Oncology, 2003. 12(3): p. 201-208.<br>● Yang, G., et al., Prospective cohort study of soy food intake and colorectal cancer risk in women. The American Journal of Clinical Nutrition, 2009. 89(2): p. 577-583.<br>● Vogtmann, E., et al., Fruit and vegetable intake and the risk of colorectal cancer: results from the Shanghai Men's Health Study. CanceVogtmann, E., et al., Fruit and vegetable intake and the risk of colorectal cancer: results from the Shanghai Men's Health Study. Cancer Causes & Control : CCC, 2013. 24(11): p. 1935-1945.
Fried food	6	● Ding, J.H., et al., A case-control study of large bowel cancer(大肠癌病例对照研究). Journal of Practical Oncology, 1992(03): p. 176-178.<br>● Xiong, J.Y., D.J. Shen, and F.Y. Zhu, Epidemological Study on Hihg Risk Factos for Large Intestine Cancer in Loudi City(娄底市大肠癌高危因素临床流行病学研究). Journal of Chinese Physician, 2002. 4(4).<br>● Ma, J.J., et al., Study on the incidence and the influencing factors of colorectal cancer in Hanting District of Weifang(潍坊市寒亭区结直肠癌发病情况及影响因素研究). Chinese Journal of Hospital Statistics, 2020. 27(04): p. 349-353.<br>● Wu, R.L.Q., et al., Case-control study of diet-related risk factors for colorectal cancer(结直肠癌患病饮食相关危险因素病例对照研究). Journal of Diseases Monitor & Control, 2016. 10(01): p. 70-72.<br>● Chen, K., et al., Relationship between glutathione transferase gene polymorphism, dietary exposure and colorectal cancer(谷胱甘肽转移酶基因多态、饮食暴露与结直肠癌关系的研究). Chinese Journal of Digestion, 2004. 24(6): p. 377-379.<br>● Wang W, Dong Z, Zhang X, Li W, Li P, Chen X. Dietary and the Risk of Sporadic Colorectal Cancer in China: A Case-control Study. Iran J Public Health. 2018;47(9):1327-1335.
Smoked products	6	● Lee, S.A., et al., Animal origin foods and colorectal cancer risk: a report from the Shanghai Women's Health Study. Nutrition and Cancer, 2009. 61(2): p. 194-205.<br>● Meng, F.H., et al., Investigation on risk factors of colorectal cancer in Guangdong. ACAD J ISI MED COLL PLA, 1994. 14(4).<br>● Ma, J.J., et al., Study on the incidence and the influencing factors of colorectal cancer in Hanting District of Weifang(潍坊市寒亭区结直肠癌发病情况及影响因素研究). Chinese Journal of Hospital Statistics, 2020. 27(04): p. 349-353.<br>● Wang, X.H., et al., Colon cancer risk factors in Jiashan county, Zhejiang province, the highest incidence area in China(大肠癌高发区居民结肠癌危险因素研究). Chinese Journal of Oncology, 2001(06): p. 44-46.<br>● Chang, C., et al., Relationship between the gene-environment factors to colorectal cancer in the Hakka population in Meizhou area(遗传及环境因素与广东省梅州客家人结直肠癌的关系研究). Chinese Journal of Surgical Oncology, 2018. 10(02): p. 87-91.<br>● Chen, K., et al., Relationship between glutathione transferase gene polymorphism, dietary exposure and colorectal cancer(谷胱甘肽转移酶基因多态、饮食暴露与结直肠癌关系的研究). Chinese Journal of Digestion, 2004. 24(6): p. 377-379.
Salted food	13	● Lee, S.A., et al., Animal origin foods and colorectal cancer risk: a report from the Shanghai Women's Health Study. Nutrition and Cancer, 2009. 61(2): p. 194-205.<br>● Keskin, H., et al., Colorectal cancer in the Linxian China Nutrition Intervention Trial: Risk factors and intervention results. PloS One, 2021. 16(9): p. e0255322.<br>● Wu, D.R., A case-control study on risk factors of colorectal cancer in Jiashan County(嘉善县大肠癌危险因素的病例对照研究). Journal of Practical Oncology, 1990. 5(2).<br>● Meng, F.H., et al., Investigation on risk factors of colorectal cancer in Guangdong. ACAD J ISI MED COLL PLA, 1994. 14(4).<br>● Xiong, J.Y., D.J. Shen, and F.Y. Zhu, Epidemological Study on Hihg Risk Factos for Large Intestine Cancer in Loudi City(娄底市大肠癌高危因素临床流行病学研究). Journal of Chinese Physician, 2002. 4(4).<br>● Ma, J.J., et al., Study on the incidence and the influencing factors of colorectal cancer in Hanting District of Weifang(潍坊市寒亭区结直肠癌发病情况及影响因素研究). Chinese Journal of Hospital Statistics, 2020. 27(04): p. 349-353.<br>● Chiu, B.C.H., et al., Dietary factors and risk of colon cancer in Shanghai, China. Cancer Epidemiology, Biomarkers & Prevention : a Publication of the American Association For Cancer Research, Cosponsored by the American Society of Preventive Oncology, 2003. 12(3): p. 201-208.<br>● Che, Q.H. and H.R. Diao, A Study on the Correlation of Dietary Factors with Colorectal Cancer in the Patients from a Hospital of Shenyang(结直肠癌患者膳食相关因素病例对照研究). Chinese Journal of Prevention and Control of Chronic Diseases, 2010. 18(04): p. 368-369.<br>● Wu, R.L.Q., et al., Case-control study of diet-related risk factors for colorectal cancer(结直肠癌患病饮食相关危险因素病例对照研究). Journal of Diseases Monitor & Control, 2016. 10(01): p. 70-72.

		• Qin, M., et al., Risk factors for colorectal neoplasms based on colonoscopy and pathological diagnoses of Chinese citizens: a multicenter, case-control study. International Journal of Colorectal Disease, 2015. 30(3): p. 353-361.<br>• Xu, M., et al., Higher freshwater fish and sea fish intake is inversely associated with colorectal cancer risk among Chinese population: a case-control study. Scientific Reports, 2015. 5: p. 12976.<br>• Chen, K., et al., Relationship between glutathione transferase gene polymorphism, dietary exposure and colorectal cancer(谷胱甘肽转移酶基因多态、饮食暴露与结直肠癌关系的研究). Chinese Journal of Digestion, 2004. 24(6): p. 377-379.<br>• Cao.L.N., et al., Relationship between Dietary Patterns and Colorectal Cancer: A Case-control Study(膳食模式与结直肠癌相关性的病例对照研究). Journal of Nursing(China), 2016. 23(13): p. 1-5.
Roast food	3	• Wang W, Dong Z, Zhang X, Li W, Li P, Chen X. Dietary and the Risk of Sporadic Colorectal Cancer in China: A Case-control Study. Iran J Public Health. 2018;47(9):1327-1335.<br>• Ho, J., S. Yuen, and S. Yuen, A case-control study on environmental and familial risk factors for colorectal cancer in Hong Kong: physical activity reduces colorectal cancer risk. 2006.<br>• Chen, J., H.G. Zhu, and L.Q. Xia, A case-control study of dietary risk factors for colon cancer(结肠癌饮食危险因素病例对照研究). Chinese Journal of Public Health, 2010. 26(04): p. 441.
Cholesterol	3	• lu, R.F., An epidemiological nutrition approach to colon cancer in urban Shanghai(上海市结肠癌营养流行病学调查). Fudan University Journal of Medical Sciences, 1986(5).<br>• Lee, S.A., et al., Animal origin foods and colorectal cancer risk: a report from the Shanghai Women's Health Study. Nutrition and Cancer, 2009. 61(2): p. 194-205.<br>• Xu, G.F., et al., A case-control study of the relationship between resistant starch and colorectal cancer. Acta Nutrimenta Sinica, 2006. 28(1).
Total energy	4	• lu, R.F., An epidemiological nutrition approach to colon cancer in urban Shanghai(上海市结肠癌营养流行病学调查). Fudan University Journal of Medical Sciences, 1986(5).<br>• Lee, S.A., et al., Animal origin foods and colorectal cancer risk: a report from the Shanghai Women's Health Study. Nutrition and Cancer, 2009. 61(2): p. 194-205.<br>• Xu, G.F., et al., A case-control study of the relationship between resistant starch and colorectal cancer. Acta Nutrimenta Sinica, 2006. 28(1).<br>• Chiu, B.C.H., et al., Dietary factors and risk of colon cancer in Shanghai, China. Cancer Epidemiology, Biomarkers & Prevention : a Publication of the American Association For Cancer Research, Cosponsored by the American Society of Preventive Oncology, 2003. 12(3): p. 201-208.
Total fat	3	• lu, R.F., An epidemiological nutrition approach to colon cancer in urban Shanghai(上海市结肠癌营养流行病学调查). Fudan University Journal of Medical Sciences, 1986(5).<br>• Yang, G., et al., Environmental and genetic factors in the pathogenesis of colorectal cancer(大肠癌发病的环境因素与遗传因素). Chinese Journal of Epidemiology, 1992. 13(1).<br>• Chiu, B.C.H., et al., Dietary factors and risk of colon cancer in Shanghai, China. Cancer Epidemiology, Biomarkers & Prevention : a Publication of the American Association For Cancer Research, Cosponsored by the American Society of Preventive Oncology, 2003. 12(3): p. 201-208.
Oil type (animal oils)	7	• Zhao, J.S., et al., A Case-control Study on Risk Factors of Colonic Cancer(结肠癌危险因素的病例对照研究). Cancer Research on Prevention and Treatment, 2008(07): p. 524-526.<br>• Jiang, Q.T., et al., The case-control study on relationship between environmental risk exposure and incidence of colorectal cancer in the population-based cohort(随访队列的结直肠癌危险因素的病例-对照研究). Tumor, 2004(01): p. 6-10.<br>• Wang, X.H., et al., Colon cancer risk factors in Jiashan county, Zhejiang province, the highest incidence area in China(大肠癌高发区居民结肠癌危险因素研究). Chinese Journal of Oncology, 2001(06): p. 44-46.<br>• Zhu, L.P., et al., A Case-control Study of Risk Factors for Colorectal Cancer in Jiangxi Province(江西省 308 例大肠癌危险因素的病例对照研究). Chinese Journal of Prevention and Control of Chronic Non-communicable Diseases, 2001. 9(3).<br>• Liu, Y.T., J.H. Ding, and Y.G. Lin, A case-control study of rectal cancer(直肠癌病例对照研究). Jiangsu Medical Journal, 1993(04): p. 218.<br>• Liu, A.Z., et al., 153 Paired case control study of risk factors for colorectal cancer(153 对大肠癌危险因素的配对病例对照研究). CHINA PUBLIC HEALTH, 1997. 13(4).<br>• Wu, R.L.Q., et al., Case-control study of diet-related risk factors for colorectal cancer(结直肠癌患病饮食相关危险因素病例对照研究). Journal of Diseases Monitor & Control, 2016. 10(01): p. 70-72.
Greasy food	12	• Wu, Y., et al., An Analysis on Risk Factors of Common Cancers in Zhejiang Province. China Cancer(浙江省常见恶性肿瘤危险因素分析), 2012. 21(04): p. 258-263.<br>• Feng, Y.J., A paired case-control study of risk factors for rectal cancer(直肠癌危险因素的配对病例对照研究). Journal of Chinese Physician, 2008(12): p. 1690-1691.

		● Peng, X.E., et al., Case-control Study on Risk Factors of Colorectal Cancer in Fujian Province(福建省大肠癌发病危险因素的病例对照研究). Cancer Research on Prevention and Treatment, 2009(9).<br>● Ma, J.J., et al., Study on the incidence and the influencing factors of colorectal cancer in Hanting District of Weifang(潍坊市寒亭区结直肠癌发病情况及影响因素研究). Chinese Journal of Hospital Statistics, 2020. 27(04): p. 349-353.<br>● Liu, Y.T., J.H. Ding, and Y.G. Lin, A case-control study of rectal cancer(直肠癌病例对照研究). Jiangsu Medical Journal, 1993(04): p. 218.<br>● Ye, J.J. and N.F. Shi, A case-control study of risk factors for colorectal cancer in Cixi City, Zhejiang Province(浙江省慈溪市结直肠癌危险因素病例对照研究). Disease surveillance, 2007(03): p. 196-198.<br>● Che, Q.H. and H.R. Diao, A Study on the Correlation of Dietary Factors with Colorectal Cancer in the Patients from a Hospital of Shenyang(结直肠癌患者膳食相关因素病例对照研究). Chinese Journal of Prevention and Control of Chronic Diseases, 2010. 18(04): p. 368-369.<br>● Chen, J., H.G. Zhu, and L.Q. Xia, A case-control study of dietary risk factors for colon cancer(结肠癌饮食危险因素病例对照研究). Chinese Journal of Public Health, 2010. 26(04): p. 441.<br>● Zhang, X.H. and A.G. Xu, Analysis on the Risk Factors for Colorectal Cancer(大肠癌危险因素的病例对照研究). Chinese Journal of Clinical Gastroenterology, 2010(5).<br>● Li, X.L., A case-control family study of colorectal cancer in rural area of Taizhou City in Jiangsu Province(江苏省泰兴市农村地区结直肠癌危险因素的病例对照家系研究). International Journal of Pathology and Clinical Medicine, 2013. 33(02): p. 112-115.<br>● Gao, W.X., et al., Smoking and other related factors and epidemiology of colorectal cancer(吸烟等相关因素与大肠癌的流行病学研究). China Tropical Medicine, 2017. 17(7).<br>● Chang, C., et al., Relationship between the gene-environment factors to colorectal cancer in the Hakka population in Meizhou area(遗传及环境因素与广东省梅州客家人结直肠癌的关系研究). Chinese Journal of Surgical Oncology, 2018. 10(02): p. 87-91.
DII	3	● Tang, D., et al., Research of genetic and lifestyle risk score on risk assessment of colorectal cancer(遗传和生活方式风险评分与结直肠癌发生风险的评估研究). Journal of Medical Forum, 2021. 42(19): p. 97-101.<br>● Wu, G.H. and H. Ren, In flammatory potential of diet and risks of colorectal cancer:a case-control study(病例对照研究分析膳食与炎症危险因素与结直肠癌的关系). Foreign Medical Sciences(Section of Medgeography), 2016. 37(03): p. 243-246.<br>● Abulimiti, A., et al., The Dietary Inflammatory Index Is Positively Associated with Colorectal Cancer Risk in a Chinese Case-Control Study. Nutrients, 2020. 12(1).
Well water	3	● Jiang, Q.T., et al., The case-control study on relationship between environmental risk exposure and incidence of colorectal cancer in the population-based cohort(随访队列的结直肠癌危险因素的病例-对照研究). Tumor, 2004(01): p. 6-10.<br>● Chen, K., et al., Association of Drinking Water Source and Colorectal Cancer Incidence: A Prospect Cohort Study(饮水类型与结直肠癌发病率关系的前瞻性队列研究). Chinese Journal of Cancer, 2004(05): p. 550-554.<br>● Chen, K., Association of Drinking Water and Colorectal Cance(饮水类型与大肠癌发病率关系的研究). Chinese Journal of Public Health, 1991. 10(6).
**Mental profile**		
History of mental stimulation	5	● Wu, Y., et al., An Analysis on Risk Factors of Common Cancers in Zhejiang Province. China Cancer(浙江省常见恶性肿瘤危险因素分析), 2012. 21(04): p. 258-263.<br>● Wu, D.R., A case-control study on risk factors of colorectal cancer in Jiashan County(嘉善县大肠癌危险因素的病例对照研究). Journal of Practical Oncology, 1990. 5(2).<br>● Ma, J.J., et al., Study on the incidence and the influencing factors of colorectal cancer in Hanting District of Weifang(潍坊市寒亭区结直肠癌发病情况及影响因素研究). Chinese Journal of Hospital Statistics, 2020. 27(04): p. 349-353.<br>● Li, W., et al., Case-control study on risk factors for colorectal cancer in Shandong pronvince(山东省大肠癌影响因素病例对照研究). Chinese Journal of Public Health, 2007(08): p. 904-905.<br>● He, H.Q., K. Chen, and X.Y. Ma, History of psychological stress and colorectal cancer:a population-based prospective cohort study(精神刺激史与结直肠癌发病关系的队列研究). Tumor, 2006(06): p. 537-539.
Negative emotion	5	● Wu, Y., et al., An Analysis on Risk Factors of Common Cancers in Zhejiang Province. China Cancer(浙江省常见恶性肿瘤危险因素分析), 2012. 21(04): p. 258-263.<br>● Wang, X.H., et al., A case-control study of risk factors for colon cancer(结肠癌危险因素的病例对照研究). Chinese Journal of Cancer, 2001(09): p. 977-980.<br>● Wang, X.H., et al., Colon cancer risk factors in Jiashan county, Zhejiang province, the highest incidence area in China(大肠癌高发区居民结肠癌危险因素研究). Chinese Journal of Oncology, 2001(06): p. 44-46.

		● Li, D.D., et al., Correlation between psychosocial risk factors and colorectal cancer9 心理社会危险因素与大肠癌的相关性研究). Practical Oncology Journal, 2012. 26(4).<br>● Wang W, Dong Z, Zhang X, Li W, Li P, Chen X. Dietary and the Risk of Sporadic Colorectal Cancer in China: A Case-control Study. Iran J Public Health. 2018;47(9):1327-1335.
Bad mental state	4	● Zhang, X.Y., et al., An Analysis on Risk Factors of Common Malignant Tumors in Tongxiang City(上海市结肠癌营养流行病学调查). Zhejiang Journal of Preventive Medicine, 2014. 26(02): p. 150-153.<br>● Wu, D.R., A case-control study on risk factors of colorectal cancer in Jiashan County(嘉善县大肠癌危险因素的病例对照研究). Journal of Practical Oncology, 1990. 5(2).<br>● Zhang, C., R.T. Wang, and T.G. Wang, A case-control study of 250 cases of colorectal cancer in Beijing(北京市 250 例大肠癌的病例对照研究). Chinese Journal of Epidemiology, 1992. 13(6).<br>● Wang, X.H., et al., Colon cancer risk factors in Jiashan county, Zhejiang province, the highest incidence area in China(大肠癌高发区居民结肠癌危险因素研究). Chinese Journal of Oncology, 2001(06): p. 44-46.
Poor emotion management	3	● Nie, S.F., et al., A Case-control Study on the Risk Factors for Colorectal Cancer among Urban Inhabitants in Wuhan of China(武汉市大肠癌危险因素的病例对照研究). Chinese Journal of Prevention and Control of Chronic Non-communicable Diseases, 2002. 10(6).<br>● Wang, F.J., L.F. Jiang, and J.F. Guo, A case-control study on the risk factors for colorectal cancer in Xiaoshan county(萧山区大肠癌发病危险因素的病例对照研究). Chinese Rural Health Service Administration, 2012. 32(9).<br>● Ye, J.J. and N.F. Shi, A case-control study of risk factors for colorectal cancer in Cixi City, Zhejiang Province(浙江省慈溪市结直肠癌危险因素病例对照研究). Disease surveillance, 2007(03): p. 196-198.
Type A personality	4	● lu, R.F., An epidemiological nutrition approach to colon cancer in urban Shanghai(上海市结肠癌营养流行病学调查). Fudan University Journal of Medical Sciences, 1986(5).<br>● Li, L., et al., A Case-Control Study for Colon Cancer in Beijing,China(北京市结肠癌危险因素的病例对照研究). Chinese Journal of Clinical Oncology, 2003(08): p. 26-28.<br>● Wu, Y., et al., An Analysis on Risk Factors of Common Cancers in Zhejiang Province. China Cancer(浙江省常见恶性肿瘤危险因素分析), 2012. 21(04): p. 258-263.<br>● Zhang, X.Y., et al., An Analysis on Risk Factors of Common Malignant Tumors in Tongxiang City(上海市结肠癌营养流行病学调查). Zhejiang Journal of Preventive Medicine, 2014. 26(02): p. 150-153.
**Personal history**		
Helicobacter pylori infection	3	● Dong, Y.F., et al., Correlations between gastric Helicobacter pylori infection and colorectal polyps or cancer(幽门螺杆菌感染与结直肠息肉及结直肠癌的相关性分析). Chinese Journal of Internal Medicine, 2019(02): p. 139-142.<br>● Liu, I.L., et al., Helicobacter pylori infection and the risk of colorectal cancer: a nationwide population-based cohort study. QJM : Monthly Journal of the Association of Physicians, 2019. 112(10): p. 787-792.<br>● Wang, M., et al., Association of Helicobacter pylori infection with colorectal polyps and malignancy in China. World Journal of Gastrointestinal Oncology, 2020. 12(5): p. 582-591.
History of hepatobiliary disease	14	● Wu, Y., et al., An Analysis on Risk Factors of Common Cancers in Zhejiang Province. China Cancer(浙江省常见恶性肿瘤危险因素分析), 2012. 21(04): p. 258-263.<br>● Yao, J.H., Relationship between body mass index and the colorectal cancer(身体体质指数与结直肠癌的相关性研究). Chinese Journal of Gastroenterology and Hepatology, 2011. 20(10): p. 904-906.<br>● Wang, Z., et al., Associations Between Nonalcoholic Fatty Liver Disease and Cancers in a Large Cohort in China. Clinical Gastroenterology and Hepatology : the Official Clinical Practice Journal of the American Gastroenterological Association, 2021. 19(4).<br>● Wu, D.R., A case-control study on risk factors of colorectal cancer in Jiashan County(嘉善县大肠癌危险因素的病例对照研究). Journal of Practical Oncology, 1990. 5(2).<br>● Zhao, J.S., et al., A Case-control Study on Risk Factors of Colonic Cancer(结肠癌危险因素的病例对照研究). Cancer Research on Prevention and Treatment, 2008(07): p. 524-526.<br>● Jiang, Q.T., et al., The case-control study on relationship between environmental risk exposure and incidence of colorectal cancer in the population-based cohort(随访队列的结直肠癌危险因素的病例-对照研究). Tumor, 2004(01): p. 6-10.<br>● Zeng, Z.S. and Z.F. Zhang, Cholecystectomy and colorectal cancer in China. Surgical Oncology, 1993. 2(6): p. 311-319.<br>● Yang, G., et al.,Some diseases of digestive system and large bowel cancer. A population-based case-control study in Shanghai(消化道疾患与大肠癌关系的病例:对照研究). Tumor, 1996. 16(2).<br>● Chen, M.T., Study on the Relationship Between Colorectal Cancer and Cholecystectomy(大肠癌发生率与胆囊切除关系研究). Journal of Chinese Physician, 2003(03): p. 313-314.

		• Song, C., et al., Associations Between Hepatitis B Virus Infection and Risk of All Cancer Types. JAMA Network Open, 2019. 2(6): p. e195718.<br>• Liu, T., et al., Associations between hepatitis B virus infection and risk of colorectal Cancer: a population-based prospective study. BMC Cancer, 2021. 21(1): p. 1119.<br>• Pang, Y., et al., Causal effects of gallstone disease on risk of gastrointestinal cancer in Chinese. British Journal of Cancer, 2021. 124(11): p. 1864-1872.<br>• Liu T, Song C, Zhang Y, et al. Hepatitis B virus infection and the risk of gastrointestinal cancers among Chinese population: A prospective cohort study. Int J Cancer. 2022;150(6):1018-1028.<br>• Lin XF, Shi KQ, You J, et al. Increased risk of colorectal malignant neoplasm in patients with nonalcoholic fatty liver disease: a large study. Mol Biol Rep. 2014;41(5):2989-2997.
Family history of cancer	33	• lu, R.F., An epidemiological nutrition approach to colon cancer in urban Shanghai(上海市结肠癌营养流行病学调查). Fudan University Journal of Medical Sciences, 1986(5).<br>• Zhang, X.Y., et al., An Analysis on Risk Factors of Common Malignant Tumors in Tongxiang City(上海市结肠癌营养流行病学调查). Zhejiang Journal of Preventive Medicine, 2014. 26(02): p. 150-153.<br>• Lee, S.A., et al., Animal origin foods and colorectal cancer risk: a report from the Shanghai Women's Health Study. Nutrition and Cancer, 2009. 61(2): p. 194-205.<br>• Wei, Y.S., et al., Risk factors for sporadic colorectal cancer in southern Chinese. World Journal of Gastroenterology, 2009. 15(20): p. 2526-2530.<br>• Wu, D.R., A case-control study on risk factors of colorectal cancer in Jiashan County(嘉善县大肠癌危险因素的病例对照研究). Journal of Practical Oncology, 1990. 5(2).<br>• Ding, J.H., et al., A case-control study of large bowel cancer(大肠癌病例对照研究). Journal of Practical Oncology, 1992(03): p. 176-178.<br>• Zhang, C., R.T. Wang, and T.G. Wang, A case-control study of 250 cases of colorectal cancer in Beijing(北京市 250 例大肠癌的病例对照研究). Chinese Journal of Epidemiology, 1992. 13(6).<br>• Meng, F.H., et al., Investigation on risk factors of colorectal cancer in Guangdong. ACAD J ISI MED COLL PLA, 1994. 14(4).<br>• Nie, S.F., et al., A Case-control Study on the Risk Factors for Colorectal Cancer among Urban Inhabitants in Wuhan of China(武汉市大肠癌危险因素的病例对照研究). Chinese Journal of Prevention and Control of Chronic Non-communicable Diseases, 2002. 10(6).<br>• Xiong, J.Y., D.J. Shen, and F.Y. Zhu, Epidemological Study on Hihg Risk Factos for Large Intestine Cancer in Loudi City(娄底市大肠癌高危因素临床流行病学研究). Journal of Chinese Physician, 2002. 4(4).<br>• Song, Y.F., et al., A case control study on the relationship between colorectal cancer and helicobacter pylori Infection and other factors(幽门螺旋杆菌感染等因素与大肠癌关系的病例对照研究). Journal of Modern Oncology, 2007. 15(7).<br>• Feng, Y.J., A paired case-control study of risk factors for rectal cancer(直肠癌危险因素的配对病例对照研究). Journal of Chinese Physician, 2008(12): p. 1690-1691.<br>• Zhao, J.S., et al., A Case-control Study on Risk Factors of Colonic Cancer(结肠癌危险因素的病例对照研究). Cancer Research on Prevention and Treatment, 2008(07): p. 524-526.<br>• Murphy, G., et al., Family cancer history affecting risk of colorectal cancer in a prospective cohort of Chinese women. Cancer Causes & Control : CCC, 2009. 20(8): p. 1517-1521.<br>• Peng, X.E., et al., Case-control Study on Risk Factors of Colorectal Cancer in Fujian Province(福建省大肠癌发病危险因素的病例对照研究). Cancer Research on Prevention and Treatment, 2009(9).<br>• Li, X.F., Q.Y. An, and X.H. Gao,1:1 matched case-control study on environmental risk factors of rectal cancer in rural areas(城市直肠癌环境危险因素的 1:1 匹配病例对照研究). Modern Preventive Medicine, 2010. 37(18): p. 3413-3414.<br>• Wang, F.J., L.F. Jiang, and J.F. Guo, A case-control study on the risk factors for colorectal cancer in Xiaoshan county(萧山区大肠癌发病危险因素的病例对照研究). Chinese Rural Health Service Administration, 2012. 32(9).<br>• Ma, J.J., et al., Study on the incidence and the influencing factors of colorectal cancer in Hanting District of Weifang(潍坊市寒亭区结直肠癌发病情况及影响因素研究). Chinese Journal of Hospital Statistics, 2020. 27(04): p. 349-353.<br>• Zhang, T., et al., Analysis of the related factors between lifestyle and colorectal cancer in Nanyang area(南阳地区居民生活方式与结直肠癌相关因素分析). Journal of Community Medicine, 2020. 18(18): p. 1255-1258.<br>• Yang, G., et al., Dietary Factors and Cancer of the Colonand Rectum in a Population based Case-control Study in Shanghai(结、直肠癌与营养因素的流行病学研究). Chinese Journal of Epidemiology, 1994. 15(5): p. 299-303.<br>• Yang, G., et al., Environmental and genetic factors in the pathogenesis of colorectal cancer(大肠癌发病的环境因素与遗传因素). Chinese Journal of Epidemiology, 1992. 13(1).<br>• Guo, Z.R., Schistosomiasis japonicum and colorectal cancer :Logistic regression model to explore the etiology of colorectal cancer(日本血吸虫病与大肠癌:Logistic 回归模型探讨大肠癌的病因). Chinese Journal of Epidemiology, 1987(1).

		● Li, H.Y., J.B. Wang, and S.W. Ji, Risk analysis on diabetes mellitus and colorectal cancer(糖尿病与结直肠癌发生的危险性分析). Journal of Jilin University(Medicine Edition), 2007(02): p. 396-398.<br>● Hu, S.Q., Z. Tang, and M. Zhang, Investigation on the risks of colorectal cancer in patients with diabetes mellitus(糖尿病与结直肠癌患病危险关系的调查分析). World Chinese Journal of Digestology, 2007(01): p. 88-91.<br>● Jiang, Q.T., et al., The case-control study on relationship between environmental risk exposure and incidence of colorectal cancer in the population-based cohort(随访队列的结直肠癌危险因素的病例-对照研究). Tumor, 2004(01): p. 6-10.<br>● Li, W., et al., Case-control study on risk factors for colorectal cancer in Shandong pronvince(山东省大肠癌影响因素病例对照研究). Chinese Journal of Public Health, 2007(08): p. 904-905.<br>● Wang, X.H., et al., A case-control study of risk factors for colon cancer(结肠癌危险因素的病例对照研究). Chinese Journal of Cancer, 2001(09): p. 977-980.<br>● Wang, X.H., et al., Colon cancer risk factors in Jiashan county, Zhejiang province, the highest incidence area in China(大肠癌高发区居民结肠癌危险因素研究). Chinese Journal of Oncology, 2001(06): p. 44-46.<br>● Yu, Z.G., et al., The Study on the Colorectal Cancer Related Risk Factors of 577 Cases(577 例大肠癌相关危险因素的研究). Chinese Journal of Clinical Gastroenterology, 2013. 25(02): p. 85-87.<br>● Yang, T., et al., Intake of Pickled Vegetables and Colorectal Cancer(腌泡菜摄入及其与其他饮食习惯的交互作用与结直肠癌发生风险的关系). Journal of Sichuan University(Medical Science Edition), 2017. 48(06): p. 886-890.<br>● Nie, H.Y. and D.H. Zhu, Analysis of the risk factors of patients with coprectal cancer(糖尿病与结直肠癌发生的危险性分析). Laboratory Medicine and Clinic, 2018. 15(07): p. 915-917+920.<br>● Keskin, H., et al., Colorectal cancer in the Linxian China Nutrition Intervention Trial: Risk factors and intervention results. PloS One, 2021. 16(9): p. e0255322.<br>● Lin Y, Peng Y, Liang B, et al. Associations of dinner-to-bed time, post-dinner walk and sleep duration with colorectal cancer: A case-control study. Medicine (Baltimore). 2018;97(34):e12038.
Family history of CRC	15	● lu, R.F., An epidemiological nutrition approach to colon cancer in urban Shanghai(上海市结肠癌营养流行病学调查). Fudan University Journal of Medical Sciences, 1986(5).<br>● Lee, S.A., et al., Animal origin foods and colorectal cancer risk: a report from the Shanghai Women's Health Study. Nutrition and Cancer, 2009. 61(2): p. 194-205.<br>● Murphy, G., et al., Family cancer history affecting risk of colorectal cancer in a prospective cohort of Chinese women. Cancer Causes & Control : CCC, 2009. 20(8): p. 1517-1521.<br>● Yang, G., et al., Dietary Factors and Cancer of the Colonand Rectum in a Population based Case-control Study in Shanghai(结、直肠癌与营养因素的流行病学研究). Chinese Journal of Epidemiology, 1994. 15(5): p. 299-303.<br>● Yang, G., et al., Environmental and genetic factors in the pathogenesis of colorectal cancer(大肠癌发病的环境因素与遗传因素). Chinese Journal of Epidemiology, 1992. 13(1).<br>● Guo, Z.R., Schistosomiasis japonicum and colorectal cancer :Logistic regression model to explore the etiology of colorectal cancer(日本血吸虫病与大肠癌:Logistic 回归模型探讨大肠癌的病因). Chinese Journal of Epidemiology, 1987(1).<br>● Li, H.Y., J.B. Wang, and S.W. Ji, Risk analysis on diabetes mellitus and colorectal cancer(糖尿病与结直肠癌发生的危险性分析). Journal of Jilin University(Medicine Edition), 2007(02): p. 396-398.<br>● Hu, S.Q., Z. Tang, and M. Zhang, Investigation on the risks of colorectal cancer in patients with diabetes mellitus(糖尿病与结直肠癌患病危险关系的调查分析). World Chinese Journal of Digestology, 2007(01): p. 88-91.<br>● Jiang, Q.T., et al., The case-control study on relationship between environmental risk exposure and incidence of colorectal cancer in the population-based cohort(随访队列的结直肠癌危险因素的病例-对照研究). Tumor, 2004(01): p. 6-10.<br>● Li, W., et al., Case-control study on risk factors for colorectal cancer in Shandong pronvince(山东省大肠癌影响因素病例对照研究). Chinese Journal of Public Health, 2007(08): p. 904-905.<br>● Wang, X.H., et al., A case-control study of risk factors for colon cancer(结肠癌危险因素的病例对照研究). Chinese Journal of Cancer, 2001(09): p. 977-980.<br>● Wang, X.H., et al., Colon cancer risk factors in Jiashan county, Zhejiang province, the highest incidence area in China(大肠癌高发区居民结肠癌危险因素研究). Chinese Journal of Oncology, 2001(06): p. 44-46.<br>● Yu, Z.G., et al., The Study on the Colorectal Cancer Related Risk Factors of 577 Cases(577 例大肠癌相关危险因素的研究). Chinese Journal of Clinical Gastroenterology, 2013. 25(02): p. 85-87.

		● Yang, T., et al., Intake of Pickled Vegetables and Colorectal Cancer(腌泡菜摄入及其与其他饮食习惯的交互作用与结直肠癌发生风险的关系). Journal of Sichuan University(Medical Science Edition), 2017. 48(06): p. 886-890.<br>● Nie, H.Y. and D.H. Zhu, Analysis of the risk factors of patients with coprectal cancer(糖尿病与结直肠癌发生的危险性分析). Laboratory Medicine and Clinic, 2018. 15(07): p. 915-917+920.
Diabetes	11	● Yao, J.H., Relationship between body mass index and the colorectal cancer(身体体质指数与结直肠癌的相关性研究). Chinese Journal of Gastroenterology and Hepatology, 2011. 20(10): p. 904-906.<br>● Zhang, D.W., et al., Relationship of patients with type 2 diabetes mellitus with colorectal cancer(II型糖尿病与结直肠癌的相关性研究). Journal of Modern Oncology, 2011. 19(09): p. 1802-1805.<br>● Wang, Z., et al., Associations Between Nonalcoholic Fatty Liver Disease and Cancers in a Large Cohort in China. Clinical Gastroenterology and Hepatology : the Official Clinical Practice Journal of the American Gastroenterological Association, 2021. 19(4).<br>● Ma, J.J., et al., Study on the incidence and the influencing factors of colorectal cancer in Hanting District of Weifang(潍坊市寒亭区结直肠癌发病情况及影响因素研究). Chinese Journal of Hospital Statistics, 2020. 27(04): p. 349-353.<br>● Li, H.Y., J.B. Wang, and S.W. Ji, Risk analysis on diabetes mellitus and colorectal cancer(糖尿病与结直肠癌发生的危险性分析). Journal of Jilin University(Medicine Edition), 2007(02): p. 396-398.<br>● Hu, S.Q., Z. Tang, and M. Zhang, Investigation on the risks of colorectal cancer in patients with diabetes mellitus(糖尿病与结直肠癌患病危险关系的调查分析). World Chinese Journal of Digestology, 2007(01): p. 88-91.<br>● Yu, Z.G., et al., The Study on the Colorectal Cancer Related Risk Factors of 577 Cases(577 例大肠癌相关危险因素的研究). Chinese Journal of Clinical Gastroenterology, 2013. 25(02): p. 85-87.<br>● Nie, H.Y. and D.H. Zhu, Analysis of the risk factors of patients with coprectal cancer(糖尿病与结直肠癌发生的危险性分析). Laboratory Medicine and Clinic, 2018. 15(07): p. 915-917+920.<br>● Yang, G., et al.,Some diseases of digestive system and large bowel cancer. A population-based case-control study in Shanghai(消化道疾患与大肠癌关系的病例:对照研究). Tumor, 1996. 16(2).<br>● Luo, X.T., et al., Association between type 2 diabetes mellitus and colorectal cancer and characteristics of colorectal cancer complicated with type 2 diabetes mellitus(2 型糖尿病与结直肠癌的相关性及合并 2 型糖尿病的结直肠癌的特点). China Medicine, 2014. 9(3): p. 345-349.<br>● Pang, Y., et al., Diabetes, plasma glucose and incidence of colorectal cancer in Chinese adults: a prospective study of 0.5 million people. Journal of Epidemiology and Community Health, 2018. 72(10): p. 919-925.
Hypertension	3	● Wang, Z., et al., Associations Between Nonalcoholic Fatty Liver Disease and Cancers in a Large Cohort in China. Clinical Gastroenterology and Hepatology : the Official Clinical Practice Journal of the American Gastroenterological Association, 2021. 19(4).<br>● Hu, S.Q., Z. Tang, and M. Zhang, Investigation on the risks of colorectal cancer in patients with diabetes mellitus(糖尿病与结直肠癌患病危险关系的调查分析). World Chinese Journal of Digestology, 2007(01): p. 88-91.<br>● Li, X., et al., Metabolic Syndrome Components and the Risk of Colorectal Cancer: A Population-Based Prospective Study in Chinese Men. Frontiers In Oncology, 2019. 9: p. 1047.
History of schistosomiasis	6	● Wu, D.R., A case-control study on risk factors of colorectal cancer in Jiashan County(嘉善县大肠癌危险因素的病例对照研究). Journal of Practical Oncology, 1990. 5(2).<br>● Guo, Z.R., Schistosomiasis japonicum and colorectal cancer :Logistic regression model to explore the etiology of colorectal cancer(日本血吸虫病与大肠癌:Logistic 回归模型探讨大肠癌的病因). Chinese Journal of Epidemiology, 1987(1).<br>● Wang, X.H., et al., A case-control study of risk factors for colon cancer(结肠癌危险因素的病例对照研究). Chinese Journal of Cancer, 2001(09): p. 977-980.<br>● Yang, G., et al.,Some diseases of digestive system and large bowel cancer. A population-based case-control study in Shanghai(消化道疾患与大肠癌关系的病例:对照研究). Tumor, 1996. 16(2).<br>● Xu, Z. and D.L. Su, Schistosoma japonicum and colorectal cancer: an epidemiological study in the People's Republic of China. International Journal of Cancer, 1984. 34(3): p. 315-318.<br>● Qiu, D.C., et al., A matched, case-control study of the association between Schistosoma japonicum and liver and colon cancers, in rural China. Annals of Tropical Medicine and Parasitology, 2005. 99(1): p. 47-52.

Table S12 Summary information specific to each factor included in the overall meta-analysis.

Risk factor	Comparison	No. of studies	Case / Total	OR (95%CI)	P value	I^2(95%CI)	95%PI	Evidence grade
**Sociodemographic**								
Married	Married vs. Unmarried	4	1,234/ 202,153	0.78 (0.47, 1.30)	0.344	76.4 (35.2, 91.4)	(0.29, 2.12)	NS
Education	High level vs. Low level	4	6,212/ 642,582	0.82 (0.64, 1.05)	0.111	91.0 (84.7, 94.7)	(0.41, 1.64)	NS
Static occupation	Mental vs. Manual labor	6	1,392/ 132,286	1.54 (1.13, 2.10)	0.006	60.5 (18.0, 81.0)	(0.74, 3.20)	Weak evidence
**Anthropometrics**								
BMI	High vs. Low	10	4,034/ 164,497	1.37 (1.18, 1.59)	4.62E-05	65.8 (35.1, 81.9)	(0.92, 2.03)	Suggestive evidence
Waist hip rate	High vs. Low	4	2,630/ 295,341	1.27 (1.12, 1.45)	2.36E-04	33.7 (0.0, 74.9)	(1.03, 1.57)	Suggestive evidence
Triglyceride	High vs. Low	4	1,459/ 161,932	1.07 (0.98, 1.17)	0.158	43.9 (0.0, 81.3)	(0.92, 1.23)	NS
C-reactive protein	High vs. Low	3	879/ 189,538	1.33 (0.91, 1.93)	0.140	85.2 (56.6, 95.0)	(0.66, 2.66)	NS
**Lifestyle**								
Smoking	Ever vs. Never	26	9,555/ 190,527	1.19 (1.01, 1.40)	0.042	82.2 (76.1, 86.7)	(0.50, 2.85)	Weak evidence
Alcohol	Ever vs. Never	26	12,107/ 678,764	1.35 (1.21, 1.50)	5.13E-08	90.2 (87.7, 92.2)	(0.78, 2.34)	Suggestive evidence
Tea	Ever vs. Never	12	9,994/ 739,966	0.73 (0.62, 0.86)	9.87E-05	80.8 (69.7, 87.8)	(0.41, 1.30)	Suggestive evidence
Irregular bowel movement	> 1 time/d vs. 1 time/d	3	3,384/ 510,934	2.30 (1.18, 4.48)	0.015	78.7 (42.7, 92.0)	(0.61, 8.65)	Weak evidence
Sedentariness	Yes vs. No	8	1,882/ 4,733	1.44 (1.13, 1.84)	0.003	76.3 (57.6, 86.8)	(0.76, 2.73)	Weak evidence
Moderate physical activity	Moderate vs. Low intensity	4	1,822/ 4,875	1.01 (0.86, 1.19)	0.907	20.3 (0.0, 60.7)	(0.76, 1.34)	NS
Heavy physical activity	High vs. Low intensity	4	1,822/ 4,875	0.64 (0.44, 0.93)	0.020	20.3 (0.0, 60.7)	(0.24, 1.76)	Weak evidence
Exercise	Yes vs. No	9	3,210/ 189,508	0.67 (0.48, 0.95)	0.024	90.2 (83.6, 94.1)	(0.25, 1.81)	Weak evidence
**Dietary intake**								
Milk and dairy products	Highest vs. Lowest category	6	7,315/ 590,941	0.85 (0.59, 1.23)	0.390	94.2 (90.4, 96.5)	(0.33, 2.30)	NS
Fruit and vegetables	Highest vs. Lowest category	21	8,095/ 183,515	0.61 (0.51, 0.74)	1.80E-07	87.8 (83.5, 91.0)	(0.25, 1.47)	Suggestive evidence
Vegetables	Highest vs. Lowest category	19	7,591/ 110,071	0.60 (0.49, 0.73)	3.52E-07	87.7 (82.7, 91.3)	(0.25, 1.41)	Suggestive evidence
Fruits	Highest vs. Lowest category	5	2,675/ 95,644	0.62 (0.40, 0.96)	0.030	86.4 (72.6, 93.3)	(0.22, 1.76)	Weak evidence
Calcium	Highest vs. Lowest category	8	7,449/ 87,935	1.00 (1.00,1.00)	0.379	83.4 (73.5, 89.6)	(0.99, 1.00)	NS
Iron	Highest vs. Lowest category	3	3,808/ 7,796	1.01 (0.91,1.13)	0.805	15.8 (0.0, 87.1)	(0.88, 1.17)	NS
Znic	Highest vs. Lowest category	3	2,163/ 4,505	0.83 (0.59,1.16)	0.274	79.4 (45.0, 92.3)	(0.43, 1.61)	NS
Selenium	Highest vs. Lowest category	3	2,163/ 4,505	0.87 (0.69,1.09)	0.226	81.2 (51.0, 92.8)	(0.55, 1.36)	NS
Folate	Highest vs. Lowest category	4	3,763/ 8,052	0.79 (0.58, 1.08)	0.134	65.0 (0.0, 88.1)	(0.45, 1.40)	NS
Eggs	Highest vs. Lowest category	5	2,081/ 106,722	1.35 (1.22, 1.49)	1.13E-08	0.0 (0.0, 74.6)	(1.22, 1.49)	Highly suggestive evidence
All meat	Highest vs. Lowest category	10	2,928/ 109,523	1.38 (1.14, 1.67)	9.36E-04	56.4 (20.8, 76.0)	(0.81, 2.37)	Suggestive evidence
Red meat	Highest vs. Lowest category	8	2,499/79,220	1.36 (1.08, 1.73)	0.010	60.7 (21.4, 80.3)	(0.76, 2.46)	Weak evidence
Carotenoid	Highest vs. Lowest category	4	2,892/ 79,153	0.61 (0.49, 0.76)	7.33E-06	60.7 (21.4, 80.3)	(0.39, 0.95)	Suggestive evidence

Vitamin A	Highest vs. Lowest category	5	2,784/ 79,156	0.88 (0.66, 1.19)	0.409	85.3 (69.9, 92.8)	(0.44, 1.78)	NS
Vitamin E	Highest vs. Lowest category	4	2,582/ 78,550	0.62 (0.48, 0.80)	3.06E-04	51.0 (0.0, 82.0)	(0.38, 1.01)	Suggestive evidence
Vitamin C	Highest vs. Lowest category	5	3,099/ 79,776	0.66 (0.47, 0.92)	0.013	88.6 (78.9, 93.8)	(0.28, 1.53)	Weak evidence
VitaminB	Highest vs. Lowest category	3	3,618/ 80,020	0.64 (0.51, 0.80)	8.64E-05	70.3 (35.1, 86.4)	(0.39, 1.06)	Suggestive evidence
Carbohydrate	Highest vs. Lowest category	4	4,200/ 81,222	0.95 (0.74, 1.23)	0.720	50.2 (0.0, 81.7)	(0.60, 1.51)	NS
Protein	Highest vs. Lowest category	5	2,544/ 6,782	1.06 (0.88, 1.26)	0.554	59.1 (5.9, 82.3)	(0.73, 1.53)	NS
Fiber	Highest vs. Lowest category	16	8,547/ 90,991	0.66 (0.60, 0.74)	1.06E-13	90.5 (87.6, 92.8)	(0.42, 1.06)	Suggestive evidence
Aquatic product	Highest vs. Lowest category	10	3,899/ 153,508	0.97 (0.80,1.17)	0.730	88.2 (82.2, 92.2)	(0.50, 1.88)	NS
SFA	Highest vs. Lowest category	3	1,012/ 74,947	1.08 (0.93, 1.26)	0.315	0.0 (0.0, 89.6)	(0.93, 1.26)	NS
UFA	Highest vs. Lowest category	3	1,279/ 147,932	0.86 (0.64, 1.16)	0.322	70.9 (36.6, 86.7)	(0.42, 1.76)	NS
Beans and soy products	Highest vs. Lowest category	5	2,002/ 133,075	0.82 (0.68, 0.99)	0.037	62.6 (9.1, 84.6)	(0.56, 1.20)	Weak evidence
Fried food	Highest vs. Lowest category	6	1,014/ 3,669	2.22 (1.40, 3.52)	6.92E-04	93.2 (88.51, 96.0)	(0.67, 7.30)	Suggestive evidence
Smoked products	Highest vs. Lowest category	6	1,148/ 75,694	1.50 (1.08, 2.09)	0.016	94.4 (90.8, 96.6)	(0.63, 3.56)	Weak evidence
Salted food	Highest vs. Lowest category	13	4,328/ 112,800	1.76 (1.37, 2.26)	9.57E-06	81.8 (71.6, 88.4)	(0.75, 4.15)	Suggestive evidence
Roast food	Highest vs. Lowest category	3	1,658/ 75,858	2.40 (1.47, 3.91)	4.62E-04	82.0 (44.5, 94.2)	(0.97, 5.94)	Suggestive evidence
Cholesterol	Highest vs. Lowest category	3	746/ 74,280	1.00 (1.00,1.00)	0.570	71.1 (1.7, 91.5)	(1.00, 1.00)	NS
Total energy	Highest vs. Lowest category	4	1,677/ 76,763	1.00 (1.00,1.00)	0.274	39.7 (0.0, 77.7)	(1.00, 1.00)	NS
Total fat	Highest vs. Lowest category	3	1,983/ 4,796	1.06 (0.92,1.23)	0.424	22.9 (0.0, 88.2)	(0.85, 1.32)	NS
Oil type (animal oils)	Highest vs. Lowest category	7	1,058/ 3,295	1.89 (1.39, 2.56)	4.59E-05	79.3 (57.5, 89.9)	(0.95, 3.77)	Suggestive evidence
Greasy food	Highest vs. Lowest category	12	2,625/ 6,042	2.77 (1.95, 3.94)	1.50E-08	87.5 (79.9, 92.2)	(0.89, 8.65)	Highly suggestive evidence
DII	Highest vs. Lowest category	3	4,771/ 11,786	1.45 (1.30, 1.62)	4.43E-11	0.0 (0.0, 89.6)	(1.30, 1.62)	Convincing evidence
Well water	Yes vs. No	3	593/ 2,838,569	0.48 (0.15, 1.54)	0.217	96.4 (93.5, 98.1)	(0.04, 6.03)	NS
**Mental profile**								
History of mental stimulation	Ever vs. Never	5	2,976/ 72,313	1.86 (1.26, 2.76)	0.002	80.9 (58.9, 91.1)	(0.78, 4.44)	Weak evidence
Negative emotion	Ever vs. Never	5	1,393/ 5,644	3.07 (1.78, 5.30)	5.55E-05	79.4 (55.2, 90.6)	(0.86, 10.95)	Suggestive evidence
Bad mental state	Ever vs. Never	4	594/ 3,125	3.88 (2.27, 6.62)	6.69E-07	52.7 (0.0, 84.4)	(1.53, 9.85)	Weak evidence
Poor emotion management	Yes vs. No	3	338/ 878	2.51 (1.06, 5.92)	0.036	66.5 (0.0, 90.3)	(0.58, 10.88)	Weak evidence
Type A personality	Yes vs. No	4	1,033/ 3,884	1.48 (1.06, 2.06)	0.022	68.1 (7.4, 89.0)	(0.81, 2.69)	Weak evidence
**Personal history**								
Helicobacter pylori infection	Ever vs. Never	3	653/ 24,406	2.11 (1.40, 3.17)	3.36E-04	79.3 (34.2, 93.5)	(1.00, 4.45)	Weak evidence
History of hepatobiliary disease	Ever vs. Never	14	11,828/ 1,260,875	1.43 (1.23, 1.66)	2.87E-06	71.1 (51.1, 82.9)	(0.92, 2.22)	Suggestive evidence
Family history of cancer	Ever vs. Never	33	11,488/ 207,536	2.30 (1.93, 2.74)	1.07E-20	68.5 (55.2, 77.9)	(1.06, 4.99)	Suggestive evidence
Family history of CRC	Ever vs. Never	15	8,018/ 169,794	2.22 (1.92, 2.58)	5.16E-26	13.2 (0.0, 71.6)	(1.72, 2.87)	Convincing evidence
Diabetes	Ever vs. Never	11	9,853/ 582,242	1.67 (1.22, 2.29)	0.001	94.3 (91.6, 96.2)	(0.60, 4.65)	Weak evidence

Hypertension	Ever vs. Never	3	1,013/ 159,617	1.00 (0.84, 1.18)	0.968	0.0 (0.0, 89.6)	(0.84, 1.18)	NS
History of schistosomiasis	Ever vs. Never	6	4,022/ 9,668	2.13 (1.56, 2.90)	1.82E-06	33.8 (0.0, 73.4)	(1.26, 3.60)	Suggestive evidence

[1]**Abbreviations:** CRC, colorectal cancer; BMI, body mass index; UFA, unsaturated fatty acids; SFA, saturated fatty acids; DII, dietary inflammatory index; OR, odds ratio; CI, confidence interval; PI, prediction interval; NS, non-significant.

Table S13 Summary information specific to each factor included in the confined meta-analysis (restricted to high quality studies).

Risk factor	Comparison	No. of studies	Case / Total	OR (95%CI)	P value	I^2(95%CI)	95%PI	Evidence grade
**Sociodemographic**								
Education	High level vs. Low level	3	5,818/ 569,358	0.80 (0.59, 1.08)	0.140	93.5 (88.5, 96.3)	(0.37, 1.71)	NS
**Anthropometrics**								
Waist hip rate	High vs. Low	3	2,236/ 222,117	1.29 (1.10, 1.51)	0.002	48.6 (0.0, 83.0)	(0.98, 1.68)	Weak evidence
Triglyceride	High vs. Low	3	1,095/ 160,835	1.08 (1.01, 1.16)	0.029	23.9 (0.0, 92.1)	(0.99, 1.18)	Weak evidence
C-reactive protein	High vs. Low	3	879/ 189,538	1.33 (0.91, 1.93)	0.140	85.2 (56.6, 95.0)	(0.66, 2.66)	NS
**Lifestyle**								
Smoking	Ever vs. Never	4	1,645/ 80,473	1.26 (0.92, 1.75)	0.155	76.6 (43.1, 90.4)	(0.62, 2.56)	NS
Alcohol	Ever vs. Never	7	4,503/ 567,291	1.09 (0.96, 1.25)	0.189	79.1 (64.1, 87.8)	(0.76, 1.58)	NS
Tea	Ever vs. Never	3	2,766/ 586,195	0.84 (0.58, 1.20)	0.330	84.0 (51.8, 94.7)	(0.43, 1.64)	NS
Irregular bowel movement	> 1 time/d vs. 1 time/d	3	3,384/ 510,934	2.30 (1.18, 4.48)	0.010	78.7 (42.7, 92.0)	(0.61, 8.65)	Weak evidence
Exercise	Yes vs. No	4	1,548/ 113,381	0.56 (0.34, 0.93)	0.025	81.0 (50.4, 92.8)	(0.21, 1.46)	Weak evidence
**Dietary intake**								
Fruit and vegetables	Highest vs. Lowest category	8	4,490/ 70,112	0.50 (0.35, 0.73)	2.56E-04	93.1 (89.1, 95.7)	(0.17, 1.53)	Suggestive evidence
Vegetables	Highest vs. Lowest category	8	4,490/ 70,112	0.55 (0.39, 0.78)	7.09E-04	92.3 (87.5, 95.2)	(0.20, 1.55)	Suggestive evidence
Calcium	Highest vs. Lowest category	4	3,838/ 80,484	0.57 (0.44,0.74)	2.61E-05	54.4 (0.0, 84.9)	(0.36, 0.90)	Suggestive evidence
Folate	Highest vs. Lowest category	3	3,623/ 7,569	0.75 (0.54, 1.04)	0.088	71.0 (1.2, 91.5)	(0.42, 1.34)	NS
Red meat	Highest vs. Lowest category	5	1,034/ 3,030	1.60 (1.17, 2.19)	0.003	50.3 (0.0, 81.8)	(0.91, 2.81)	Weak evidence
Carotenoid	Highest vs. Lowest category	3	1,961/ 76,670	0.64 (0.47, 0.88)	0.005	69.4 (11.6, 89.4)	(0.35, 1.17)	Weak evidence
Fiber	Highest vs. Lowest category	5	3,873/ 80,577	0.39 (0.27, 0.57)	6.58E-07	71.6 (41.5, 86.2)	(0.17, 0.91)	Suggestive evidence
Aquatic product	Highest vs. Lowest category	3	632/ 73,715	1.14 (0.45,2.92)	0.780	86.2 (60.0, 95.2)	(0.19, 6.71)	NS
Smoked products	Highest vs. Lowest category	3	349/ 1,555	1.17 (0.93, 1.48)	0.190	75.8 (33.4, 91.2)	(0.76, 1.82)	NS
Roast food	Highest vs. Lowest category	3	1,658/ 75,856	2.40 (1.47, 3.92)	4.65E-04	82.0 (44.5, 94.2)	(0.97, 5.94)	Suggestive evidence
Oil type (animal oils)	Highest vs. Lowest category	3	527/ 2,016	1.42 (1.06, 1.91)	0.020	74.9 (16.8, 92.4)	(0.86, 2.35)	Weak evidence
Greasy food	Highest vs. Lowest category	3	461/ 922	2.67 (1.25, 5.71)	0.010	67.0 (0.0, 90.5)	(0.73, 9.76)	Weak evidence
**Mental profile**								
Poor emotion Management	Yes vs. No	3	338/ 878	2.51 (1.06, 5.92)	0.040	66.5 (0.0, 90.3)	(0.58, 10.88)	Weak evidence
**Personal history**								
History of hepatobiliary disease	Ever vs. Never	4	4,210/ 660,029	1.41 (1.08, 1.85)	0.011	73.4 (25.4, 90.5)	(0.84, 2.38)	Weak evidence
Family history of cancer	Ever vs. Never	6	968/ 75,368	2.85 (1.34, 6.07)	0.007	77.8 (50.7, 90.0)	(0.52, 15.45)	Weak evidence

[1]**Abbreviations:** OR, odds ratio; CI, confidence interval; PI, prediction interval; NS, non-significant.

Figure S1 Trends analysis in age-standardised incidence and mortality from 1990 to 2019: Data from GBD 2019.

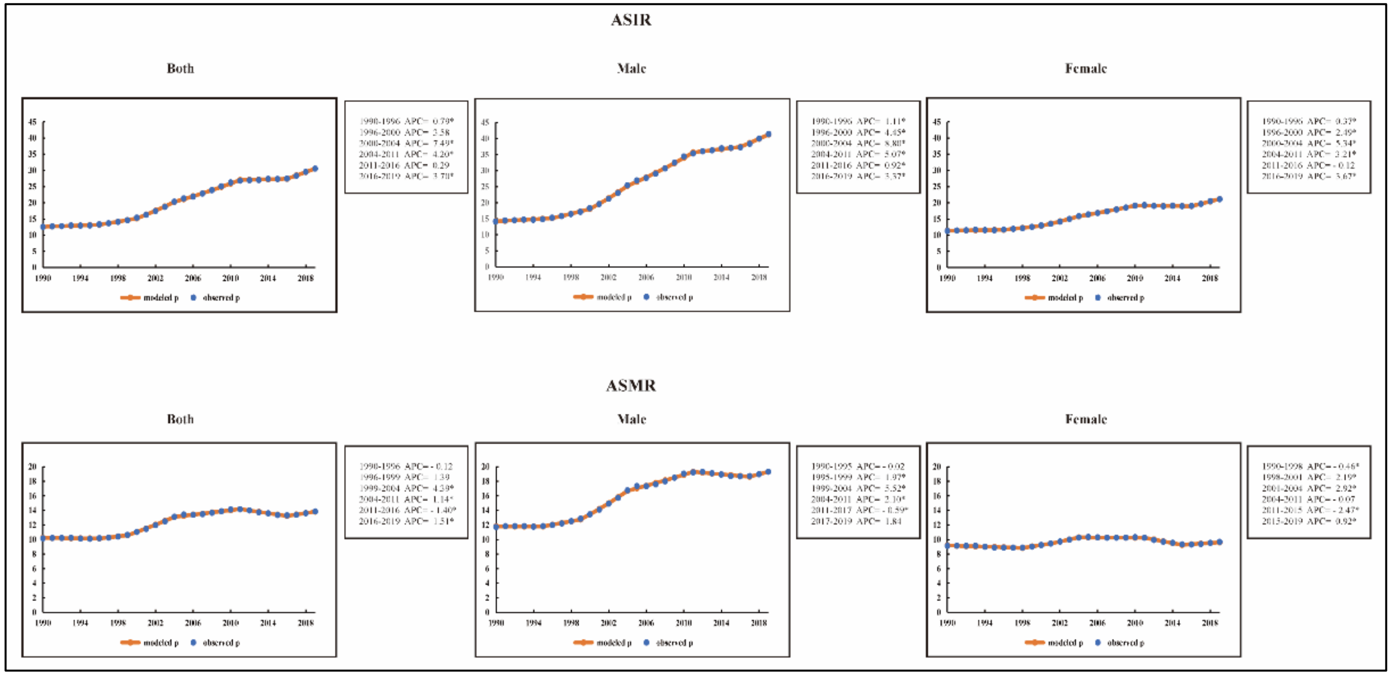

Figure S2 Trends analysis in age-standardised incidence from 1988 to 2012: Data from GLOBOCAN 2020 online database.

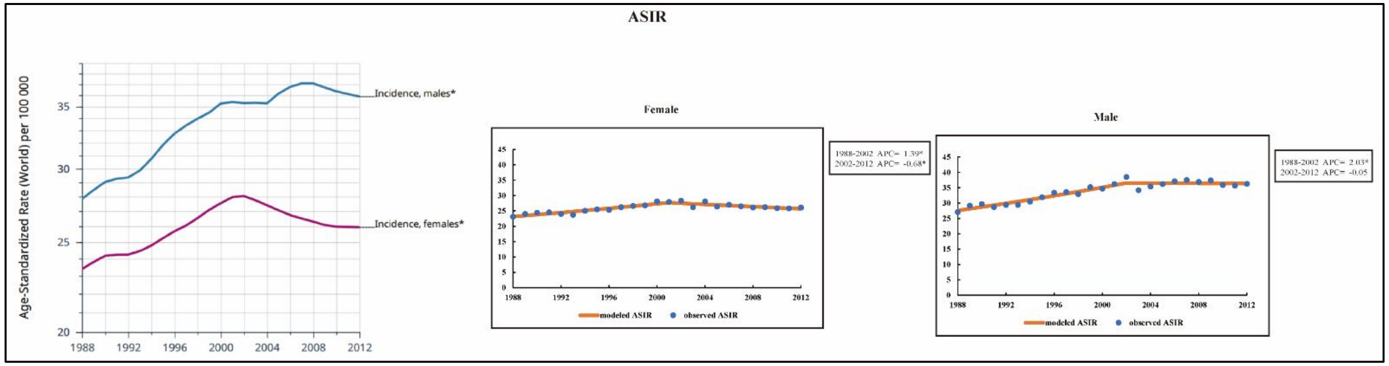

Figure S3 Trends analysis in age-standardised incidence and mortality rates by geographic partition from 1972 to 2020.

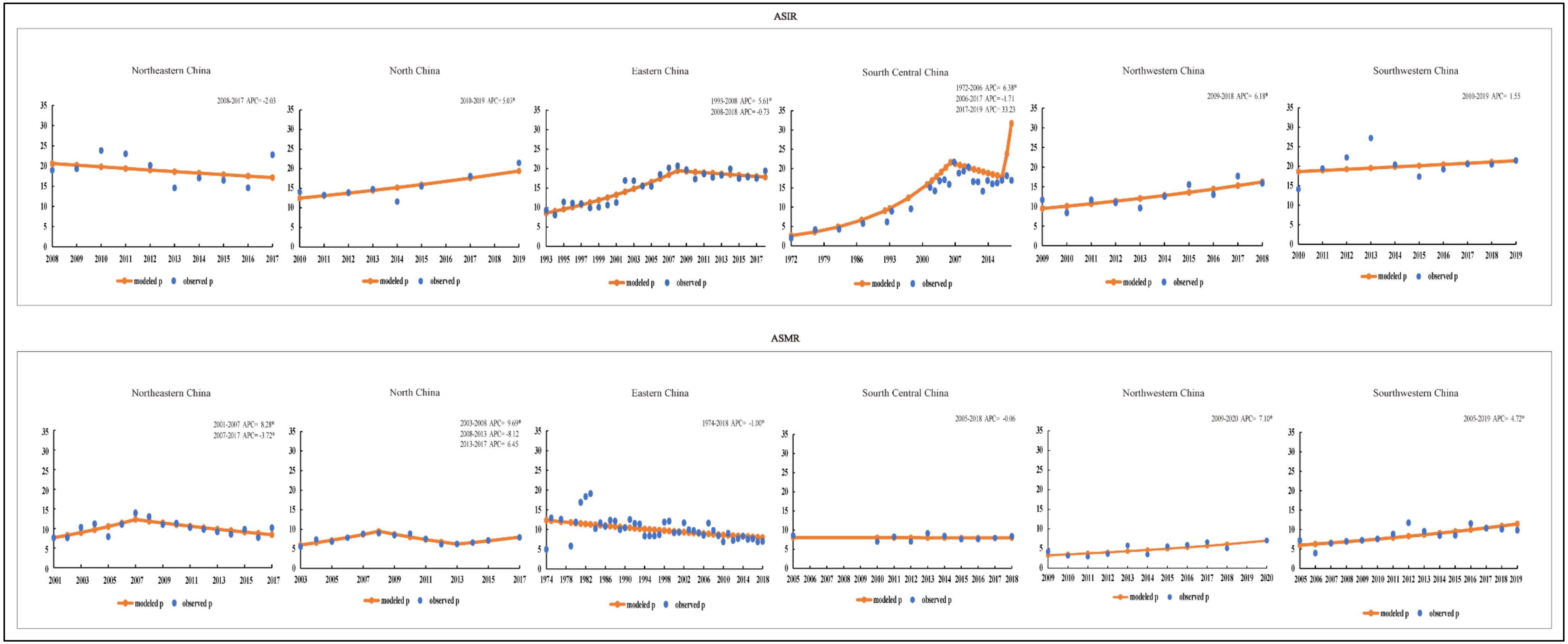

Figure S4 Trends analysis in age-standardised incidence and mortality rates by economic partition from 1972 to 2020.

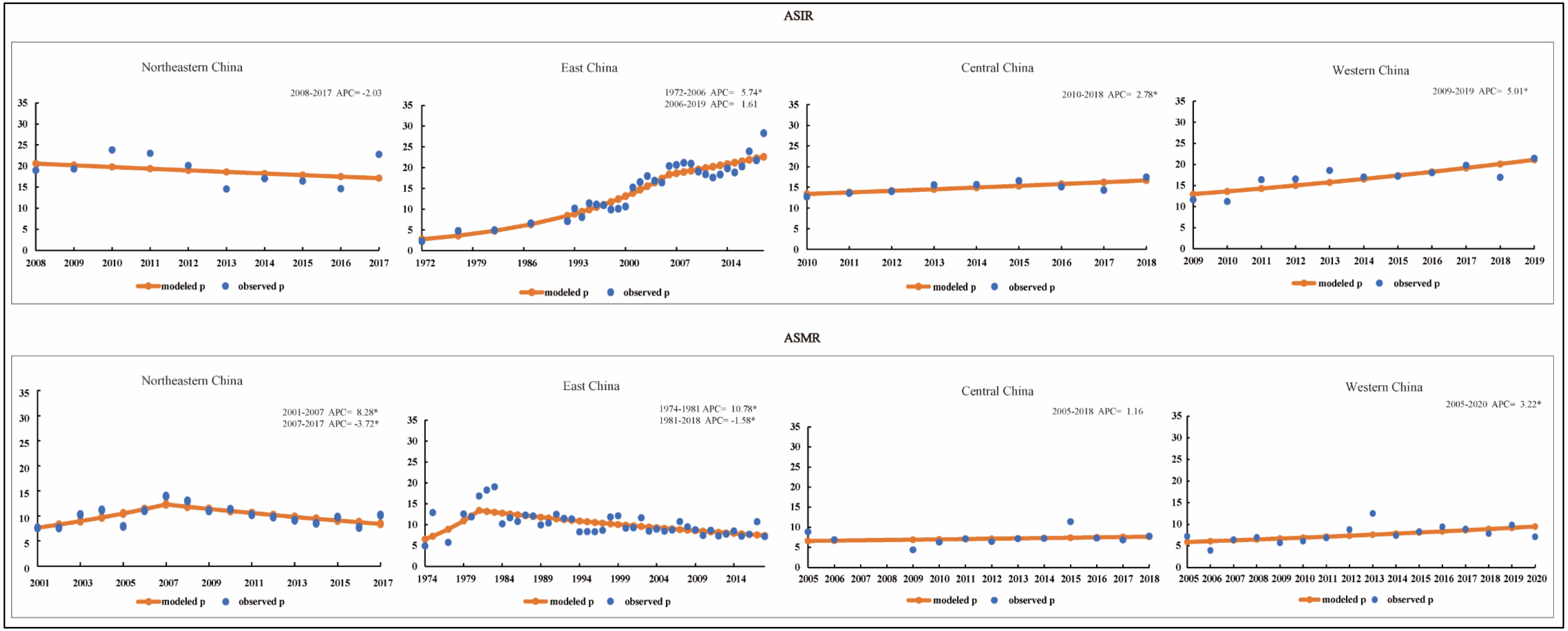

Supplement: Online Supplementary Document [file jogh-13-04096-s001.pdf]
